# Glycoconjugates of Mucochloric Acid—Synthesis and Biological Activity

**DOI:** 10.3390/ph16040525

**Published:** 2023-03-31

**Authors:** Katarzyna Żurawska, Daria Burdalska, Magdalena Skonieczna, Anna Byczek-Wyrostek, Anahit Dawicka, Anna Kasprzycka, Krzysztof Walczak

**Affiliations:** 1 Department of Organic Chemistry, Bioorganic Chemistry and Biotechnology, Faculty of Chemistry, Silesian University of Technology, Krzywoustego Street 4, 44-100 Gliwice, Poland; 2 Biotechnology Centre, The Silesian University of Technology, Krzywoustego Street 8, 44-100 Gliwice, Poland; 3 Department of Systems Biology and Engineering, The Silesian University of Technology, Akademicka Street 16, 44-100 Gliwice, Poland

**Keywords:** 3,4-dichloro-furan-2(5*H*)-one, 2*H*-pyrrol-2-one, click chemistry, glycoconjugate, anticancer activity

## Abstract

The pharmacological effects of the presence of a sugar moiety, 1,2,3-triazole ring and silyl groups in the structure of biologically active compounds have been extensively studied in drug design and medicinal chemistry. These components can be useful tools to tailoring the bioavailability of target molecules. Herein we present the study on the impact of the sugar substituent structure and triisopropylsilyl group presence on the anticancer activity of mucochloric acid (MCA) derivatives containing the furan-2(5*H*)-one or 2*H*-pyrrol-2-one core. The obtained results clearly indicated that tested compounds caused a significant decrease in cell viability of HCT116 and MCF-7 cell lines. MCF-7 cells indicate serious resistance toward investigated compounds in comparison with HCT116 cell line, it suggests that estrogen-dependent breast cancer cells are significantly less sensitive to the tested derivatives. Depending on the structure of the sugar, the type and site of connection with the furanone or 2*H*-pyrrol-2-one derivative and the presence of the silyl group, the selectivity of the compound towards cancer cells can be controlled. The obtained results may have an impact on the design of new furanone-based anticancer compounds.

## 1. Introduction

**Figure 1:**
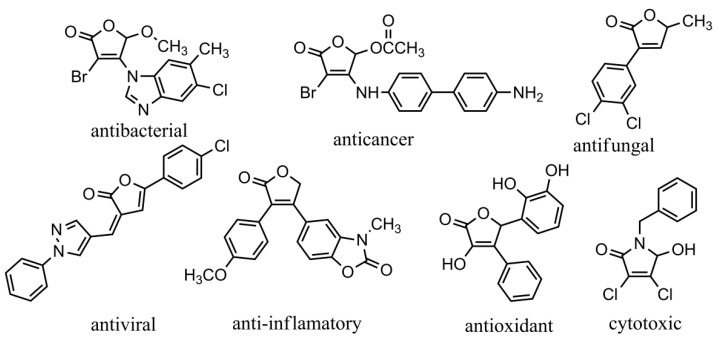
The structures of the structures based on furan-2(5*H*)-one and 2*H*-pyrrol-2-one with known biological activity.

The furanone scaffold is found in many complex natural products and exhibits diverse biological properties, such as antibacterial [1,2,3,4], anticancer [5,6,7,8,9,10], antifungal [11,12], antiviral [13], anti-inflammatory [14,15,16,17] and antioxidant [15,16,17,18] (Figure 1). In recent years, several derivatives, such as 3,4-dihalogeno-furan-2(5*H*)-one derivatives, have been obtained, which exhibited various anticancer activities [19,20]. Molecular targets for these derivatives were such key enzymes as kinases, COX-1, topoisomerase I or MDM2-p53 interaction. As a result of the reductive amination reaction of 3,4-dihalogeno-furan-2(5*H*)-one derivatives in the presence of various amines, highly functionalized α,β-unsaturated α-halogeno-β-aryl-γ-butyrolactams were obtained [21]. These compounds have been shown to exhibit cytotoxic effects [9] and increase the sensitivity of tumor cells to oncogenic viruses [22]. Additionally, as we have shown in our previous work [19], the introduction of a silyl group into the structure of 3,4-dihalogeno-furan-2(5*H*)-one increases the cytotoxicity of the tested compound with reference to the initial structure.

Carbohydrates and their glycoconjugates are involved in many biological processes and play an important role in various diseases. The structure of carbohydrates, including the presence of functional groups, allows numerous structural modifications leading to creation of new compounds with biological activity as well as enhancing the activity of existing drugs. A good example of this strategy are carbohydrate-based metallo-drugs such as platinum carbohydrate complexes [23]. Glycosylation of peptides can protect against proteolysis and improve aqueous solubility. Sugar residues are most often attached to biologically active compounds in order to increase their bioactivity, improve their physical and chemical properties or target their action [24]. Many of them are subjected to further modifications in order to obtain better activity and selectivity compared to the parent structure. Carbohydrate-based drugs have e.g., antibacterial [25], antidiabetic [26,27], anticancer and antiviral effects, and act as enzyme inhibitors [28,29,30,31].

In continuation of our studies on the synthesis and biological activity of 3,4-dihalogeno-furan-2(5*H*)-one derivatives [3,19,20,32], in this paper, we describe the synthesis and evaluation of anticancer activity of a group of novel glycoconjugates of 3,4-dichloro-furan-2(5*H*)-one and 2*H*-pyrrol-2-one with sugar residues.

## 2. Results and Discussion

### 2.1. Synthesis

**Scheme 1:**
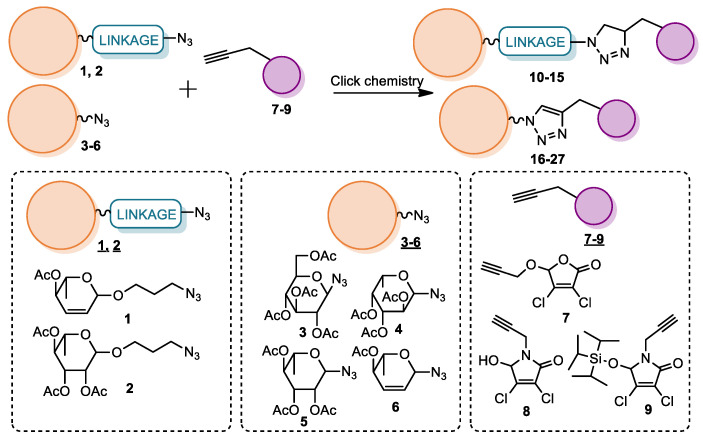
Analogs of mucochloric acid joined with sugar derivatives via 1,2,3-triazole linker in click chemistry reaction.

Derivatives of 3,4-dichloro-furan-2(5*H*)-one and 2*H*-pyrrol-2-one were joined to appropriate sugar derivative via *O*-CH_2_-CH_2_-CH_2_-1,2,3-triazole linker or 1,2,3-triazole ring, which was built directly on anomeric carbon atom (Scheme 1). 1,2,3-Triazole is an important heterocycle in medicinal chemistry, often use as a convenient coupler joining two parts into final product. It is formed via 1,3-dipolar addition of appropriate azido derivatives (1,3-dipole) with corresponding propargyl fragment of 1,3-dipolarophile. It occurs in many compounds with anticancer activity, inhibiting various enzymes such as EGFR [33,34,35], VEGFR [36] and PARP [37].

The synthesis strategy involves several steps. The first one leads to the sugar derivatives with an azide group capable of participating in click chemistry reactions (Table 1). Depending on the structure of sugar component, different approaches to synthesize azido derivatives were applied, imposed by optimalisation of adducts yield and anomers ratio. Addition of 3-azidopropan-1-ol to 3,4-di-*O*-acetyl-L-rhamnal in a presence of FeCl_3_ as catalyst provided **1** as a result of Ferrier rearrangement reaction in 87% yield [38]. Compound **1** is a mixture of isomers (α:β = 1:6). Treatment of per-*O*-acetyl-L-rhamnose with 3-azidopropan-1-ol, in the presence of BF_3_OEt_2_ provided adduct **2** as a single β anomer with 82% yield [39]. Derivatives **3**–**5** were obtained in reaction of appropriate per-*O*-acetylated sugars with azidotrimethylsilane (TMSN_3_), in anhydrous methylene chloride, in the presence of SnCl_4_ as catalyst [40]. Compound **3** was obtained as an anomer β with 97% yield, compound **4** as an anomer β with 98% yield, and compound **5** as a mixture of isomers (α:β = 1:8) with 97% yield. Reaction of TMSN_3_ with 3,4-di-*O*-acetyl-L-rhamnal in the presence of I_2_ produces Ferrier rearrangement product **6** as a mixture of isomers (α:β = 1:1.5), with 78% yield [41].

**Scheme 2:**
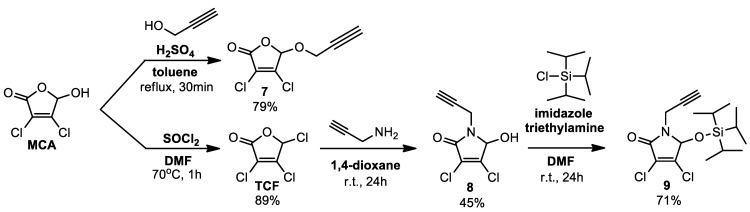
Synthesis of 3,4-dichloro-furan-2(5*H*)-one and 2*H*-pyrrol-2-one derivatives.

In the second step of synthesis, 3,4-dichloro-furan-2(5*H*)-one and 2*H*-pyrrol-2-one derivatives containing a propargyl group capable of participating in click chemistry reactions with sugar derivatives were synthesized (Scheme 2).

Propargyl derivative of 3,4-dichloro-furan-2(5*H*)-one (**7**) was obtained in reaction of mucochloric acid with propargyl alcohol in a presence of catalytic amounts of H_2_SO_4_ in 79% yield [22]. To obtain propargyl derivative of 2*H*-pyrrol-2-one (**8**), mucochloric acid (MCA) was transformed into 3,4,5-trichloro-furan-2(5*H*)-one (TCF) in reaction with thionyl chloride in a presence of DMF [22]. Next, 3,4,5-trichloro-furan-2(5*H*)-one reacted with propargylamine in 1,4-dioxane to obtain **8** in 45% yield [22]. Finally, *N*-propargyl-3,4-dichloro-5-hydroxy-2*H*-pyrrol-2-one **8** reacted with triisopropylsilyl chloride in the presence of imidazole and trimethylamine to obtain product **9** in 71% yield [42,43].

**Scheme 3:**
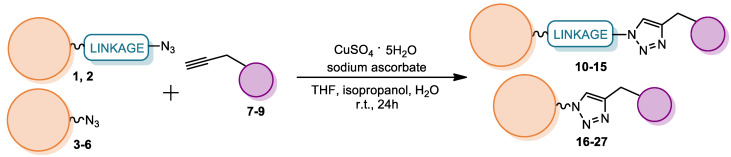

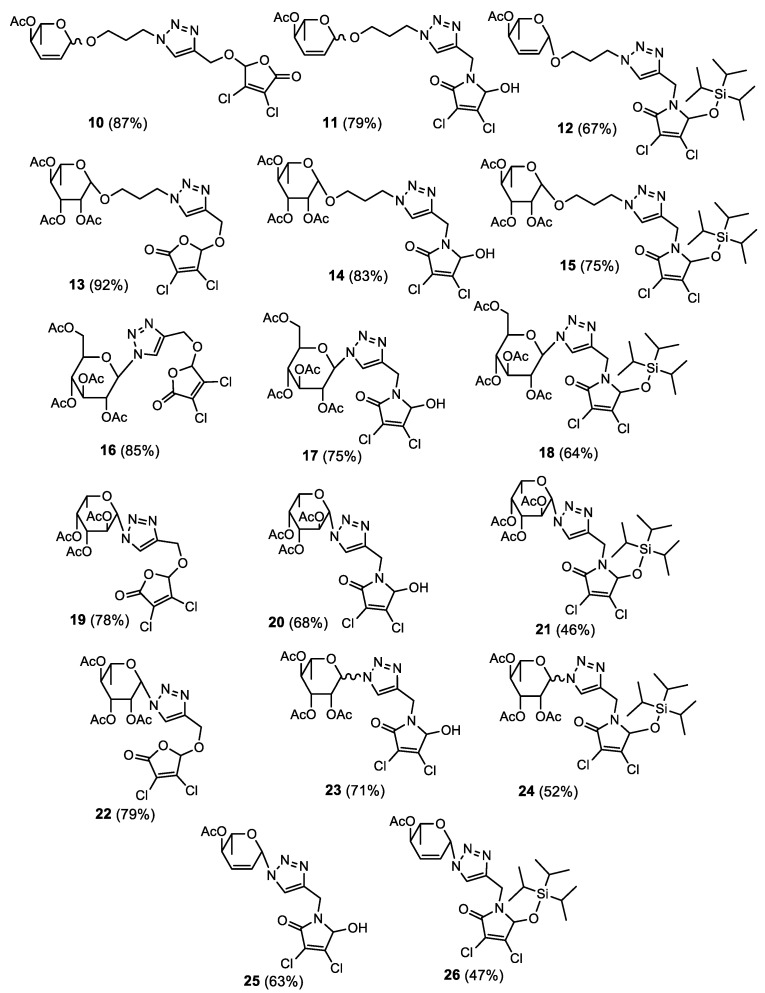
Synthesis of **10**–**26** in click chemistry reaction conditions.

In the third, final step, the desired derivatives **10**–**26** were obtained with high yield by Copper(I)-catalyzed azide-alkyne cycloaddition (CuAAC) starting from appropriate sugar azides **1**–**6** and propargyl derivatives of 3,4-dichloro-furan-2(5*H*)-one **7** or 2*H*-pyrrol-2-one **8** and **9** (Scheme 3). Structures of all prepared compounds were confirmed by analysis of ^1^H and ^13^C NMR spectra.

### 2.2. Anticancer Screening

#### 2.2.1. Cytotoxicity and Anticancer Activities

The 72 h in vitro MTT assay, performed on HCT116 and MCF-7 cancer cell lines, allowed the assessment of cell viability and IC_50_ parameter for tested compounds. IC_50_ for cell viability was described with a dose of the drug, with 50% reduction in the whole population when compared to the untreated controls. Obtained results allow for comparison of different compounds using the same or different targeted cell lines. The calculated IC_50_ parameters for tested compounds are presented in Table 2. As positive control, MCA was used with IC_50_ value determined on the same cell lines in previous research [20]. Cells survival fractions (SF) followed by 72 h MTT viability assay are presented in Supplementary File S1.

Based on the obtained results of the cytotoxicity, chemical modifications did not always improve the drug activity against cancer cell lines. The compounds **10**, **11**, **15**, **17**, **19**, **20** and **23** were not active at all, or reduced viability of only one of used cell lines, HCT116 or MCF-7, respectively. Such results may be explained by the modification in the compound structure, e.g., the presence of a sugar residue may facilitate the introduction of the compound into the cell, and the presence of a 1,2,3-triazole ring may increase cytotoxicity. Compound **12** showed high cytotoxicity against both colorectal and breast cancer cell lines, with the IC_50_ 9.1 ± 2.4 and 10.8 ± 0.7 μM, respectively. Other tested compounds exhibit moderate toxicity, but at doses over 10 μM.

Analysing the relationship between compound structure and activity against selected cancer cell lines, a significant improvement in the cytotoxicity was observed when the molecule contains 2,3-unsaturated sugar fragment (**10**, **11**, **12**, **25** and **26**). The smallest effect is observed in the case of derivatives containing L-fucose in their structure (**19**, **20** and **21**). Making a further comparison of the obtained derivatives, it can be concluded that an attachment of the sugar moiety through the nitrogen atom in the furan-2(5*H*)-one ring has a negligible effect on increasing cytotoxic properties. Moreover, the pattern of the sugar rest addition by the nitrogen/oxygen atom and the distance from the furan-2(5*H*)-one ring were not significant for improving the cytotoxic activity of the tested derivatives. However, it is noticeable that the compounds containing a silyl substituent in their structure (**12**, **18**, **21**, **24** and **26**) significantly inhibit the proliferation of cancer cells. More detailed cytotoxic effects are presented as particular HCT116 and MCF cells viability graphs in Supplementary File (Figure S1).

#### 2.2.2. Inhibition of Cell Cycle and Pro-Apoptotic Action

The mechanism of action of anticancer drugs may be based on cell cycle inhibition (cytostatic action), inducing apoptosis, cellular programmed death or rapid necrosis. To confirm the mechanism of action of the tested derivatives in cancer cells, a cycle using flow cytometry techniques was applied. The amount of DNA in a cell is variable and dependent on single stage of the cycle. In eukaryotic cells, there are two main phases, i.e., interphase and mitosis, in which the amount of nuclear DNA content is single (in human diploid cells 2n, which means a set of 46 chromosomes), or doubled after replication (S phase), in which mitosis begins (tetraploid cells 4n, with 46 chromosomes doubled into 92 sister chromatids) [44]. DNA staining with specific nuclear dyes such as propidium iodide (PI) allowed an easily counting and distinguishing between cells with a different DNA–nucleus contents. Using flow cytometry and appropriate analysis gating, the cell cycle phases can be matched: subG1 (apoptotic and necrotic cells); G0/G1 phase (2n diploid cells, also called mononuclear cells); S phase (replication with DNA synthesis; DNA content more than 2n); G2/M phase (4n tetraploids cells, with doubled DNA content in nucleus). The cells with disturbed and uncontrolled replication and damaged mitosis present polyploid fraction (DNA contend above 4n).

**Figure 2:**
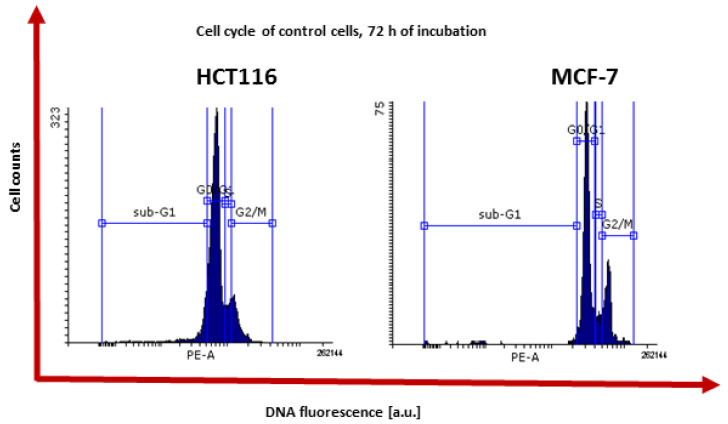
Cell cycle distribution in control HCT116 (**left**) and MCF-7 (**right**) cells after 72 h of incubation. DNA stained with PI [100 µg/mL] showed cells in cell cycle phases: subG1 (with the lowest content of DNA, <2n); G0/G1 (2n); S (>2n) and G2/M (4n).

Figure 2 shows histograms of typical DNA content in HCT116 and MCF-7 control cells, after 72 h of incubation.

**Figure 3:**
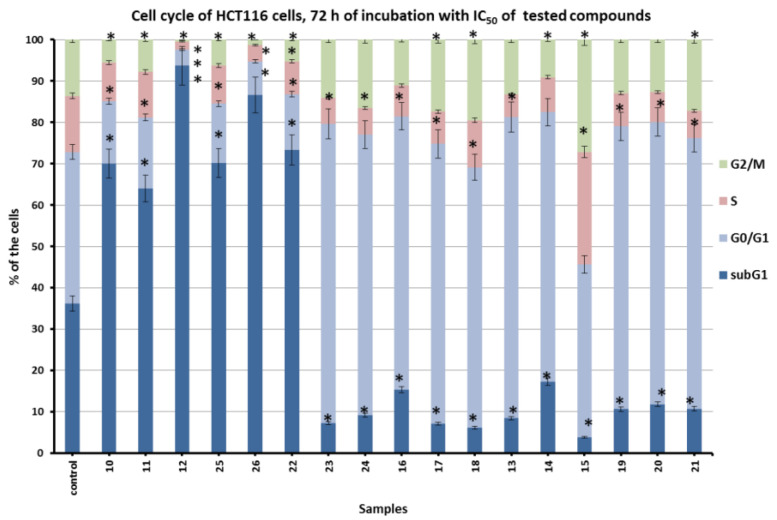
Cell cycle distribution in HCT116 cells after 72 h of incubation with tested compounds at IC_50_ dose or 100 µM for no effective compounds based on Table 2. The subG1 fraction represents apoptotic and dead cells. Results presented as means from 3 experiments, ±SD. Statistical significance indicated by star; evaluated by T-test, where *p* < 0.05.

**Figure 4:**
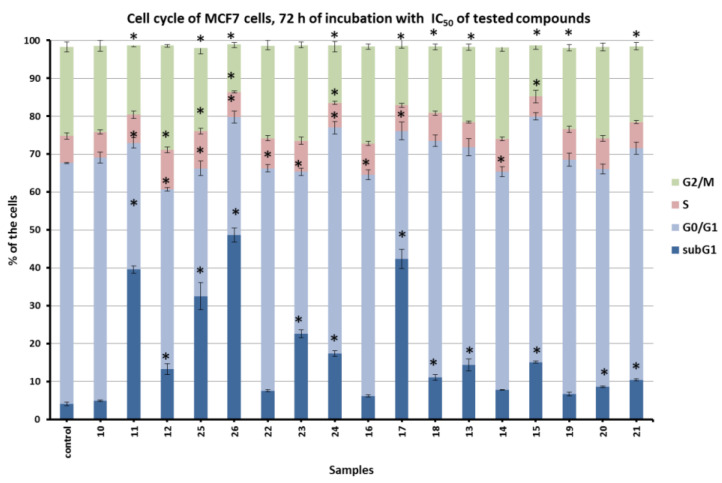
Cell cycle distribution in MCF-7 cells after 72 h of incubation with tested compounds at IC_50_ dose or 100 µM for no effective compounds, based on Table 2. The subG1 fraction represents apoptotic and dead cells. Results presented as mean of 3 experiments ± SD. Statistical significance indicated by star; evaluated by T-test, where *p* < 0.05.

The obtained results of the cell cycle analysis confirm the cytotoxic effects for most of the tested derivatives (Figure 3 and Figure 4), while their divergence for both cell lines may indicate tissue-dependent effects presented on HCT116 or MCF-7 cells, respectively. Based on the graphs, it can be seen that for both HCT116 and MCF-7 cell lines, an impact on subG1 phase elevation can be observed compared to untreated control (Figure 3 and Figure 4). An increase in the subG1 phase may indicate activation of programmed cell death, i.e., apoptosis and/or uncontrolled and rapid necrosis. Such aim of action confirms potentially pro-apoptotic effect of the tested compounds against HCT116 cancer cell line after addition of **10**, **11**, **12**, **25**, **26**, **22** (Figure 3), and MCF-7 cells after addition of **11**, **12**, **17**, **23**, **24**, **25**, **26** (Figure 3), respectively.

In contrast, for the HCT116 cell line, a slight increase in cell number was observed in the G0/G1 phase for compounds **13**, **14**, **16**, **17**, **18**, **19**, **20**, **21**, **23** and **24**, which may appear on a cell cycle blockade, probably because of the disruption of cell cycle checkpoints (Figure 3). Upon further analysis of the tested compounds, no cell growth was observed in the phases G0/G1 and S, with the exception of the 15 treatment for the HCT116 cells, where it significantly affected S-phase elevation compared to the untreated control (Figure 3).

Based on the results obtained on the cell cycle distribution in MCF-7 cells, after 72 h of treatment, a visible increase in apoptotic cells was observed in the subG1 fraction, followed by slight increase only for a few compounds, as confirmed by the cytotoxicity assay (Figure 4). The subG1 fraction increased after the addition of **11**, **12**, **13**, **15**, **17**, **18**, **20**, **21**, **23**, **25** and **26** (Figure 4); however, the effect on MCF-7 cell line was not so spectacular as on HCT116 cell line. Decreased G0/G1 fraction in most of the treatments on MCF-7 cells was the result of apoptotic fraction formation rather than cytostatic cell cycle blockade.

## 3. Conclusions

Glycoconjugate derivatives of 3,4-dichloro-furan-2(5*H*)-one and 2*H*-pyrrol-2-one were obtained and their in vitro biological activity was tested. The click chemistry approach in a simple, easy and inexpensive way leads to products with high yield and purity, which does not deteriorate the enantiomeric purity of the starting compounds.

The obtained results clearly indicated that tested compounds caused a significant decrease in cell viability of HCT116 and MCF-7 cell lines. When comparing the results for selected cell lines, it can be seen that the IC_50_ values on the MCF-7 cell line are much higher or show not activity at all in comparison to the HCT116 cell line, indicating that estrogen-dependent breast cancer cells are significantly less sensitive to the tested derivatives.

Analysing the structures of the tested compounds in terms of the structure of the sugar molecule, among the 3,4-dichloro-furan-2(*5H*)-one derivatives, the glucose derivative was the most cytotoxic compound, while among the 2*H*-pyrrole-2-one derivatives, the 4-*O*-acetyl-2,3,6-trideoxy-L-*erythro*-hex-2-eno pyranoside proved to be the most cytotoxic. These differences may be due to interactions with other molecular targets in the cell. It was also observed that increasing the distance of the sugar unit from the 1,2,3-triazole ring decreases the IC_50_ value of the tested compounds. The best IC_50_ results were obtained for derivatives **12**, **21** and **26** containing a silyl substituent in their structure, which may result from an increase in the lipophilicity of the compounds, and thus improve their bioavailability.

Performed investigation delivers a preliminary assessment of the anticancer effect of furanone derivatives and are a start point for further research focused on optimization of the structure and determination of the molecular target.

## 4. Experimental Section

### 4.1. General Information

All solvents were purified and dried according to standard methods before use. Reagents were purchased from commercial sources (Merck, Darmstadt, Germany; Acros Organics, Geel, Belgium; Alpha Aesar, Haverhill, USA and Sigma-Aldrich, Taufkirchen, Germany) and were used without further purification. Analytical thin layer chromatography (TLC) was performed on precoated plates of silica gel 60 F_254_ (Merck, Darmstadt, Germany) and visualized by exposure to ultraviolet light, or a 10% solution of sulphuric acid in ethanol. Chromatographic purification was performed on silica gel 60 (Merck, Darmstadt, Germany, particle size 0.063–0.2 mm). The ^1^H-NMR and ^13^C-NMR spectra were recorded on an Agilent 400 MHz or Varian 600 MHz spectrometer in DMSO-*d_6_* or CDCl_3_ using tetramethylsilane (TMS) as an internal standard. Mass spectra were recorded at ESI Mass Spectrometer ABSciex System 4000 QTRAP^®^ at positive mode of ionization. Optical rotations were measured with a JASCO P-2000 polarimeter using a sodium lamp (589.3 nm) at room temperature.

### 4.2. Chemistry

#### 4.2.1. Synthesis of Sugar Azides

1-(3-Azidopropyloxy)-4-*O*-acetyl-2,3,6-trideoxy-L-*erythro*-hex-2-enopyranoside **1**

To a solution of 3,4-di-*O*-acetyl-L-rhamnal (200 mg, 0.934 mmol) dissolved in DCM (2 mL), FeCl_3_ (15.1 mg, 0.093 mmol) and 3-azidopropan-1-ol (100 µL, 1.028 mmol) were added. The reaction was stirred at room temperature on a magnetic stirrer for 1 h. After completion of the reaction, monitored by TLC (toluene:EtOAc (2:1, *v*/*v*)), the reaction mixture was quenched with saturated solution of NaHCO_3_ (2 × 10 mL), and then aqueous phase was extracted with EtOAc (2 × 10 mL). Combined organic phases were dried over anhydrous MgSO_4_, filtered off. Removal of the solvent under diminished pressure followed by purification on silica gel using *n*-hexane:EtOAc (5:1, *v*/*v*) afforded product in the form of yellowish syrup, with 87% yield (206.8 mg), which is a mixture of isomers (α:β = 1:6); R_f_ = 0.76 (toluene:EtOAc 2:1). ^1^H NMR (600 MHz, CDCl_3_): *δ* 5.85–5.87 (m, 1H, H-2/3), 5.78–5.81 (m, 1H, H-2/3), 5.05–5.07 (m, 1H, H-1), 4.96 (m, 1H, H-4), 3.94 (dq, *J* = 6.6 Hz, 9.0 Hz, 1H, H-5), 3.87 (dt, *J* = 6.0 Hz, 9.6 Hz, -CH-), 3.56 (dt, *J* = 6.6 Hz, 10.2 Hz, -CH-), 3.40–3.43 (m, 2H, -CH_2_-), 2.09 (s, 3H, OAcα), 2.08 (s, 3H, OAcβ), 1.89 (qi, *J* = 6.6 Hz, 2H, -CH_2_-), 1.31 (d, *J* = 6.6 Hz, 3H, H-6β), 1.23 (d, *J* = 6.6 Hz, 3H, H-6α); ^13^C NMR (150 MHz, CDCl_3_): 170.6, 129.9, 127.7, 94.6, 71.0, 65.2, 65.1, 48.6, 29.4, 21.2, 18.1; MS(ESI): calcd. for C_11_H_17_N_3_O_4_ ([M+Na]^+^): *m*/*z* 278.1, found: *m*/*z* 278.5.

1-(3-Azidopropyloxy)-2,3,4-tri-*O*-acetyl-β-L-rhamnopyranoside **2**

To a solution of 1,2,3,4-tetra-*O*-acetyl-L-rhamnose (500 mg, 1.505 mmol) in DCM (15 mL), 3-azidopropan-1-ol (417 µL, 4.514 mmol) was added. The reaction mixture was stirred for 10 min at 0 °C, then BF_3_OEt_2_ (2 µL, 0.020 mmol) was added dropwise. The reaction was stirred for 2 h in 0 °C, and at room temperature for 8 h. After completion of reaction, monitored by TLC (toluene: EtOAc (2:1, *v*/*v*)), the reaction mixture was quenched with saturated solution of NaHCO_3_ (2 × 10 mL), and then aqueous phase was extracted with DCM (2 × 10 mL). Combined organic phases were dried over anhydrous MgSO_4_ and filtered off. Removal of the solvent in vacuo, followed by purification on silica gel using toluene: EtOAc (100:1, *v*/*v*) afforded product in the form of yellowish syrup, with 82% yield (460.6 mg) as an anomer β, α^24^_D_ = −38.5 (c = 1.0, CHCl_3_); 2); R_f_ = 0.62 (toluene:EtOAc 2:1). ^1^H NMR (400 MHz, CDCl_3_): *δ* 5.29–5.23 (m, 2H, H-2, H-3), 5.07 (t, 1H, *J* = 10.0 Hz, H-4), 4.73 (d, 1H, *J* = 1.6 Hz, H-1β), 3.75–3.89 (m, 2H, H-5, -CH_a_-), 3.46–3.53 (m, 1H, -CH_b_-), 3.43 (t, 2H, *J* = 6.4 Hz, -CH_2_-), 2.15 (s, 3H, OAc), 2.05 (s, 3H, OAc), 1.99 (s, 3H, OAc), 1.81–1.93 (m, 2H, -CH_2_-), 1.23 (d, 3H, *J* = 6.0 Hz, H-6); ^13^C NMR (100 MHz, CDCl_3_): *δ* 170.3, 170.1, 170.1, 97.7, 71.2, 69.9, 69.2, 66.7, 64.7, 48.3, 28.9, 21.1, 20.9, 20.9, 17.6; MS(ESI): calcd. for C_15_H_23_N_3_O_8_ ([M+H]^+^): *m*/*z* 373.1, found: *m*/*z* 373.5.

**General procedure A**

To per-*O*-acetyl-β-D-sugar (1.0 eq) dissolved in dry DCM (5 mL), TMSN_3_ (4.0 eq) and SnCl_4_ (1M SnCl_4_ in DCM, 0.26 eq) were added under argon atmosphere. The reaction was carried at room temperature until complete conversion of the sugar substrate (24 h). Then, the mixture was diluted with DCM (2 × 15 mL) and washed with saturated aqueous solution of NaHCO_3_ (2 × 15 mL), water (2 × 15 mL) and brine (2 × 15 mL). The organic phase was dried with MgSO_4_, filtered and concentrated on a rotary evaporator resulting in products **3**–**5**.

1-Azido-2,3,4,6-tetra-*O*-acetyl-β-D-glucopyranoside **3**

Obtained according to General Procedure A starting from 1,2,3,4,6-penta-*O*-acetyl-β-D-glucopyranose (500 mg, 1.281 mmol), TLC: *n*-hexane:toluene:EtOAc (3:3:4, *v*/*v*/*v*); yield: 97% (463.7 mg), product was obtained as white solid; m.p.: 125.2–126.9 °C as an anomer β, α^24^_D_ = −23.9 (c = 1.0, CHCl_3_); R_f_ = 0.57 (*n*-hexane:toluene:EtOAc 3:3:4). ^1^H NMR (600 MHz, CDCl_3_): *δ* 5.22 (t, 1H, *J* = 9.6 Hz, H-2), 5.11 (t, 1H, *J* = 9.6 Hz, H-3), 4.96 (t, 1H, *J* = 9.0 Hz, H-4), 4.65 (d, 1H, *J* = 9.0 Hz, H-1), 4.28 (dd, 1H, *J* = 4.8 Hz, 12.0 Hz, H-6a), 4.18 (dd, 1H, *J* = 1.8 Hz, 12 Hz, H-6b), 3.79–3.81 (m, 1H, H-5), 2.11 (s, 3H, OAc), 2.08 (s, 3H, OAc), 2.04 (s, 3H, OAc), 2.01 (s, 3H, OAc); ^13^C NMR (150 MHz, CDCl_3_): *δ* 170.7, 170.2, 169.4, 169.3, 88.1, 74.2, 72.8, 70.8, 68.0, 61.8, 20.8, 20.7, 20.7; MS(ESI): calcd. for C_14_H_19_N_3_O_9_ ([M+Na]^+^): *m*/*z* 396.1, found: *m*/*z* 396.3.

1-Azido-2,3,4-tri-*O*-acetyl-β-L-fucopyranoside **4**

Obtained according to General Procedure A starting from 1,2,3,4-tetra-*O*-acetyl-L-fucopyranose (500.0 mg, 1.505 mmol), TLC: toluene:EtOAc (2:1, *v*/*v*); yield: 98% (464.9 mg), product was obtained as colorless, solidifying oil as an anomer β, α^24^_D_ = 22.7 (c = 1.0, CHCl_3_); R_f_ = 0.78 (toluene:EtOAc 2:1). ^1^H NMR (600 MHz, CDCl_3_): *δ* 5.27 (d, *J* = 2.4 Hz, 1H, H-3), 5.13 (dd, *J* = 9.0 Hz, 9.6 Hz, 1H, H-4), 4.99 (dd, *J* = 3.0 Hz, 10.2 Hz, 1H, H-2), 4.59 (d, *J* = 8.4 Hz, 1H, H-1), 3.92 (q, *J* = 6.0 Hz, 12.0 Hz, 1H, H-5), 2.19 (s, 3H, OAc), 2.09 (s, 3H, OAc), 1.99 (s, 3H, OAc), 1.26 (d, *J* = 6.0 Hz, 1H, H-6); ^13^C NMR (150 MHz, CDCl_3_): *δ* 170.4, 170.0, 169.4, 88.2, 71.5, 71.1, 69.9, 68.9, 20.6, 20.6, 20.5, 16.0; MS(ESI): calcd. for C_12_H_17_N_3_O_7_ ([M+Na]^+^): *m*/*z* 338.1, found: *m*/*z* 338.3.

1-Azido-2,3,4-tri-*O*-acetyl-L-rhamnopyranoside **5**

Obtained according to General Procedure A starting from 1,2,3,4-tetra-*O*-acetyl-L-rhamnopyranose (500.0 mg, 1.505 mmol), TLC: *n*-hexane:EtOAc (1:1, *v*/*v*); yield: 97% (460.6 mg), product was obtained as white solid; m.p.: 64.8–66.9 °C; as a mixture of isomers (α:β = 1:8); R_f_ = 0.71 (toluene:EtOAc 2:1). ^1^H NMR (600 MHz, CDCl_3_): *δ* 5.44 (dd, 1H, *J* = 1.2 Hz, 3.6 Hz, H-4β), 5.32 (d, 1H, *J* = 1.8 Hz, H-1α), 5.20 (dd, 1H, *J* = 3.0 Hz, 10.2 Hz, H-4α), 5.15–5.14 (m, 1H, H-2α), 5.09 (t, 2H, *J* = 10.2 Hz, H-3α, H-2β), 4.99 (dd, 1H, *J* = 3.6 Hz, 10.2 Hz, H-3β), 4.69 (d, 1H, *J* = 1.2 Hz, H-1β), 4.03 (dq, 1H, *J* = 6.0 Hz, 9.6 Hz, H-5α), 3.63 (dq, 1H, *J* = 6.0 Hz, 9.6 Hz, H-5β), 2.21 (s, 3H, OAcβ), 2.17 (s, 3H, OAcα), 2.06 (s, 6H, OAcα, OAcβ), 1.99 (s, 6H, OAcα, OAcβ), 1.33 (d, 3H, *J* = 6.0 Hz, H-6β), 1.28 (d, 3H, *J* = 6.6 Hz, H-6α); ^13^C NMR (150 MHz, CDCl_3_): *δ* 170.1, 170.0, 170.0, 87.6, 70.6, 69.6, 68.8, 68.4, 21.0, 20.9, 20.8, 17.6; MS(ESI): calcd. for C_12_H_17_N_3_O_7_ ([M+H]^+^): *m*/*z* 316.1 found: *m*/*z* 315.9.

1-Azido-4-*O*-acetyl-2,3,6-trideoxy-L*-erythro*-hex-2-enopyranoside **6**

Solution of 3,4-di-*O*-acetyl-L-rhamnal (500 mg, 2.334 mmol) and iodine (47.5 mg, 0.187 mmol) in DCM (5 mL) was cooled to 0 °C while stirring. After 10 min, TMSN_3_ (460 µL, 3.501 mmol) was added dropwise at 0 °C and allowed to stir at room temperature for 8 h. After consumption of sugar substrate (TLC: toluene/EtOAc 2:1), the reaction mixture was quenched with water (2 × 10 mL) and then aqueous phase was extracted with DCM (2 × 10 mL). Combined organic extracts were dried over MgSO_4_, filtered and concentrated on a rotary evaporator. The residue was purified by column chromatography on silica gel (eluent: toluene) to afford product in the form of a colorless solidifying oil with 78% (363.6 mg) yield as a mixture of isomers (α:β = 1:1.5); R_f_ = 0.82 (toluene:EtOAc 2:1). ^1^H NMR (400 MHz, CDCl_3_): *δ* 5.99 (ddd, 1H, *J* = 2.0 Hz, 2.8 Hz, 6.0 Hz, H-3β), 5.93 (dt, 1H, *J* = 1.6 Hz, 10.4 Hz, H-3α), 5.81 (dt, 1H, *J* = 1.6 Hz, 10.4 Hz, H-2β), 5.76 (ddd, 1H, *J* = 2.0 Hz, 2.8 Hz, 10.0 Hz, H-2α), 5.63 (bs, 1H, H-1α), 5.46–5.48 (m, 1H, H-4α), 5.37 (d, *J* = 5.2 Hz, 1H, H-1β), 5.25–5.27 (m, 1H, H-4β), 4.11–4.16 (m, 1H, H-5), 3.96–4.01 (m, 1H, H-5), 2.15 (s, 3H, OAc), 2.13 (s, 3H, Oac), 1.31 (d, 3H, *J* = 6.4 Hz, H-6β), 1.29 (d, 3H, *J* = 6.0 Hz, H-6α); ^13^C NMR (100 MHz, CDCl_3_): *δ* 170.2, 170.0, 130.3, 129.4, 128.1, 126.4, 88.8, 84.6, 73.0, 72.5, 69.4, 67.1, 20.0, 20.8, 17.1, 16.9; MS(ESI): calcd. For C_8_H_11_N_3_O_3_ ([M+H]^+^): *m*/*z* 198.1, found: *m*/*z* 198.9.

#### 4.2.2. Synthesis of 3,4-Dichloro-Furan-2(5H)-One and 2H-Pyrrol-2-One Derivatives

3,4-Dichloro-5-(prop-2-yn-1-yloxy)-furan-2(5*H*)-one **7**

To a stirred solution of MCA (150 mg, 0.888 mol) dissolved in toluene (5 mL), propargyl alcohol (517 µL, 8.878 mmol) and H_2_SO_4_ (3 µL, 0.044 mmol) were added. The mixture was refluxed on magnetic stirrer for 30 min. After decay of substrate (TLC: *n*-hexane/EtOAc 1:1), K_2_CO_3_ (4.5 mg, 0.044 mmol) was added to the reaction mixture and stirred for about 30 min. Next, precipitate was filtered off, and the mixture concentrated on a rotary evaporator. The residual oil was purified by column chromatography on silica gel (toluene) to afford product as a colorless oil with 79% (145.2 mg) yield; R_f_ = 0.78 (*n*-hexane:EtOAc). ^1^H NMR (600 MHz, CDCl_3_): δ 6.06 (s, 1H, H-5), 4.51 (m, 2H, -CH_2_-), 2.64 (t, *J* = 2.4 Hz, 1H, ≡CH); ^13^C NMR (100 MHz, CDCl_3_): 163.1, 147.5, 124.7, 98.6, 77.8, 76.8, 57.4; MS(ESI): calcd. for C_7_H_4_Cl_2_O_3_ ([M+H]^+^): *m*/*z* 207.0, found: *m*/*z* 207.8.

3,4-Dichloro-1,5-dihydro-5-hydroxy-1-(prop-2-yn-1-yl)-2*H*-pyrrol-2-one **8**

To a flask with MCA (214 mg, 1.267 mmol), SOCl_2_ (1.359 g, 11.403 mmol) was added. Next, DMF (30 µL, 0.387 mmol) was added dropwise. The mixture was carried out on magnetic stirrer at 70 °C for 1 h. After consumption of substrate (TLC: toluene/EtOAc 1:1), the reaction mixture was poured into water (5 mL) and extracted with EtOAc (3 × 5 mL). Organic phase was quenched with saturated aqueous solution of NaHCO_3_ (2 × 5 mL). Next, combined organic phase was dried with MgSO_4_, filtered, and concentrated on a rotary evaporator. The resulting product was purified by column chromatography on silica gel (gradient: *n*-hexane to *n*-hexane/EtOAc 10:1, *v*/*v*) to afford 3,4,5-trichloro-furan-2(5*H*)-one in the form of a yellowish liquid with 89% yield (211.3 mg). To a stirred solution of 3,4,5-trichloro-furan-2(5*H*)-one (150 mg, 0.800 mmol), in 1,4-dioxane (5 mL) propargylamine (96.5 mg, 1.736 mmol) was added dropwise. The mixture was stirred on magnetic stirrer at room temperature for 24 h. After decay of substrate (TLC: *n*-hexane/EtOAc 1:1), the reaction mixture was poured into saturated aqueous solution of NH_4_Cl (20 mL) and extracted with EtOAc (2 × 20 mL). Combined organic phases were washed with brine (2 × 15 mL) and then dried over MgSO_4_, filtered and concentrated on a rotary evaporator. The resulting product was purified by column chromatography on silica gel (gradient from *n*-hexane to *n*-hexane/EtOAc 5:1) to afford product in the form of a yellow solid with 45% (74.2 mg) yield; m.p.: 106.8–107.8 °C; R_f_ = 0.67 (*n*-hexane:EtOAc 1:1). ^1^H NMR (600 MHz, DMSO-*d_6_*): *δ* 7.25 (d, *J* = 9.4 Hz, 1H, -OH), 5.47 (d, *J* = 9.4 Hz, 1H, H-5), 4.32 (dd, *J* = 17.9 Hz, 2.5 Hz, 1H, >N-CH_2b_-C≡CH), 3.95 (dd, *J* = 17.9 Hz, 2.5 Hz, 1H, >N-CH_2a_-C≡CH), 3.26 (t, *J* = 2.5 Hz, 1H, >N-CH_2_-C≡CH); ^13^C NMR (150 MHz, DMSO-*d_6_*): δ 161.1, 145.0, 124.4, 81.2, 78.5, 74.5, 29.1; MS(ESI): calcd. For C_7_H_5_Cl_2_NO_2_ ([M+H]^+^): *m*/*z* 206. 0, found: *m*/*z* 206.7.

3,4-Dichloro-1,5-dihydro-1-(prop-2-yn-1-yl)-5-(triisopropylsilyloxy)-2*H*-pyrrol-2-one **9**

To a stirred solution of **8** (200 mg, 0.971 mmol) in DMF (5 mL), imidazole (13 mg, 0.194 mmol) and triethylamine (270 µL, 1.942 mmol) were added. The mixture was stirred at room temperature for 15 min and triisopropylsilyl chloride (225 mg, 1.165 mmol) was added in portions, and the stirring was continued at room temperature for 24 h. After consumption of substrate (TLC: *n*-hexane/EtOAc 1:1), the reaction mixture was poured into ice-water (2 × 15 mL) and extracted with DCM (3 × 30 mL). Combined organic phases were dried over MgSO_4_, filtered and concentrated using rotary evaporator. The residual oil was purified by column chromatography on silica gel (gradient from *n*-hexane to *n*-hexane/EtOAc 3.5:1) to afford product in the form of a white semi crystal in 71% yield (249.8 mg); R_f_ = 0.64 (*n*-hexane:EtOAc 1:1). ^1^H NMR (600 MHz, DMSO-*d_6_*): *δ* 5.74 (s, 1H, H-5), 4.73 (dd, *J* = 18.0 Hz, 2.4 Hz, 1H, >N-CH_2a_-C≡CH), 3.81 (dd, *J* = 17.4 Hz, 2.4 Hz, 1H, >N-CH_2b_-C≡CH), 2.26–2.27 (m, 1H, >N-CH_2_-C≡CH), 1.24–1.29 (m, 3H, -CH(CH_3_)_2_), 1.15 (m, 3H, -CH(CH_3_)_2_), 1.14 (m, 3H, -CH(CH_3_)_2_), 1.05 (m, 3H, -CH(CH_3_)_2_); ^13^C NMR (150 MHz, DMSO-*d_6_*): *δ* 162.1, 144.2, 126.7, 81.9, 77.8, 73.1, 29.45, 18.2, 18.2, 17.9, 13.2, 12.4; MS(ESI): calcd. for C_16_H_25_Cl_2_NO_2_Si ([M+H]^+^): *m*/*z* 362.1, found: *m*/*z* 362.4.

#### 4.2.3. Synthesis of Glycoconjugates **10**–**26**

**General procedure B**

To a solution of CuSO_4_·5H_2_O (0.2 eq) in H_2_O (2.5 mL), sodium ascorbate (0.4 eq) dissolved in H_2_O (2.5 mL) was added. The resulting mixture was then added to a stirred solution of sugar azide **1**–**6** (1.0 eq) and propargyl derivative of 3,4-dichloro-furan-2(5*H*)-one **7** (1.0 eq) or 2*H*-pyrrol-2-one **8** or **9** (1.0 eq) dissolved in 10 mL of mixture of solvents THF: isopropanol (1:1, *v*/*v*). The stirring was continued at room temperature for 24 h. After consumption of sugar substrate (TLC: toluene/EtOAc 2:1), the reaction mixture was concentrated on a rotary evaporator and purified by column chromatography on silica gel to provide products **10**–**26**.

3,4-Dichloro-5-((1-(3-(1-*O*-propyl)-4-*O*-acetyl-2,3,6-trideoxy-L-*erythro*-hex-2-enopyranose)-1*H*-1,2,3-triazol-4-yl)methoxy)-furan-2(5*H*)-one **10**

Obtained according to General Procedure B starting from 1-(3-azidopropyloxy)-4-*O*-acetyl-2,3,6-trideoxy-L-*erythro*-hex-2-enopyranoside **1** (100 mg, 0.392 mmol) and 3,4-dichloro-5-(prop-2-yn-1-yloxy)-furan-2(5*H*)-one **7** (81.1 mg, 0.392 mmol), yield: 87% (157.6 mg), product was obtained in the form of yellowish oil; as a mixture of isomers (α:β = 1:6); R_f_ = 0.37 (toluene:EtOAc 2:1). ^1^H NMR (600MHz, CDCl_3_): *δ* 7.71 (s, 1H, H-5*_triazole_*), 5.94 (m, 1H, H-5*_furanone_*), 5.85–5.87 (m, 1H, H-3), 5.78–5.81 (m, 1H, H-2), 5.04–5.07 (m, 1H, H-4), 4.98–5.01 (m, 2H, -C-CH_2_-N<), 4.96 (m, 1H, H-1), 3.94 (dq, *J* = 6.0 Hz, 9.0 Hz, 1H, H-5), 3.87 (dt, *J* = 6.0 Hz, 10.2 Hz, 1H, -O-CH_2_-CH_2_-CH_2a_-N-), 3.57 (dt, *J* = 6.0 Hz, 9.6 Hz, Hz, 1H, -O-CH_2_-CH_2_-CH_2b_-N-), 3.40–3.43 (m, 2H, -O-CH_2_-CH_2_-CH_2_-N-), 2.09 (s, 3H, OAcα), 2.08 (s, 3H, OAcβ), 1.87–1.91 (m, 2H, -O-CH_2_-CH_2_-CH_2_-N-), 1.31 (d, *J* = 6.6 Hz, 3H, H-6β), 1.23 (d, *J* = 6.0 Hz, 3H, H-6α); ^13^C NMR (150MHz, CDCl_3_): *δ* 170.6, 162.3, 147.0, 140.4, 131.0, 129.9, 127.7, 125.1, 102.1, 94.6, 70.9, 65.2, 65.1, 48.6, 29.4, 21.9, 18.7, 18.1; MS(ESI): calcd. for C_18_H_21_Cl_2_N_3_O_7_ ([M+H]^+^): *m*/*z* 462.1, found: *m*/*z* 462.0.

3,4-Dichloro-*N*-((1-(3-(1-*O*-propyl)-4-*O*-acetyl-2,3,6-trideoxy-L-*erythro*-hex-2-enopyranose)-1*H*-1,2,3-triazol-4-yl)-methyl)-2*H*-pyrrol-2-one **11**

Obtained according to General Procedure B starting from 1-(3-azidopropyloxy)-4-*O*-acetyl-2,3,6-trideoxy-L-*erythro*-hex-2-enopyranoside **1** (100 mg, 0.392 mmol) and 3,4-dichloro-1,5-dihydro-5-hydroxy-1-(prop-2-yn-1-yl)-2*H*-pyrrol-2-one **8** (80.8 mg, 0.392 mmol), yield: 79% (142.8 mg), product was obtained in the form of yellowish oil as a mixture of isomers (α:β = 1:5); R_f_ = 0.18 (toluene:EtOAc 2:1). ^1^H NMR (400 MHz, CDCl_3_): *δ* 7.59 (s, 1H, H-5*_triazole_*), 6.70 (d, *J* = 2.8 Hz, 1H, H-5*_furanone_*) 5.85–5.88 (m, 1H, H-3), 5.76–5.80 (m, 1H, H-2), 5.03–5.06 (m, 1H, H-4), 4.93 (m, 1H, H-1), 4.80 (d, *J* = 15.6 Hz, 1H, =C-CH_a_-N<), 4.57 (d, *J* = 15.6 Hz, 1H, =C-CH_b_-N<), 4.46 (t, *J* = 7.2 Hz, 2H, -O-CH_2_-CH_2_-CH_2_-N-), 3.92 (dq, *J* = 6.4 Hz, 9.2 Hz, 1H, H-5), 3.76–3.82 (m, 1H, -O-CH_a_-CH_2_-CH_2_-N-), 3.43–3.49 (m, 1H, -O-CH_b_-CH_2_-CH_2_-N-), 2.19 (m, 2H, -O-CH_2_-CH_2_-CH_2_-N-), 2.10 (s, 3H, OAc), 2.08 (s, 3H, OAc), 1.30 (d, *J* = 6.4 Hz, 3H, H-6β), 1.21 (d, *J* = 6.0 Hz, 3H, H-6α); ^13^C NMR (100 MHz, CDCl_3_): *δ* 170.6, 170.2, 163.2, 145.1, 143.0, 140.9, 130.0, 127.5, 125.4, 124.5, 94.7, 80.3, 70.9, 65.2, 64.7, 47.6, 36.9, 30.5, 21.2, 18.1; MS(ESI): calcd. for C_18_H_22_Cl_2_N_4_O_6_ ([M+H]^+^): *m*/*z* 461.1, found: *m*/*z* 461.4.

3,4-Dichloro-*N*-((1-(3-(1-*O*-propyl)-4-*O*-acetyl-2,3,6-trideoxy-β-L-*erythro*-hex-2-enopyranose)-1*H*-1,2,3-triazol-4-yl)-methyl)-5-isopropylsilyloxy-2*H*-pyrrol-2-one **12**

Obtained according to General Procedure B starting from 1-(3-azidopropyloxy)-4-*O*-acetyl-2,3,6-trideoxy-β-L-*erythro*-hex-2-enopyranoside **1** (100 mg, 0.392 mmol) and 3,4-dichloro-1,5-dihydro-5-hydroxy-1-(prop-2-yn-1-yl)-5-(triisopropylsilyloxy)-2*H*-pyrrol-2-one **9** (142.0 mg, 0.392 mmol), yield: 67% (162.1 mg), product was obtained in the form of yellowish oil as an anomer β; α^23^_D_ = −22.7 (c = 0.5, CHCl_3_); R_f_ = 0.62 (*n*-hexane:EtOAc 1:1). ^1^H NMR (400 MHz, CDCl_3_): *δ* 7.55 (d, *J* = 1.6 Hz, 1H, H-5*_triazole_*), 5.86–5.88 (m, 1H, H-2), 5.76–5.81 (m, 2H, H-3, H-5*_furanone_*), 5.05 (dq, *J* = 1.6 Hz, 9.2 Hz, 1H, =C-CH_a_-N<), 4.92–4.96 (m, 2H, H-4, =C-CH_a_-N<), 4.41–4.45 (m, 3H, =C-CH_b_-N<, -O-CH_2_-CH_2_-CH_2_-N-), 3.89–3.97 (m, 1H, H-5), 3.74–3.82 (m, 1H, -O-CH_a_-CH_2_-CH_2_-N-), 3.46 (dt, *J* = 6.0 Hz, 10.4 Hz, 1H, -O-CH_b_-CH_2_-CH_2_-N-), 2.18 (dt, *J* = 6.4 Hz, 13.8 Hz, 2H, -O-CH_2_-CH_2_-CH_2_-N-), 2.10 (s, 3H, OAc), 1.25–1.34 (m, 3H, -Si(CH(CH_3_)_2_)_3_)), 1.22 (d, *J* = 6.0 Hz, 3H, H-6), 1.14 (d, *J* = 7.2 Hz, 18H, -Si(CH(CH_3_)_2_)_3_); ^13^C NMR (100 MHz, CDCl_3_): *δ* 170.6, 162.9, 144.4, 142.9, 129.9, 127.5, 126.5, 123.3, 94.5, 82.7, 70.8, 65.1, 64.6, 47.1, 35.3, 30.4, 21.1, 18.0, 18.0, 13.1; MS(ESI): calcd. for C_27_H_42_Cl_2_N_4_O_6_Si ([M+H]^+^): *m*/*z* 617.2, found: *m*/*z* 617.5.

3,4-Dichloro-5-((1-(3-(1-*O*-propyl)-2,3,4-tri-*O*-acetyl-β-L-rhamnopyranose)-1*H*-1,2,3-triazol-4-yl)methoxy)-furan-2(5*H*)-one **13**

Obtained according to General Procedure B starting from 1-(3-azidopropyloxy)-2,3,4-tri-*O*-acetyl-β-L-rhamnopyranoside **2** (100 mg, 0.268 mmol) and 3,4-dichloro-5-(prop-2-yn-1-yloxy)-furan-2(5*H*)-one **7** (55.5 mg, 0.268 mmol), yield: 92% (143.0 mg), product was obtained in the form of colorless oil as an anomer β; α^22^_D_ = 34.7 (c = 0.5, CHCl_3_); R_f_ = 0.24 (toluene:EtOAc 2:1). ^1^H NMR (600 MHz, CDCl_3_): *δ* 7.71 (s, 1H, H-5*_triazole_*), 6.01 (d, *J* = 1.8 Hz, 1H, H-5*_furanone_*), 5.22–5.25 (m, 1H, H-4), 5.19–5.21 (m, 1H, H-2), 5.07 (t, 1H, *J* = 10.2 Hz, 1H, H-3), 4.99–5.03 (m, 2H, -C-CH_2_-N<), 4.69 (m, 1H, H-1), 4.50–4.57 (m, 2H, -O-CH_2_-CH_2_-CH_2_-N-), 3.81–3.86 (m, 1H, H-5), 3.75–3.78 (m, 1H, -O-CH_b_-CH_2_-CH_2_-N-), 3.38–3.42 (m, 1H, -O-CH_a_-CH_2_-CH_2_-N-), 2.22–2.29 (m, 2H, -O-CH_2_-CH_2_-CH_2_-N-), 2.15 (s, 3H, OAc), 2.06 (s, 3H, OAc), 2.00 (s, 3H, OAc), 1.22 (d, *J* = 6.6 Hz, 3H, H-6); ^13^C NMR (150 MHz, CDCl_3_): *δ* 170.4, 170.3, 170.1, 162.2, 146.8, 139.9, 131.3, 124.1, 100.2, 97.7, 71.0, 69.8, 69.2, 66.9, 64.5, 63.2, 47.7, 29.9, 21.0, 21.0, 20.9, 17.5; MS(ESI): calcd. for C_22_H_27_Cl_2_N_3_O_11_ ([M+H]^+^): *m*/*z* 580.1, found: *m*/*z* 580.4.

3,4-Dichloro-*N*-((1-(3-(1-*O*-propyl)-2,3,4-tri-*O*-acetyl-β-L-rhamnopyranose)-1*H*-1,2,3-triazol-4-yl)-methyl)-2*H*-pyrrol-2-one **14**

Obtained according to General Procedure B starting from 1-(3-azidopropyloxy)-2,3,4-tri-*O*-acetyl-β-L-rhamnopyranoside **2** (100 mg, 0.268 mmol) and 3,4-dichloro-1,5-dihydro-5-hydroxy-1-(prop-2-yn-1-yl)-2*H*-pyrrol-2-one **8** (55.2 mg, 0.268 mmol), yield: 83% (128.8 mg), product was obtained in the form of yellowish oil; an anomer β; α^25^_D_ = −26.9 (c = 1.0, CHCl_3_); R_f_ = 0.18 (toluene:EtOAc 2:1). ^1^H NMR (600 MHz, CDCl_3_): *δ* 7.70 (d, *J* = 3.6 Hz, 1H, H-5*_triazole_*), 5.53 (d, *J* = 4.2 Hz, 1H, H-5*_furanone_*), 5.12–5.19 (m, 2H, H-1, H-3), 5.04 (dt, *J* = 2.4 Hz, 9.6 Hz, 1H, H-2), 4.81 (dd, *J* = 8.4 Hz, 15.6 Hz, 1H, =C-CH_a_-N<), 4.66–4.70 (m, 2H, H-4, =C-CH_b_-N<), 4.42–4.50 (m, 2H, -O-CH_2_-CH_2_-CH_2_-N-), 3.78–3.83 (m, 1H, -O-CH_a_-CH_2_-CH_2_-N-), 3.72–3.76 (m, 1H, H-5), 3.38–3.42 (m, 1H, -O-CH_b_-CH_2_-CH_2_-N-), 2.17–2.26 (m, 2H, -O-CH_2_-CH_2_-CH_2_-N-), 2.13 (s, 3H, OAc), 2.05 (s, 3H, OAc), 1.98 (s, 3H, OAc), 1.19 (dd, *J* = 1.8 Hz, 6.0 Hz, 3H, H-6); ^13^C NMR (150 MHz, CDCl_3_): *δ* 170.5, 170.4, 170.1, 162.8, 144.2, 143.1, 129.1, 123.5, 97.7, 82.3, 70.9, 69.8, 69.3, 66.8, 64.7, 47.9, 35.7, 29.8, 21.0, 20.9, 20.9, 17.5; MS(ESI): calcd. for C_22_H_28_Cl_2_N_4_O_10_ ([M+H]^+^): *m*/*z* 579.1, found: *m*/*z* 580.0.

3,4-Dichloro-*N*-((1-(3-(1-*O*-propyl)-2,3,4-tri-*O*-acetyl-β-L-rhamnopyranose)-1*H*-1,2,3-triazol-4-yl)-methyl)-5-triisopropylsilyloxy-2*H*-pyrrol-2-one **15**

Obtained according to General Procedure B starting from 1-(3-azidopropyloxy)-2,3,4-tri-*O*-acetyl-β-L-rhamnopyranoside **2** (100 mg, 0.268 mmol) and 3,4-dichloro-1,5-dihydro-5-hydroxy-1-(prop-2-yn-1-yl)-5-(triisopropylsilyl)-2*H*-pyrrol-2-one **9** (97.1 mg, 0.268 mmol), yield: 75% (147.8 mg), product was obtained in the form of yellowish oil as an anomer β; α^25^_D_ = −9.3 (c = 1.0, CHCl_3_); R_f_ = 0.4 (toluene:EtOAc 2:1). ^1^H NMR (600 MHz, CDCl_3_): *δ* 7.56 (d, *J* = 4.8 Hz, 1H, H-5*_triazole_*), 5.79 (d, *J* = 13.2 Hz, 1H, H-5*_furanone_*), 5.21–5.26 (m, 2H, H-1, H-3), 5.06 (t, *J* = 10.2 Hz, 1H, H-2), 4.95 (dd, *J* = 3.6 Hz, 15.6 Hz, 1H, =C-CH_a_-N<), 4.69–4.70 (m, 1H, H-4), 4.37–4.48 (m, 3H, =C-CH_b_-N<, -O-CH_2_-CH_2_-CH_2_-N-), 3.81–3.88 (m, 1H, H-5), 3.69–3.74 (m, 1H, -O-CH_a_-CH_2_-CH_2_-N-), 3.38–3.42 (m, 1H, -O-CH_b_-CH_2_-CH_2_-N-), 2.16–2.25 (m, 2H, -O-CH_2_-CH_2_-CH_2_-N-), 2.14 (s, 3H, OAc), 2.05 (s, 3H, OAc), 1.99 (s, 3H, OAc), 1.26–1.31 (m, 3H, -Si(CH(CH_3_)_2_)_3_)), 1.21 (d, *J* = 6.6 Hz, 3H, H-6), 1.12 (dd, *J* = 2.4 Hz, 7.2 Hz, 18H, -Si(CH(CH_3_)_2_)_3_); ^13^C NMR (150 MHz, CDCl_3_): *δ* 170.3, 170.2, 170.1, 162.9, 144.4, 143.3, 139.6, 123.0, 97.7, 82.7, 71.0, 69.8, 69.2, 66.8, 64.4, 47.3, 35.3, 30.0, 21.0, 20.9, 20.9, 18.2, 18.2, 17.5, 13.2; MS(ESI): calcd. for C_31_H_48_Cl_2_N_4_O_10_Si ([M+Na]^+^): *m*/*z* 757.2, found: *m*/*z* 757.5.

3,4-Dichloro-5-((1-(2,3,4,6-tetra-*O*-acetyl-β-D-glucopyranose)-1*H*-1,2,3-triazol-4-yl)methoxy)-furan-2(5H)-one **16**

Obtained according to General Procedure B starting from 1-azido-2,3,4,6-tetra-*O*-acetyl-β-D-glucopyranoside **3** (100 mg, 0.268 mmol) and 3,4-dichloro-5-(prop-2-yn-1-yloxy)-furan-2(5*H*)-one **7** (55.5 mg, 0.268 mmol), yield: 85% (132.1 mg), product was obtained in the form of yellowish, solidifying oil as an anomer β; α^24^_D_ = −8.1 (c = 1.0, CHCl_3_); R_f_ = 0.33 (toluene:EtOAc 2:1). ^1^H NMR (400 MHz, CDCl_3_): *δ* 7.88 (d, *J* = 5.2 Hz, 1H, H-5*_triazole_*), 5.95 (d, *J* = 6.4 Hz, 1H, H-5*_furanone_*), 5.89 (d, *J* = 9.2 Hz, 1H, H-1), 5.45–5.36 (m, 2H, -CH_2_-), 5.25 (t, *J* = 9.6 Hz, 1H, H-2), 5.04–4.95 (m, 2H, H-3, H-4), 4.32 (dd, *J* = 5.2 Hz, 12.8 Hz, 1H, H-6a), 4.16 (dd, *J* = 2.0 Hz, 12.8 Hz, 1H, H-6b), 4.02 (ddd, *J* = 2.4 Hz, 5.2 Hz, 10.4 Hz, 1H, H-5), 2.09 (s, 3H, OAc), 2.08 (s, 3H, OAc), 2.03 (s, 3H, OAc), 1.89 (s, 3H, OAc); ^13^C NMR (100 MHz, CDCl_3_): *δ* 170.6, 170.0, 169.5, 169.1, 163.1, 147.5, 138.0, 122.2, 99.7, 86.1, 86.1, 75.4, 72.6, 70.6, 67.8, 62.8, 61.6, 20.8, 20.7, 20.6, 20.3; MS(ESI): calcd. for C_21_H_23_Cl_2_N_3_O_12_ ([M+H]^+^): *m*/*z* 580.1, found: *m*/*z* 581.0.

3,4-Dichloro-*N*-((1-(2,3,4,6-tetra-*O*-acetyl-β-D-glucopyranose)-1*H*-1,2,3-triazol-4-yl)-methyl)-2*H*-pyrrol-2-one **17**

Obtained according to General Procedure B starting from 1-azido-2,3,4,6-tetra-*O*-acetyl-β-D-glucopyranoside **3** (100 mg, 0.268 mmol) and 3,4-dichloro-1,5-dihydro-5-hydroxy-1-(prop-2-yn-1-yl)-2*H*-pyrrol-2-one **8** (55.2 mg, 0.268 mmol), yield: 75% (116.4 mg), product was obtained in the form of yellowish oil as an anomer β; α^24^_D_ = −26.1 (c = 1.0, CHCl_3_); R_f_ = 0.27 (toluene:EtOAc 2:1). ^1^H NMR (600 MHz, CDCl_3_): *δ* 7.96 (bs, 1H, H-5*_triazole_*), 5.84 (m, 1H, H-5*_furanone_*), 5.41–5.45 (m, 2H, =C-CH_2_-N<), 5.29–5.35 (m, 1H, H-1), 5.24 (t, *J* = 9.6 Hz, 1H, H-2), 4.72–4.85 (m, 2H, H-3, H-4), 4.30–4.33 (m, 1H, H-6_a_), 4.16 (d, *J* = 12.6 Hz, 1H, H-5), 4.01–4.04 (m, 1H, H-6_b_), 2.09 (s, 3H, OAc), 2.07 (s, 3H, OAc), 2.02 (s, 3H, OAc), 2.01 (s, 3H, OAc); ^13^C NMR (100 MHz, CDCl_3_): *δ* 170.7, 170.7, 169.5, 169.2, 162.8, 144.2, 144.0, 129.2, 126.5, 86.3, 82.4, 75.5, 72.3, 70.9, 67.7, 61.6, 35.5, 20.8, 20.7, 20.6, 20.4; MS(ESI): calcd. for C_21_H_24_Cl_2_N_4_O_11_ ([M+H]^+^): *m*/*z* 579.1, found: *m*/*z* 579.4.

3,4-Dichloro-*N*-((1-(2,3,4,6-tetra-*O*-acetyl-β-D-glucopyranose)-1*H*-1,2,3-triazol-4-yl)-methyl)-5-triisopropylsilyloxy-2*H*-pyrrol-2-one **18**

Obtained according to General Procedure B starting from 1-azido-2,3,4,6-tetra-*O*-acetyl-β-D-glucopyranoside **3** (100 mg, 0.268 mmol) and 3,4-dichloro-1,5-dihydro-5-hydroxy-1-(prop-2-yn-1-yl)-5-(triisopropylsilyl)-2*H*-pyrrol-2-one **9** (97.1 mg, 0.268 mmol), yield: 64% (129.9 mg product was obtained in the form of colorless oil as an anomer β; α^22^_D_ = −13.3 (c = 1.0, CHCl_3_). ^1^H NMR (600 MHz, CDCl_3_): *δ* 7.74 (bs, 1H, H-5*_triazole_*), 5.87 (m, 1H, H-5*_furanone_*), 5.42–5.45 (m, 2H, =C-CH_2_-N<), 5.27–5.40 (m, 1H, H-1), 5.17–5.19 (m, 1H, H-2), 4.73–4.82 (m, 2H, H-3, H-4), 4.29–4.34 (m, 1H, H-6_a_), 4.15 (d, *J* = 12.6 Hz, 1H, H-5), 3.99–4.05 (m, 1H, H-6_b_), 2.11 (s, 3H, OAc), 2.07 (s, 3H, OAc), 2.05 (s, 3H, OAc), 2.02 (s, 3H, OAc), 1.26–1.31 (m, 3H, -Si(CH(CH_3_)_2_)_3_)); ^13^C NMR (100 MHz, CDCl_3_): *δ* 170.6, 170.5, 169.7, 169.2, 162.9, 145.2, 144.6, 129.0, 128.1, 86.9, 82.7, 75.7, 71.8, 71.2, 68.1, 61.2, 35.7, 21.1, 20.9, 20.7, 20.6, 20.5, 20.2, 18.9, 18.1; MS(ESI): calcd. for C_30_H_44_Cl_2_N_4_O_11_Si ([M+H]^+^): *m*/*z* 735.2, found: *m*/*z* 735.6.

3,4-Dichloro-5-((1-(2,3,4-tri-*O*-acetyl-β-L-fucopyranose)-1*H*-1,2,3-triazol-4-yl)methoxy)-furan-2(5*H*)-one **19**

Obtained according to General Procedure B starting from 1-azido-2,3,4-tri-*O*-acetyl-β-L-fucopyranoside **4** (100 mg, 0.317 mmol) and 3,4-dichloro-5-(prop-2-yn-1-yloxy)-furan-2(5*H*)-one **7** (65.6 mg, 0.317 mmol), yield: 78% (129.2 mg), product was obtained in the form of colorless, solidifying oil as an anomer β. A^24^_D_ = −35.5 (c = 1.0, CHCl_3_); R_f_ = 0.42 (toluene:EtOAc 2:1). ^1^H NMR (400 MHz, CDCl_3_): *δ* 7.83 (d, *J* = 3.2 Hz, 1H, H-5*_triazole_*), 5.95 (d, *J* = 4.8 Hz, 1H, H-5*_furanone_*), 5.82 (d, *J* = 9.2 Hz, 1H, H-1), 5.40–5.51 (m, 2H, -CH_2_-), 5.23–5.27 (m, 1H, H-2) 4.98–5.01 (m, 2H, H-3, H-4), 4.13 (dq, *J* = 6.0 Hz, 6.8 Hz, 1H, H-5), 2.24 (s, 3H, Oac), 2.01 (s, 3H, Oac), 1.90 (s, 3H, Oac), 1.28 (d, *J* = 6.4 Hz, 3H, H-6); ^13^C NMR (100 MHz, CDCl_3_): *δ* 170.5, 170.4, 170.0, 163.2, 143.3, 129.7, 122.4, 99.5, 86.7, 73.1, 71.1, 69.9, 68.4, 68.3, 62.8, 20.8, 20.7, 20.4, 16.2; MS(ESI): calcd. For C_19_H_21_Cl_2_N_3_O_10_ ([M+Na]^+^): *m*/*z* 544.0496, found: *m*/*z* 544.4.

3,4-Dichloro-*N*-((1-(2,3,4-tri-*O*-acetyl-β-L-fucopyranose)-1*H*-1,2,3-triazol-4-yl)-methyl)-2*H*-pyrrol-2-one **20**

Obtained according to General Procedure B starting from 1-azido-2,3,4-tri-*O*-acetyl-β-L-fucopyranoside **4** (100 mg, 0.317 mmol) and 3,4-dichloro-1,5-dihydro-5-hydroxy-1-(prop-2-yn-1-yl)-2*H*-pyrrol-2-one **8** (65.3 mg, 0.317 mmol), yield: 68% (112.4 mg), product was obtained in the form of yellowish oil as an anomer β; α^23^_D_ = 24.9 (c = 1.0, CHCl_3_); R_f_ = 0.2 (toluene:EtOAc 2:1). ^1^H NMR (600 MHz, CDCl_3_): *δ* 7.86 (bs, 1H, H-5*_triazole_*), 5.90 (dd, *J* = 1.8 Hz, 6.6 Hz, 1H, H-5*_furanone_*), 5.80–5.81 (m, 1H, H-2), 5.61–5.75 (m, 2H, H-1, H-3), 5.14–5.18 (m, 1H, =C-CH_2a_-N<), 4.85–4.95 (m, 1H, =C-CH_2b_-N<), 4,70–4.76 (m, 1H, H-3), 3.84 (dq, *J* = 6.0 Hz, 9.6 Hz, 1H, H-5), 2.16 (s, 3H, OAc), 2.15 (s, 3H, OAc), 2.06 (s, 3H, OAc), 1.22 (d, *J* = 6.0 Hz, 3H, H-6);

^13^C NMR (100 MHz, CDCl_3_): *δ* 170.5, 170.0, 162.9, 144.3, 127.6, 126.5, 124.3, 84.3, 70.3, 69.3, 69.1, 68.2, 35.1, 20.9, 20.9, 20.7, 20.7, 17.4; MS(ESI): calcd. for C_19_H_22_Cl_2_N_4_O_9_ ([M+H]^+^): *m*/*z* 521.1, found: *m*/*z* 521.2.

3,4-Dichloro-*N*-((1-(2,3,4-tri-*O*-acetyl-β-L-fucopyranose)-1*H*-1,2,3-triazol-4-yl)-methyl)-5-triisopropylsilyloxy-2*H*-pyrrol-2-one **21**

Obtained according to General Procedure B starting from 1-azido-2,3,4-tri-*O*-acetyl-β-L-fucopyranoside **4** (100 mg, 0.317 mmol) and 3,4-dichloro-1,5-dihydro-5-hydroxy-1-(prop-2-yn-1-yl)-5-(triisopropylsilyl)-2*H*-pyrrol-2-one **9** (114.9 mg, 0.317 mmol), yield: 46% (98.9 mg), product was obtained in the form of yellowish oil as an anomer β; α^22^_D_ = −38.7 (c = 1.0, CHCl_3_). ^1^H NMR (600 MHz, CDCl_3_): *δ* 7.76 (d, *J* = 4.2 Hz, 1H, H-5*_triazole_*), 5.89 (s, 1H, =C-CH_a_-N<), 5.78–5.85 (m, 1H, H-3), 5.75 (s, 1H, =C-CH_b_-N<), 5.15 (t, *J* = 9.0 Hz, 1H, H-4), 4.96 (dd, *J* = 3.0 Hz, 15.6 Hz, 1H, H-2), 4.46 (d, *J* = 15.6 Hz, 1H, H-1), 3.67–3.75 (m, 1H, H-5), 2.17 (s, 3H, OAc), 2.05 (s, 3H, OAc), 2.02 (s, 3H, OAc), 1.27–1.32 (m, 3H, -Si(CH(CH_3_)_2_)_3_)), 1.24 (d, *J* = 6.6, Hz, 3H, H-6α), 1.22 (d, *J* = 6.0 Hz, 3H, H-6β), 1.16–1.22 (m, 18H, -Si(CH(CH_3_)_2_)_3_); ^13^C NMR (150 MHz, CDCl_3_): *δ* 170.0, 169.8, 169.6, 162.9, 144.5, 143.8, 128.3, 122.9, 83.9, 82.6, 70.9, 70.2, 68.9, 35.1, 20.9, 20.7, 18.2, 17.8, 17.4, 17.1, 13.2, 13.2; MS(ESI): calcd. for C_28_H_42_Cl_2_N_4_O_9_Si ([M+H]^+^): *m*/*z* 677.2, found: *m*/*z* 677.6.

3,4-Dichloro-5-((1-(2,3,4-tri-*O*-acetyl-β-L-rhamnopyranose)-1*H*-1,2,3-triazol-4-yl) methoxy)-furan-2(5*H*)-one **22**

Obtained according to General Procedure B starting from 1-azido-2,3,4-tri-*O*-acetyl-L-rhamnopyranoside **5** (100 mg, 0.317 mmol) and 3,4-dichloro-5-(prop-2-yn-1-yloxy)-furan-2(5*H*)-one **7** (65.6 mg, 0.317 mmol), yield: 79% (130.9 mg), product was obtained in the form of yellowish oil as an anomer β; α^24^_D_ = −35.5 (c = 1.0, CHCl_3_); R_f_ = 0.42 (toluene:EtOAc 2:1). ^1^H NMR (400 MHz, CDCl_3_): *δ* 7.95 (d, *J* = 3.2 Hz, 1H, H-5*_triazole_*), 5.95 (d, *J* = 4.8 Hz, 1H, H-5*_furanone_*), 5.82 (d, *J* = 9.2 Hz, 1H, H-1), 5.51–5.40 (m, 2H, H-2, H-4), 5.26–5.27 (m, 1H, H-3), 4.95–5.04 (m, 2H, -CH_2_-), 4.13 (q, *J* = 6.8 Hz, 1H, H-5), 2.24 (s, 3H, OAc), 2.01 (s, 3H, OAc), 1.90 (s, 3H, OAc), 1.28 (d, *J* = 6.4 Hz, 3H, H-6); ^13^C NMR (100MHz, CDCl_3_): 170.4, 170.0, 169.5, 163.2, 147.6, 142.8, 125.4, 122.4, 99.7, 86.6, 73.0, 71.1, 70.0, 68.3, 62.8, 20.8, 20.7, 20.4, 16.2; MS(ESI): calcd. for C_19_H_21_Cl_2_N_3_O_10_ ([M+H]^+^): *m*/*z* 522.1, found: *m*/*z* 522.9.

3,4-Dichloro-*N*-((1-(2,3,4-tri-*O*-acetyl-L-rhamnopyranose)-1*H*-1,2,3-triazol-4-yl)-methyl)-2*H*-pyrrol-2-one **23**

Obtained according to General Procedure B starting from 1-azido-2,3,4-tri-*O*-acetyl-L-rhamnopyranoside **5** (100 mg, 0.317 mmol) and 3,4-dichloro-1,5-dihydro-5-hydroxy-1-(prop-2-yn-1-yl)-2*H*-pyrrol-2-one **8** (63.3 mg, 0.317 mmol), yield: 71% (117.4 mg), product was obtained in the form of yellowish oil; as a mixture of isomers (α:β = 1:3); R_f_ = 0.33 (toluene:EtOAc 2:1). ^1^H NMR (400MHz, CDCl_3_): δ 7.86 (d, *J* = 4.8 Hz, 1H, H-5*_triazole-_*_α_), 7.79 (d, *J* = 1.2 Hz, 1H, H-5*_triazole-__β_*), 6.71 (d, *J* = 8.0 Hz, 1H, H-5*_furanone-β_*), 6.67 (s, 1H, H-5*_furanone-α_*), 6.13 (dd, *J* = 2.4 Hz, 4.4 Hz, 1H, H-1β), 5.74–5.79 (d, *J* = 9.6 Hz, 1H, H-1α), 5.15–5.23 (m, 2H, H-2α, H-2β), 4.77–4.87 (m, 2H, H-4α, H-β), 4.61–4.65 (m, 2H, H-3α, H-β), 4.49–4.89 (m, 2H, -CH_2_-), 4.34 (q, *J* = 4.8 Hz, 10.8 Hz, 1H, H-5β), 4.10 (q, *J* = 6.0 Hz, 13.2 Hz, 1H, H-5α), 2.25 (s, 3H, OAcα), 2.18 (s, 3H, OAcα), 2.17 (s, 3H, OAcα), 2.14 (s, 3H, OAcβ), 2.10 (s, 3H, OAcβ), 2.09 (s, 3H, -OHα), 1.99 (s, 3H, OAcβ), 1.30 (d, *J* = 6.0 Hz, 3H, H-6β), 1.25 (d, *J* = 6.4 Hz, 3H, H-6α); ^13^C NMR (100 MHz, CDCl_3_): *δ* 170.5, 170.1, 170.0, 169.5, 169.3, 163.1, 143.1, 141.0, 128.0, 122.3, 121.6, 92.0, 86.5, 80.2, 79.9, 73.0, 72.9, 71.2, 71.1, 70.0, 68.3, 36.7, 36.6, 21.2, 20.8, 20.9, 20.6, 20.4, 20.3, 16.4, 16.2; MS(ESI): calcd. for C_19_H_22_Cl_2_N_4_O_9_ ([M+H]^+^): *m*/*z* 521.1, found: *m*/*z* 521.9.

3,4-Dichloro-*N*-((1-(2,3,4-tri-*O*-acetyl-L-rhamnopyranose)-1*H*-1,2,3-triazol-4-yl)-methyl)-5-triisopropylsilyloxy-2*H*-pyrrol-2-one **24**

Obtained according to General Procedure B starting from 1-azido-2,3,4-tri-*O*-acetyl-L-rhamnopyranoside **5** (100 mg, 0.317 mmol) and 3,4-dichloro-1,5-dihydro-5-hydroxy-1-(prop-2-yn-1-yl)-5-(triisopropylsilyl)-2*H*-pyrrol-2-one **9** (114.9 mg, 0.317 mmol), yield: 52% (111.8 mg), product was obtained in the form of yellowish oil; as a mixture of isomers (α:β = 1:1); R_f_ = 0.4 (toluene:EtOAc 2:1). ^1^H NMR (400MHz, CDCl_3_): δ 7.86 (d, *J* = 4.8 Hz, 1H, H-5*_triazole-α_*), 7.79 (d, *J* = 1.2 Hz, 1H, H-5*_triazole-β_*), 6.71 (d, *J* = 8.0 Hz, 1H, H-5*_furanone-β_*), 6.67 (s, 1H, H-5*_furanone-α_*), 6.13 (dd, *J* = 2.4 Hz, 4.4 Hz, 1H, H-1β), 5.74–5.79 (d, *J* = 9.6 Hz, 1H, H-1α), 5.15–5.23 (m, 2H, H-2α, H-2β), 4.77–4.87 (m, 2H, H-4α, H-β), 4.61–4.65 (m, 2H, H-3α, H-β), 4.49–4.89 (m, 2H, -CH_2_-), 4.34 (q, *J* = 4.8 Hz, 10.8 Hz, 1H, H-5β), 4.10 (q, *J* = 6.0 Hz, 13.2 Hz, 1H, H-5α), 2.25 (s, 3H, OAcα), 2.18 (s, 3H, Oacα), 2.17 (s, 3H, Oacα), 2.14 (s, 3H, Oacβ), 2.10 (s, 3H, Oacβ), 2.09 (s, 3H, -Ohα), 1.99 (s, 3H, Oacβ), 1.30 (d, *J* = 6.0 Hz, 3H, H-6β), 1.25 (d, *J* = 6.4 Hz, 3H, H-6α), 1.13–1.16 (m, 18H, -Si(CH(CH_3_)_2_)_3_); ^13^C NMR (100 MHz, CDCl_3_): *δ* 170.5, 170.1, 170.0, 169.5, 169.3, 163.1, 143.1, 141.0, 128.0, 122.3, 121.6, 92.0, 86.5, 80.2, 79.9, 73.0, 72.9, 71.2, 71.1, 70.0, 68.3, 36.7, 36.6, 21.2, 20.8, 20.9, 20.6, 20.4, 20.3, 16.4, 16.2; MS(ESI): calcd. For C_28_H_42_Cl_2_N_4_O_9_Si ([M+H]^+^): *m*/*z* 677.2, found: *m*/*z* 677.6.

3,4-Dichloro-*N*-((1-(4-*O*-acetyl-2,3,6-trideoxy-β-L-*erythro*-hex-2-enopyranose)-1*H*-1,2,3-triazol-4-yl)-methyl)-2*H*-pyrrol-2-one **25**

Obtained according to General Procedure B starting from 1-azido-4-*O*-acetyl-2,3,6-trideoxy-L-*erythro*-hex-2-enopyranoside **6** (100 mg, 0.507 mmol) and 3,4-dichloro-1,5-dihydro-5-hydroxy-1-(prop-2-yn-1-yl)-2*H*-pyrrol-2-one **8** (104.5 mg, 0.507 mmol), yield: 63% (128.8 mg), product was obtained in the form of yellowish oil as an anomer β; α^23^_D_ = −48.0 (c = 0.25, CHCl_3_); R_f_ = 0.31 (toluene:EtOAc 2:1). ^1^H NMR (600 MHz, CDCl_3_): *δ* 7.74 (s, 1H, H-5*_triazole_*), 6.60 (dd, *J* = 2.4 Hz, 6.0 Hz, 1H, H-5*_furanone_*) 5.46–5.44 (m, 2H, H-3, H-2), 5.30–5.33 (m, 1H, H-1), 5.13–5.16 (m, 1H, H-4), 4.58–4.68 (m, 2H, -CH_2_-), 4.13–4.18 (m, 1H, H-5), 2.11 (s, 3H, OAc), 1.30 (d, *J* = 6.6 Hz, 3H, H-6); ^13^C NMR (150 MHz, CDCl_3_): *δ* 169.9, 162.8, 148.7, 144.3, 137.9, 129.1, 128.2, 126.2, 125.3, 94.5, 81.9, 71.0, 67.5, 35.1, 20.6, 17.0; MS(ESI): calcd. for C_15_H_16_Cl_2_N_4_O_5_ ([M+Na]^+^): *m*/*z* 425.0 found: *m*/*z* 425.4.

3,4-Dichloro-*N*-((1-(4-*O*-acetyl-2,3,6-trideoxy-β-L-*erythro*-hex-2-enopyranose)-1*H*-1,2,3-triazol-4-yl)-methyl)-5-triisopropylsilyloxy-2*H*-pyrrol-2-one **26**

Obtained according to General Procedure B starting from 1-azido-4-*O*-acetyl-2,3,6-trideoxy-β-L-*erythro*-hex-2-enopyranoside **6** (100 mg, 0.507 mmol) and 3,4-dichloro-1,5-dihydro-5-hydroxy-1-(prop-2-yn-1-yl)-5-(triisopropylsilyl)-2*H*-pyrrol-2-one **9** (183.7 mg, 0.507 mmol), yield: 47% (133.4 mg), product was obtained in the form of yellowish oil as an anomer β; α^23^_D_ = −50.7 (c = 0.25, CHCl_3_); R_f_ = 0.64 (toluene:EtOAc 2:1). ^1^H NMR (600 MHz, CDCl_3_): *δ* 7.55 (d, *J* = 1.6 Hz, 1H, H-5*_triazole_*), 5.86–5.88 (m, 1H, H-2), 5.76–5.81 (m, 2H, H-3, H-5*_furanone_*), 5.05 (dq, *J* = 1.6 Hz, 9.2 Hz, 1H, =C-CH_a_-N<), 4.92–4.96 (m, 2H, H-4, =C-CH_a_-N<), 4.41–3.89–3.97 (m, 1H, H-5), 2.10 (s, 3H, OAc), 1.25–1.34 (m, 3H, -Si(CH(CH_3_)_2_)_3_)), 1.22 (d, *J* = 6.0 Hz, 3H, H-6), 1.14 (d, *J* = 7.2 Hz, 18H, -Si(CH(CH_3_)_2_)_3_); ^13^C NMR (150 MHz, CDCl_3_): *δ* 169.9, 162.7, 147.4, 142.6, 129.0, 128.2, 126.6, 122.9, 94.8, 82.7, 72.8, 67.4, 35.4, 21.5, 21.0, 20.6, 18.1, 17.1, 13.1; MS(ESI): calcd. for C_24_H_36_Cl_2_N_4_O_5_Si ([M+Na]^+^): *m*/*z* 581.2, found: *m*/*z* 582.1.

### 4.3. Biological Evaluation

#### 4.3.1. Cells

For cytological studies, human cancer cell lines were selected, with special emphasis on the colorectal—HCT116 and breast—MCF-7 cancer cell lines (both obtained from the ATTC collection; Manassas, VA, USA). The cells were harvested under the standard conditions in an incubator (5% CO_2_, 37 °C and 60% humidity; Panasonic model MCO-19 AIC). Cells were cultured in T75 mL sterile bottles (Sarstedt, Nümbrecht, Germany), using DMEM-F12 medium (Merck, Darmstadt, Germany) supplemented with 10% of Fetal Bovine Serum (EURx, Gdańsk, Poland). During the experimental procedures, the 60–80% confluence of each cell line was used, with the maximum number of passages not exceeding 30. For adherent cell passage, trypsin enzyme working solution prepared in sodium phosphate buffered saline (PBS, pH = 7.4; PAN-Biotech Gmbh, Aidenbach, Germany) was used. The tested compounds were prepared as 1 mM stocks in 100% DMSO (Merck, Darmstadt, Germany) and stored at −20 °C. Before application in cytological studies, a working solution of each compound was prepared directly in complete DMEM-F12 medium.

#### 4.3.2. MTT Cytotoxicity Assay

Collected after trypsinization, HCT116 and MCF-7 cells were counted under a Bürker chamber, and 2000 or 5000 cells were seeded, respectively, on each well of a sterile 96-well format plate (Sarstedt, Nümbrecht, Germany), in 100 µL of complete DMEM-F12 medium. For 24 h before compound addition, the cells were monitored directly on plates, and only well-attached cells were used for treatments with background controls (DMSO with a final maximum concentration of 1%) and untreated controls. Different proliferation rate of both cell lines discriminates the number of cells seeded into wells, where doubling time for HCT116 cells is 14–16 h, and for MCF-7 cells it is about 24–26 h (in-house experimental observations); such a procedure allowed for prolonged, up to 72 h lifetime observations, eliminating the contact inhibition and false cytostatic effects in vitro. To treated wells, 100 µL of tested compounds in fresh medium were added at a concentration range: 100, 50, 25, 12.5, 6.25, 3.13 µM (0 µM for control, untreated cells in DMEM-F12 without addition of tested compounds). After 72 h of incubation, the viability assay was performed according to the producer protocol (Promega), where the medium over the compounds was removed and cells were washed with PBS solution. A yellow tetrazolium salt (3-(4,5-dimethylthiazol-2-yl)-2,5-diphenyltetrazolium bromide) solution (0.5 mg/mL) in serum and phenol red free DMEM (Merck, Darmstadt, Germany) in volume of 50 µL was added to each well on plates for 2–3 h in dark, 37 °C incubations. The purple formazan crystals were produced only by the viable cells, containing NAD(P)H-dependent oxidoreductase enzymes that reduce MTT. The crystals were dissolved in acidic (0.04 M HCl) 2-propanolol solution (Merck, Darmstadt, Germany) after addition of 75 µL of dissolving mixture to each well on plates, for 5–10 min in darkness, at room temperature (r.t.) incubation. The absorption at 570 nm was measured directly using multiplate reader (Epoch, BioTek, Janki, Poland). Each time the biological experiments were repeated three times.

#### 4.3.3. Cell Cycle and Cytometry Analysis of Apoptosis

For apoptotic dead cell and cell cycle estimation, cell cultures were plated in 6-well plates at a confluence of 3 × 10^5^ cells in 2 mL of complete fresh DMEM-F12 medium. After 24 h, the medium was replaced with prepared sample solutions, at doses of calculated from MTT assay IC_50_ values, respectively, for further long-term 72 h incubations. After incubation, the collected cells were centrifuged, and the pellet was dissolved in 250 μL of hypotonic buffer (hypotonic buffer comprised: PI 100 μg/mL in PBS (BD Biosciences, San Jose, CA, USA); 5 mg/L of citric acid; 1:9 Triton-X solution; RNase 100 μg/mL in PBS (Merck, Darmstadt, Germany)). The samples were incubated for 15 min at room temperature and in darkness. The cellular DNA contents were determined by fluorescence measurements using BD FACS Aria™ III sorter (Becton, Dickinson and Company, Franklin Lakes, NJ, USA) using a PE configuration (547 nm excitation laser line; emission: 585 nm). At least 1 × 10^4^ cells were analyzed for each sample, recording the DNA content as a differentiating parameter for mononuclear cells (G0/G1 phase); S (DNA replication phase); G2/M (binucleate and mitotic fraction) or dead, necrotic and apoptotic cells (sub-G1 fraction), respectively. The results were analyzed using the free software FlowingSoftware 2.5.1 (Perttu Terho, Turku Centre for Biotechnology, University of Turku, Finland) and are presented as mean fluorescence.

#### 4.3.4. Statistical Analysis

Obtained absorbance was analysed to assess proliferation and survival fraction (SF), where tested samples were compared with the untreated controls (100%). Using Excel (Microsoft Office 365, A3 for faculty, access date: 2020), an IC_50_ index was calculated for tested compounds/drugs, where used concentration reduced viability of treated populations by 50% in comparison to the untreated controls. The results from MTT and cell cycle were presented as mean from three experiments, and ± SD was added. The statistical significance was calculated with T-test and indicated by a star on the charts (*p* value < 0.05).

## Figures and Tables

**Table 1:** Reaction conditions to obtain azide derivatives of sugars (**1**–**6**).

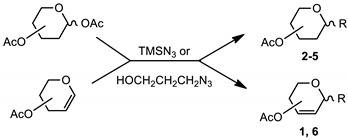				
R	Conditions	Product	Yield [%]	α/β
-OCH_2_CH_2_CH_2_N_3_	HO(CH_2_)_3_N_3_, FeCl_3_, DCM, 1 h—r.t.	**1**	87%	1:6
-OCH_2_CH_2_CH_2_N_3_	HO(CH_2_)_3_N_3_, BF_3_OEt_2_, DCM, 2 h—0 °C, 8 h—r.t.	**2**	82%	0:100
-N_3_	TMSN_3_, SnCl_4_, DCM, 24 h—r.t.	**3**	97%	0:100
-N_3_		**4**	98%	0:100
-N_3_		**5**	97%	1:8
-N_3_	TMSN_3_, I_2_, DCM, 8 h—0 °C-r.t.	**6**	78%	1:1.5

**Table 2:** IC_50_ calculated for HCT116, and MCF-7 cell lines after 72 h of incubation with tested compounds (mean value from 3 experiments ± SD).

Compound	IC_50_(μM)	
	HCT116	MCF-7
**10**	No effect *	29.9 ± 0.1
**11**	14.6 ± 3.7	No effect *
**12**	9.1 ± 2.4	10.8 ± 0.7
**13**	166.4 ± 0.7	212.1 ± 1.0
**14**	39.2 ± 17.8	11.9 ± 0.02
**15**	31.4 ± 6.4	No effect *
**16**	19.2 ± 3.3	19.7 ± 8.8
**17**	No effect *	258.9 ± 2.2
**18**	13.0 ± 4.3	11.1 ± 1.9
**19**	31.4 ± 6.4	No effect *
**20**	70.4 ± 10.8	No effect *
**21**	14.4 ± 4.3	8.9 ± 0.05
**22**	14.3 ± 1.3	35.6 ± 0.03
**23**	No effect *	No effect *
**24**	15.0 ± 2.2	12.5 ± 2.7
**25**	14.0 ± 0.2	19.0 ± 0.9
**26**	9.2 ± 0.6	17.0 ± 1.0
**MCA ^a^**	8.3 ± 1.1	32.3 ± 4.4

* No effect—viability of cells (SF) similar to the control, about 100%; see Figure S1 (Supplementary File), MCA—mucochloric acid, ^a^ IC_50_ values from previous studies [20].

## Data Availability

Data are contained within the article.

## Supplementary Materials

The following supporting information can be downloaded at: https://www.mdpi.com/article/10.3390/ph16040525/s1. 1H and 13C NMR spectra of all obtained compounds: Figure S1: Results of viability of HCT116 and MCF-7 cells after 72 h of incubation with compounds at a dose of 0–100 μM.
