# Supplementary material for: Glycoconjugates of Mucochloric Acid—Synthesis and Biological Activity
Source: Pharmaceuticals (Basel). 2023 Mar 31;16(4):525. doi: 10.3390/ph16040525 (PMC10143444; doi:10.3390/ph16040525)
Supplement: Supplementary file 1 [file pharmaceuticals-16-00525-s001.zip › pharmaceuticals-2297715-supplementary.pdf]

Supplementary Data

Glycoconjugates of mucochloric acid – synthesis and biological activity

Katarzyna Żurawska 1,2, Daria Burdalska 1, Magdalena Skonieczna 2,3,*, Anna Byczek-Wyrostek 2, Anahit Dawicka 2,3, Anna Kasprzycka 1,2,* and Krzysztof Walczak 1

[1] Department of Organic Chemistry, Bioorganic Chemistry and Biotechnology, Faculty of Chemistry, Silesian University of Technology, Krzywoustego Street 4, 44-100 Gliwice, Poland

[2] Biotechnology Centre, The Silesian University of Technology, Krzywoustego Street 8, 44-100 Gliwice, Poland

[3] Department of Systems Biology and Engineering, The Silesian University of Technology, Akademicka Street 16, 44-100 Gliwice, Poland

*Correspondence: magdalena.skonieczna@polsl.pl (M.S.); anna.kasprzycka@polsl.pl (A.K.)

Content:

1. **NMR spectra** **S2**
2. **Figure S1.** Survival fraction of HCT116 and MCF7 cell lines after 72-hours of incubation with tested compounds, evaluated by MTT assay. **S17**

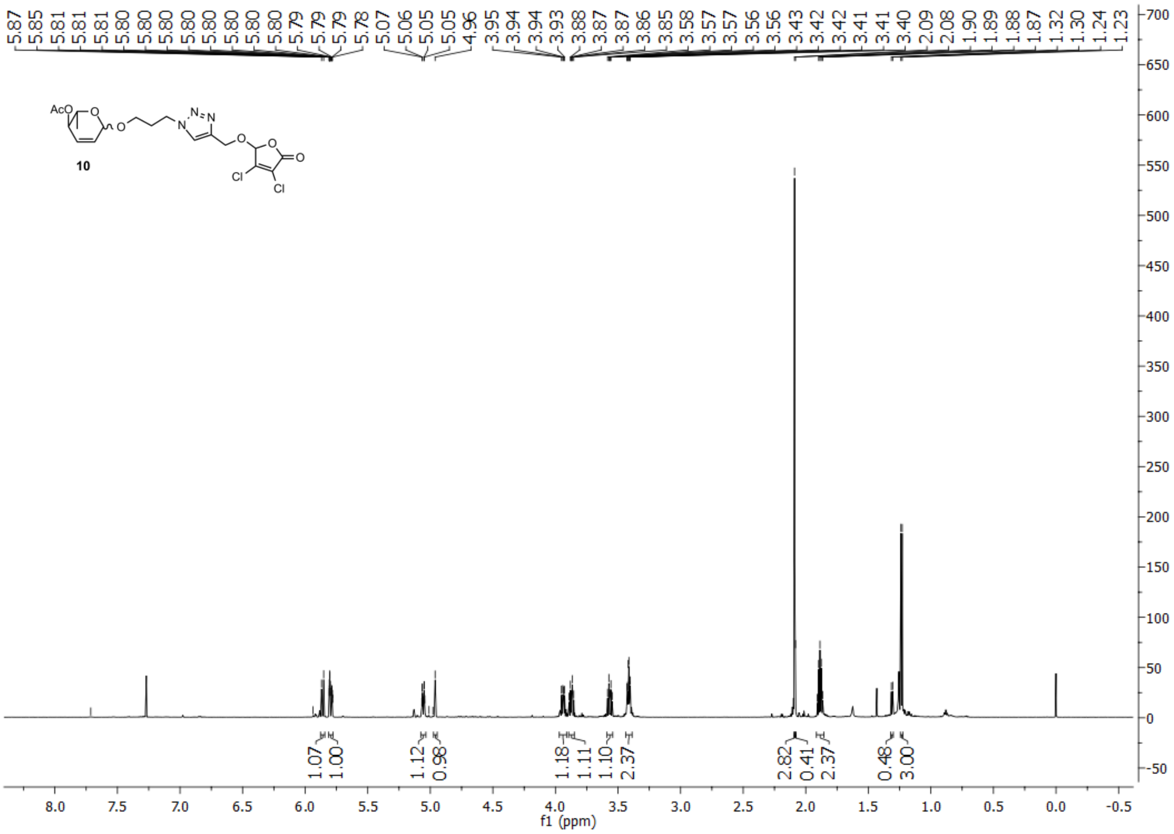
5.87 5.85 5.81 5.81 5.80 5.80 5.80 5.80 5.80 5.80 5.80 5.79 5.79 5.78 5.07 5.06 5.05 5.05 4.96 3.95 3.94 3.94 3.93 3.88 3.87 3.87 3.86 3.85 3.58 3.57 3.57 3.56 3.56 3.43 3.42 3.42 3.41 3.41 3.40 2.09 2.08 1.90 1.89 1.88 1.87 1.32 1.30 1.24 1.23
AcO
O
N
N
N
Cl
Cl
10
1.07 1.00 1.12 0.98 1.18 1.11 1.10 2.37 2.82 0.41 2.37 0.48 3.00
f1 (ppm)

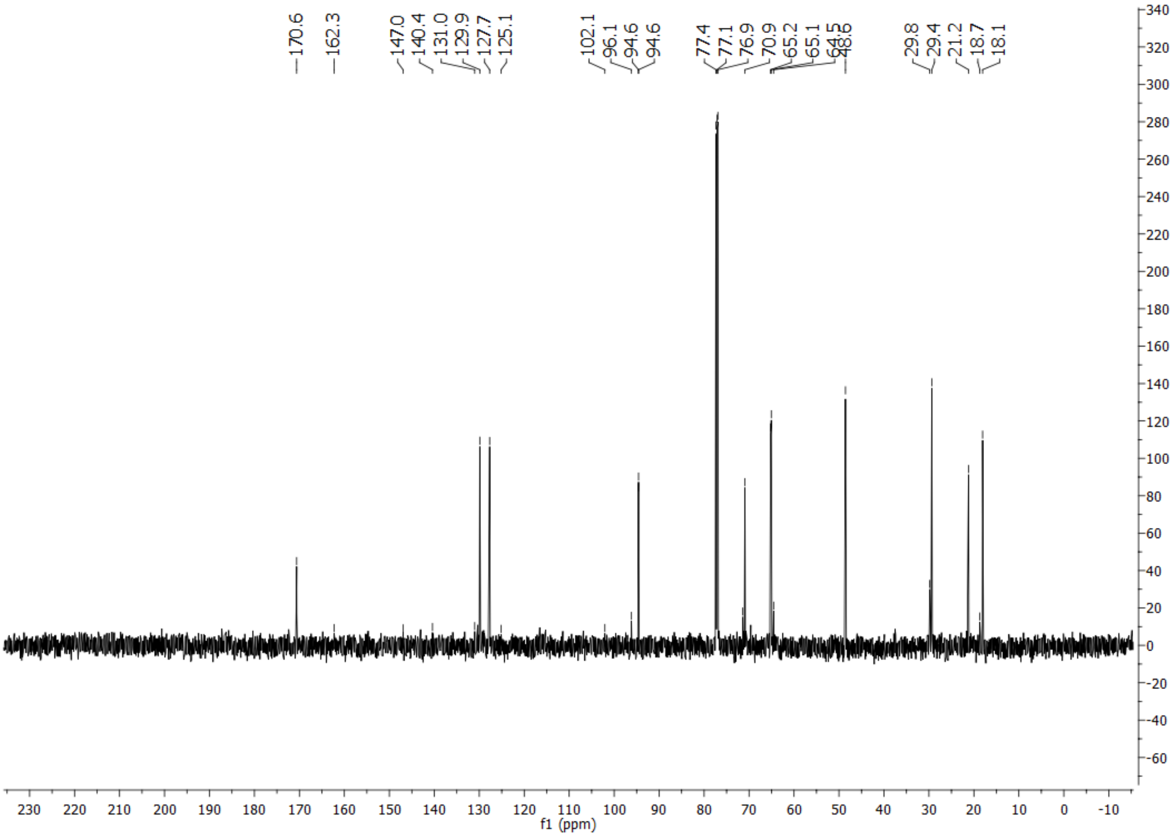
170.6 162.3 147.0 140.4 131.0 129.9 127.7 125.1 102.1 96.1 94.6 94.6 77.4 77.1 76.9 70.9 65.2 65.1 64.5 48.6 29.8 29.4 21.2 18.7 18.1
f1 (ppm)

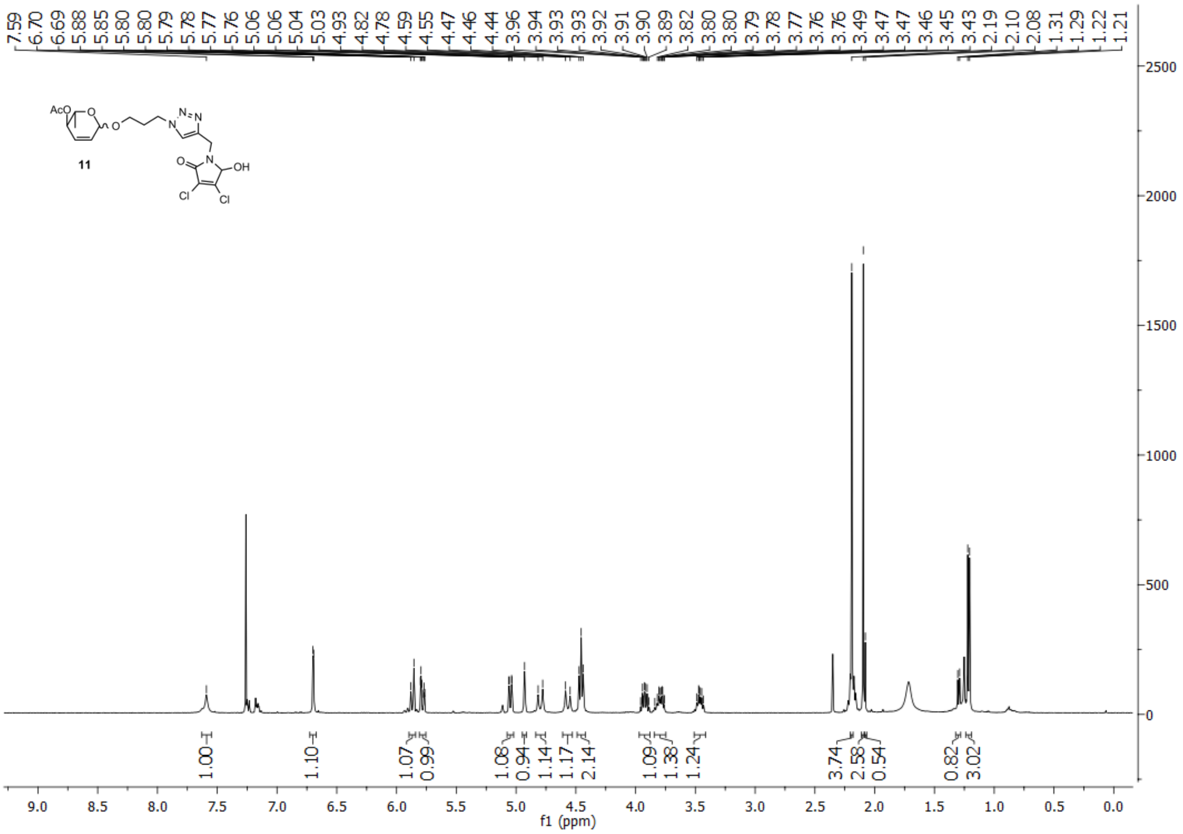
7.59 6.70 6.69 5.88 5.85 5.80 5.80 5.79 5.78 5.77 5.76 5.06 5.06 5.04 5.03 4.93 4.82 4.78 4.59 4.55 4.47 4.46 4.44 3.96 3.94 3.93 3.93 3.92 3.91 3.90 3.89 3.82 3.80 3.80 3.79 3.78 3.77 3.76 3.76 3.49 3.47 3.47 3.46 3.45 3.45 3.43 2.19 2.10 2.08 1.31 1.29 1.22 1.21
AcO
O
N
OH
Cl
Cl
11
1.00 1.10 1.07 0.99 1.08 0.94 1.14 1.17 2.14 1.09 1.38 1.24 3.74 2.58 0.54 0.82 3.02
f1 (ppm)

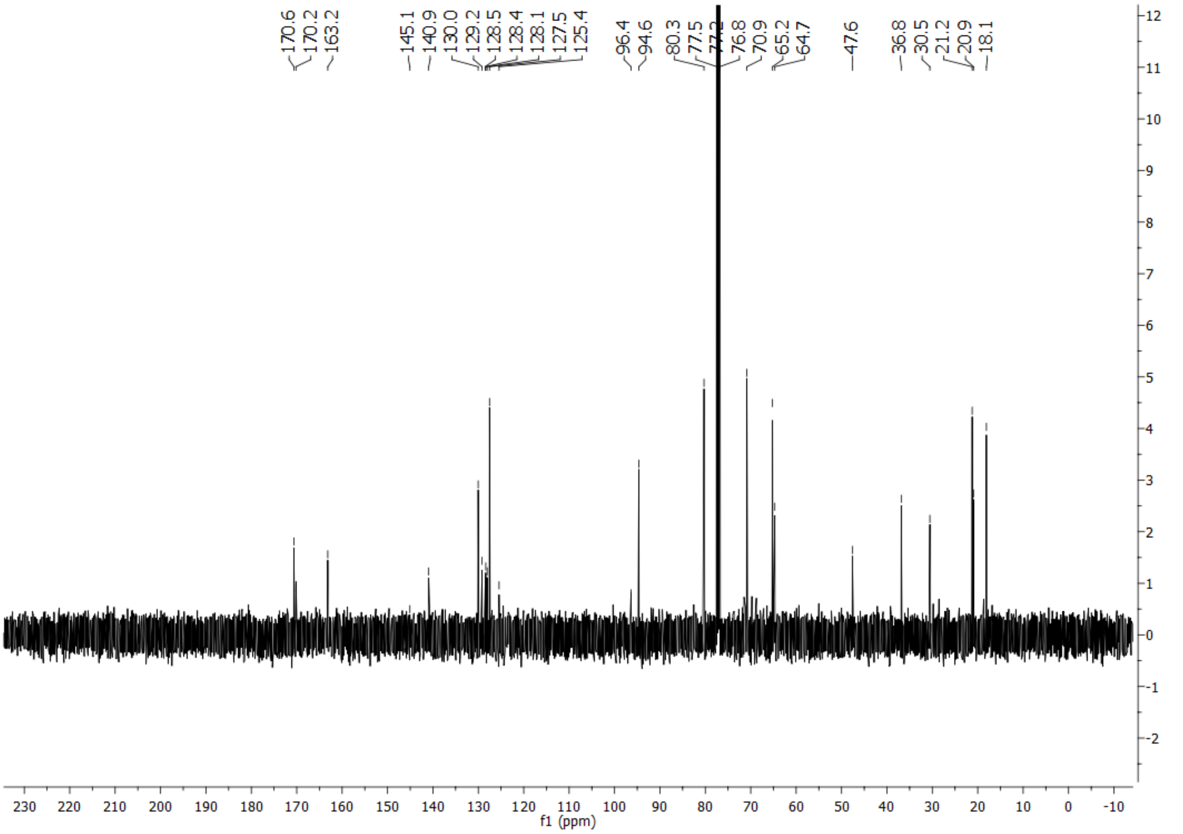
170.6 170.2 163.2 145.1 140.9 130.0 129.2 128.5 128.4 128.1 127.5 125.4 96.4 94.6 80.3 77.5 77.2 76.8 70.9 65.2 64.7 47.6 36.8 30.5 21.2 20.9 18.1
f1 (ppm)

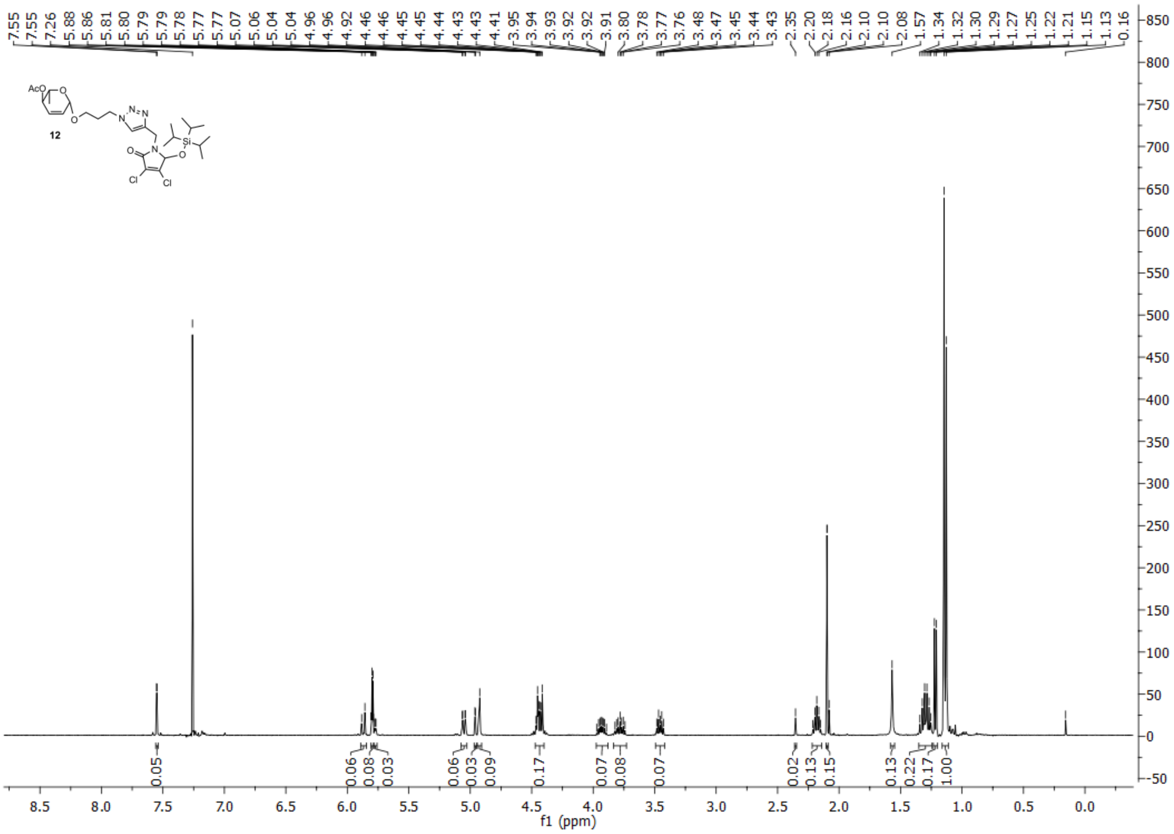
7.55 7.55 7.26 5.88 5.86 5.81 5.80 5.79 5.79 5.78 5.77 5.77 5.07 5.06 5.04 5.04 4.96 4.96 4.92 4.46 4.46 4.45 4.45 4.44 4.43 4.43 4.41 3.95 3.94 3.93 3.92 3.91 3.80 3.78 3.77 3.76 3.48 3.47 3.45 3.44 3.43 2.35 2.20 2.18 2.16 2.10 2.10 2.08 1.57 1.34 1.32 1.30 1.29 1.27 1.25 1.22 1.21 1.15 1.13 0.16
AcO
12
Si
Cl
Cl
0.05 0.06 0.08 0.03 0.06 0.03 0.09 0.17 0.07 0.08 0.07 0.02 0.13 0.15 0.13 0.22 0.17 1.00
f1 (ppm)

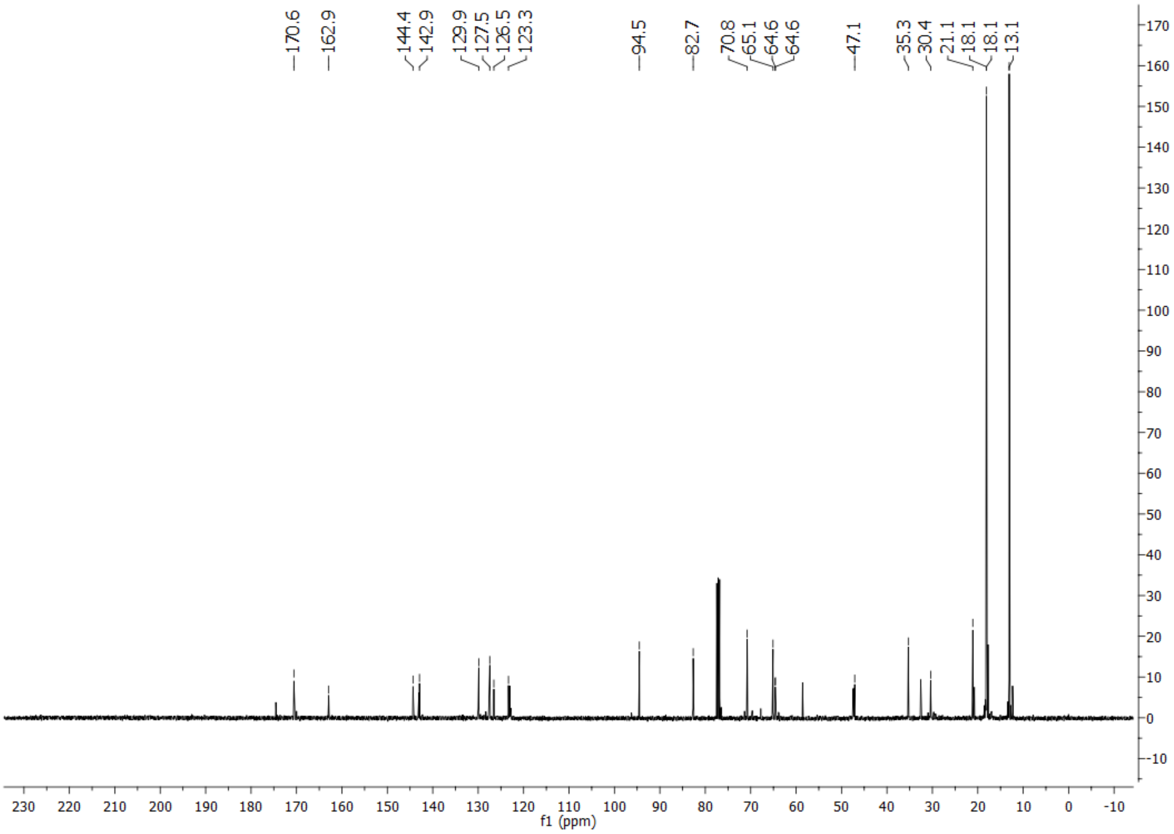
170.6 162.9 144.4 142.9 129.9 127.5 126.5 123.3 94.5 82.7 70.8 65.1 64.6 64.6 47.1 35.3 30.4 21.1 18.1 18.1 13.1
f1 (ppm)

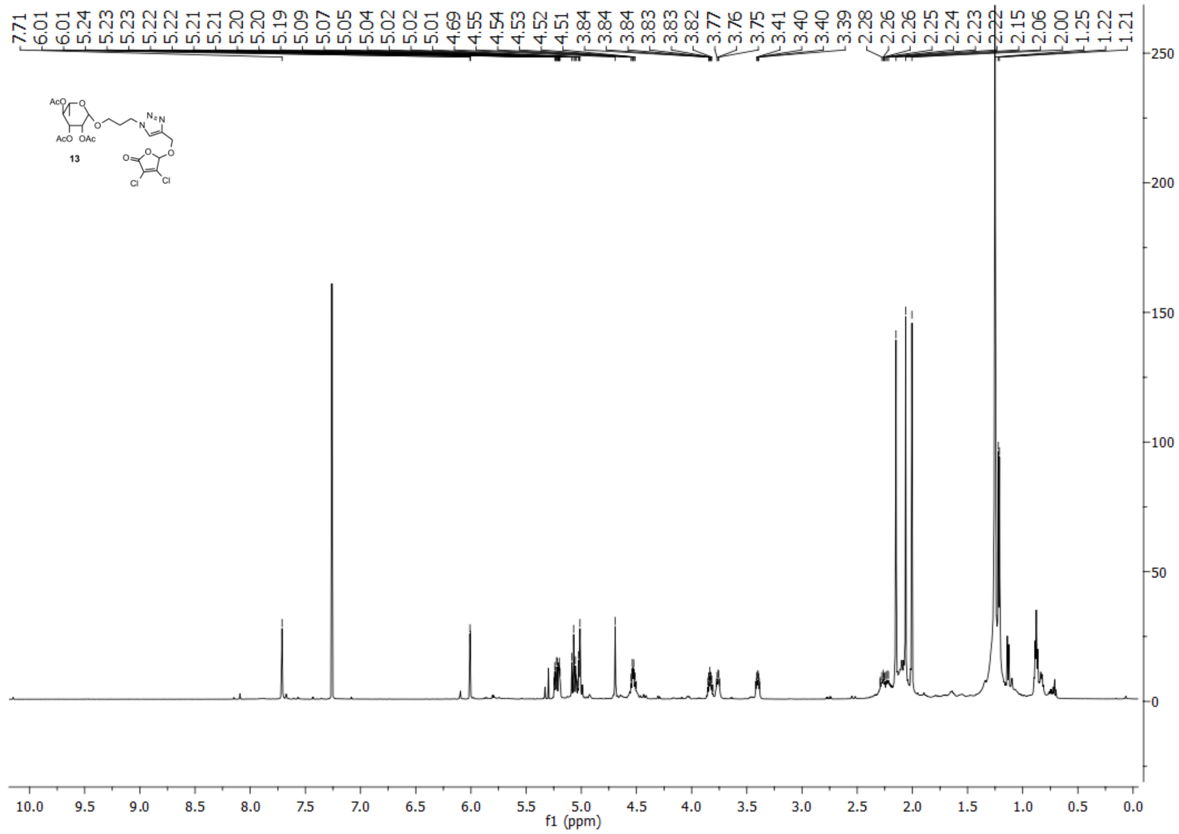
7.71 6.01 6.01 5.24 5.23 5.23 5.22 5.22 5.21 5.21 5.20 5.20 5.19 5.09 5.07 5.05 5.04 5.02 5.02 5.01 4.69 4.55 4.54 4.53 4.52 4.51 3.84 3.84 3.84 3.83 3.83 3.82 3.77 3.76 3.75 3.41 3.40 3.40 3.39 2.28 2.26 2.26 2.25 2.24 2.23 2.22 2.15 2.06 2.00 1.25 1.22 1.21
AcO
AcO OAc
13
Cl
Cl
f1 (ppm)

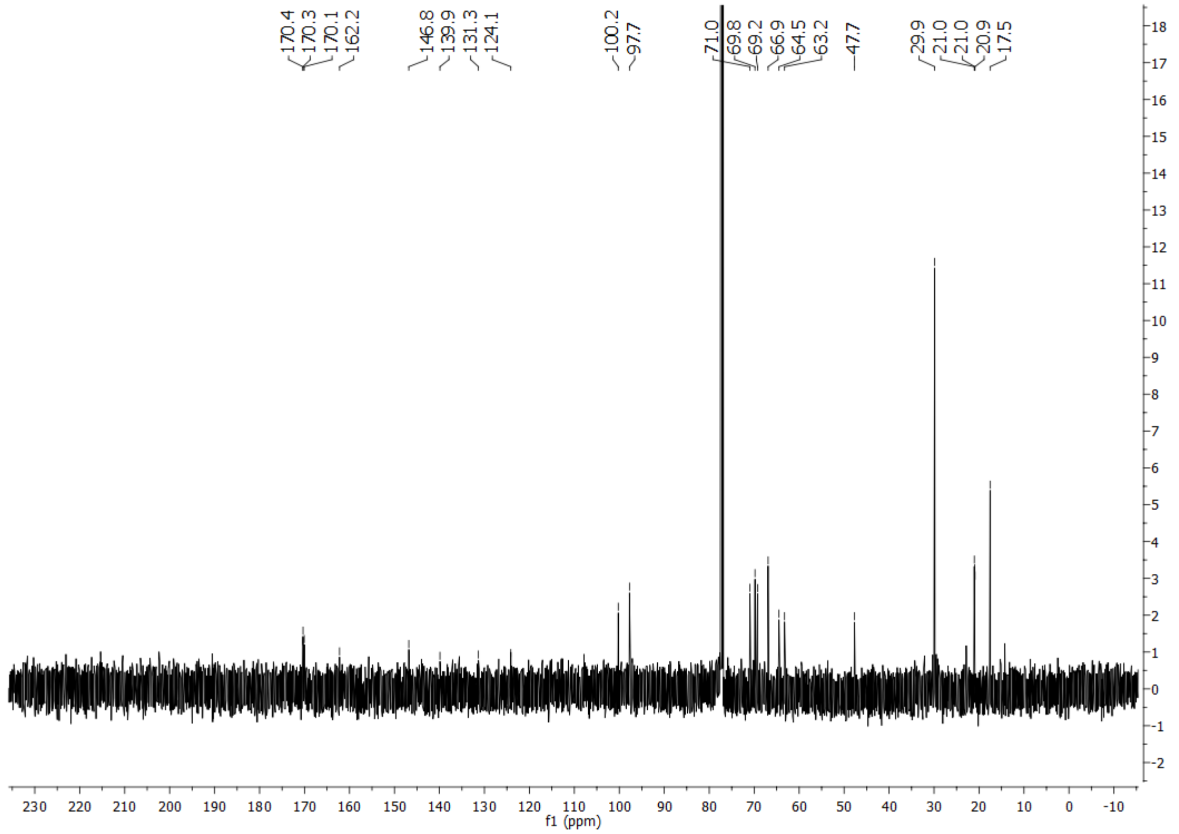
170.4 170.3 170.1 162.2 146.8 139.9 131.3 124.1 100.2 97.7 71.0 69.8 69.2 66.9 64.5 63.2 47.7 29.9 21.0 21.0 20.9 17.5
f1 (ppm)

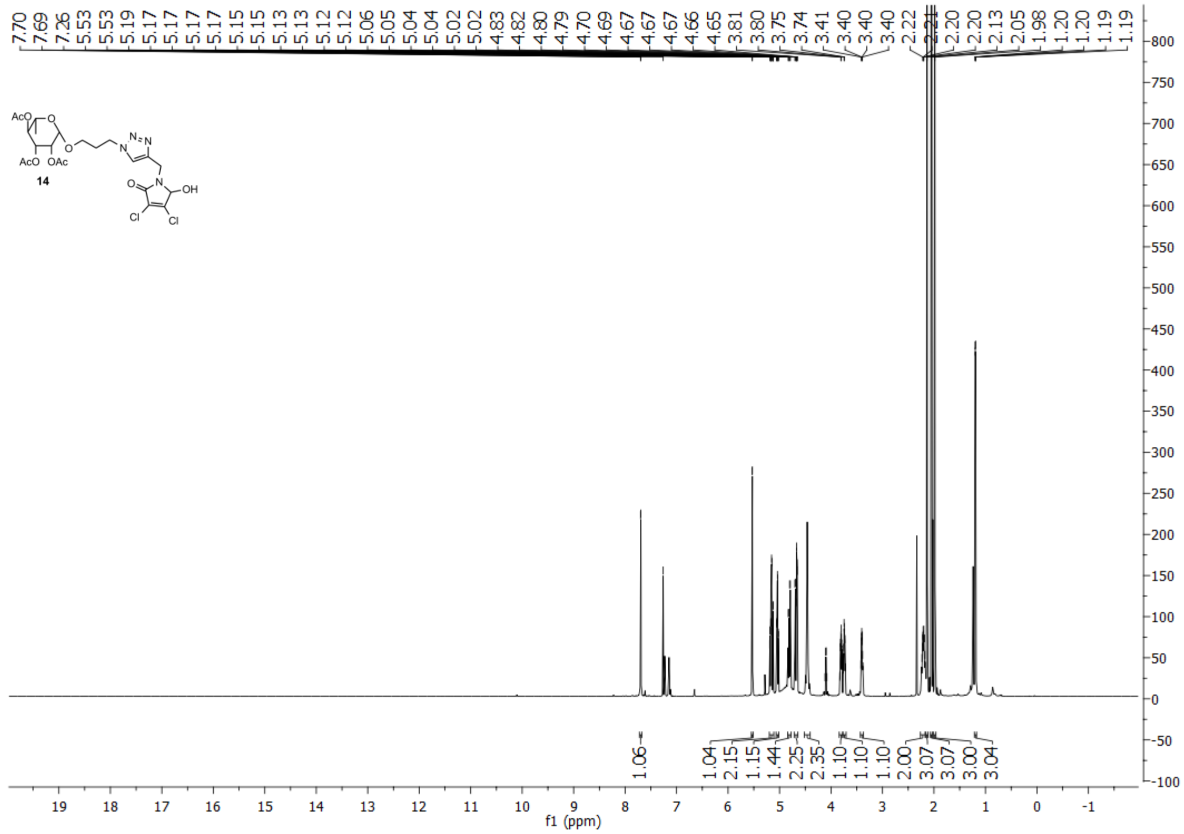
7.70
7.69
7.26
5.53
5.53
5.19
5.17
5.17
5.17
5.17
5.15
5.15
5.13
5.13
5.12
5.12
5.06
5.05
5.04
5.04
5.02
5.02
4.83
4.82
4.80
4.79
4.70
4.69
4.67
4.67
4.67
4.66
4.65
3.81
3.80
3.75
3.74
3.41
3.40
3.40
3.40
2.22
2.21
2.20
2.20
2.13
2.05
1.98
1.20
1.20
1.19
1.19
AcO
O
O
N
N
N
AcO OAc
14
O
N
OH
Cl
Cl
1.06
1.04
2.15
1.15
1.44
2.25
2.35
1.10
1.10
1.10
2.00
3.07
3.07
3.00
3.04
f1 (ppm)

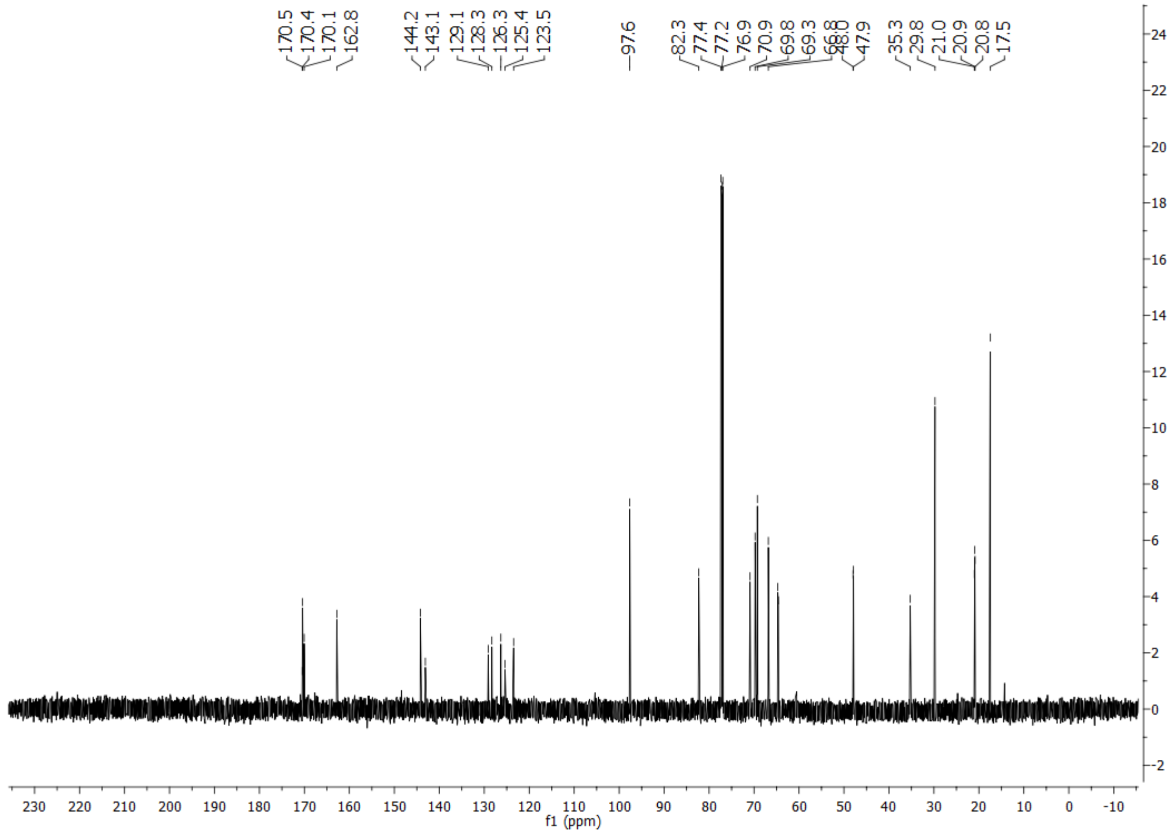
170.5
170.4
170.1
162.8
144.2
143.1
129.1
128.3
126.3
125.4
123.5
97.6
82.3
77.4
77.2
76.9
70.9
69.8
69.3
66.8
48.0
47.9
35.3
29.8
21.0
20.9
20.8
17.5
f1 (ppm)

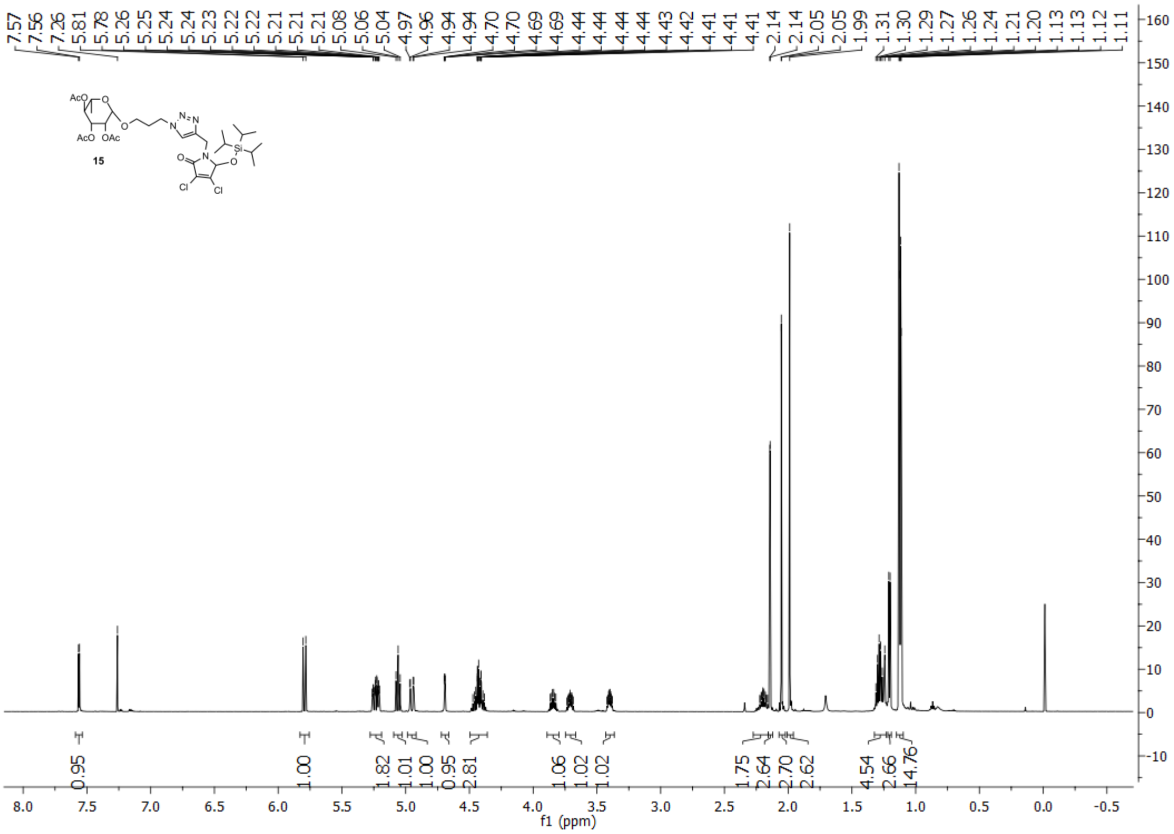
7.57 7.56 7.26 5.81 5.78 5.26 5.25 5.24 5.24 5.23 5.22 5.22 5.21 5.21 5.21 5.08 5.06 5.04 4.97 4.96 4.94 4.94 4.70 4.70 4.69 4.69 4.44 4.44 4.44 4.44 4.43 4.42 4.41 4.41 4.41 2.14 2.14 2.05 2.05 1.99 1.31 1.30 1.29 1.27 1.26 1.24 1.21 1.20 1.13 1.13 1.12 1.11
15
0.95 1.00 1.82 1.01 1.00 0.95 2.81 1.06 1.02 1.02 1.75 2.64 2.70 2.62 4.54 2.66 14.76
f1 (ppm)

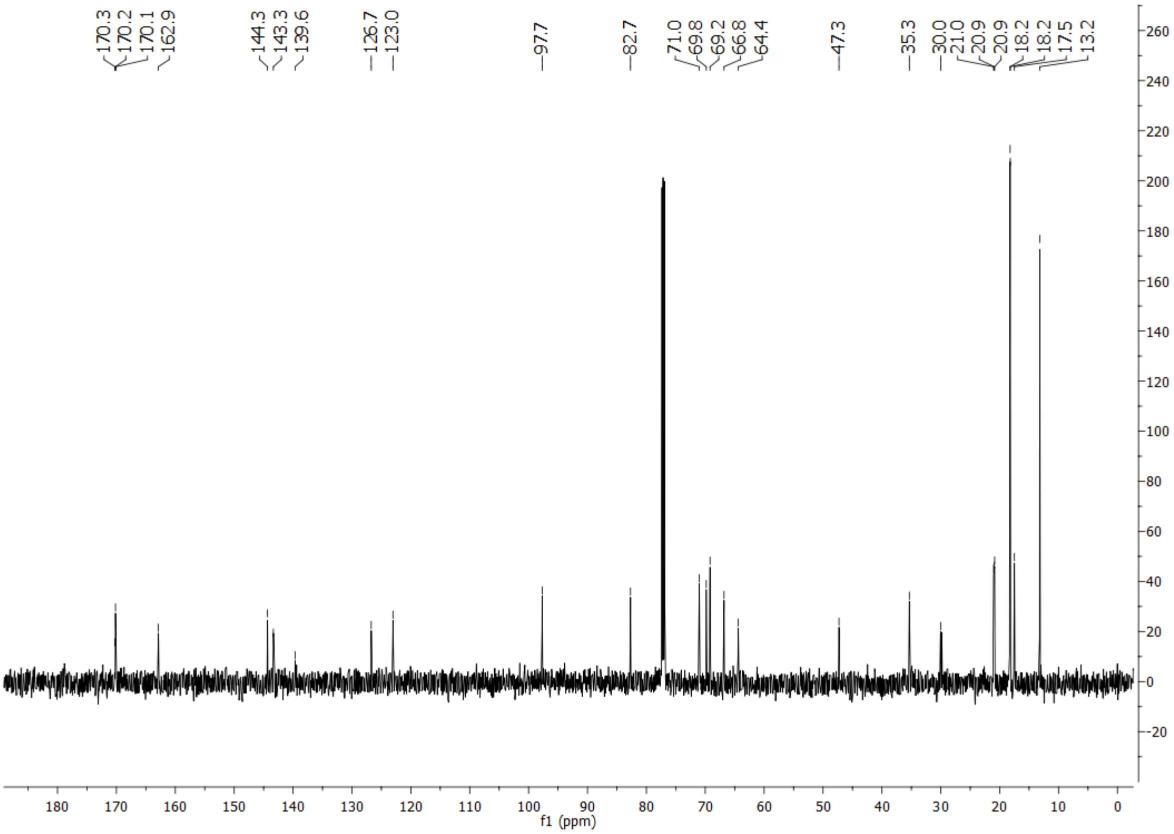
170.3 170.2 170.1 162.9 144.3 143.3 139.6 126.7 123.0 97.7 82.7 71.0 69.8 69.2 66.8 64.4 47.3 35.3 30.0 21.0 20.9 20.9 18.2 18.2 17.5 13.2
f1 (ppm)

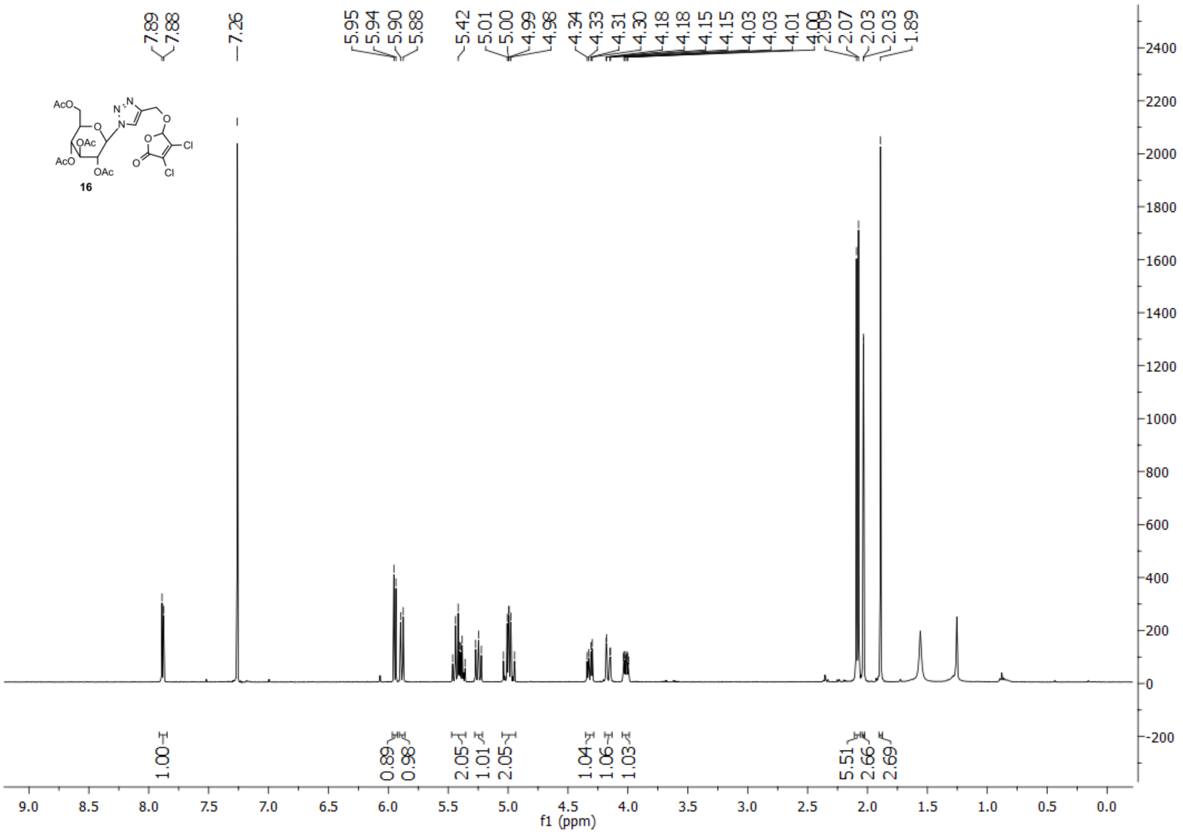
7.89
7.88
7.26
5.95
5.94
5.90
5.88
5.42
5.01
5.00
4.99
4.98
4.34
4.33
4.31
4.30
4.18
4.18
4.15
4.15
4.03
4.03
4.01
4.00
2.09
2.07
2.03
2.03
1.89
AcO
OAc
AcO
OAc
Cl
Cl
16
1.00
0.89
0.98
2.05
1.01
2.05
1.04
1.06
1.03
5.51
2.66
2.69
f1 (ppm)

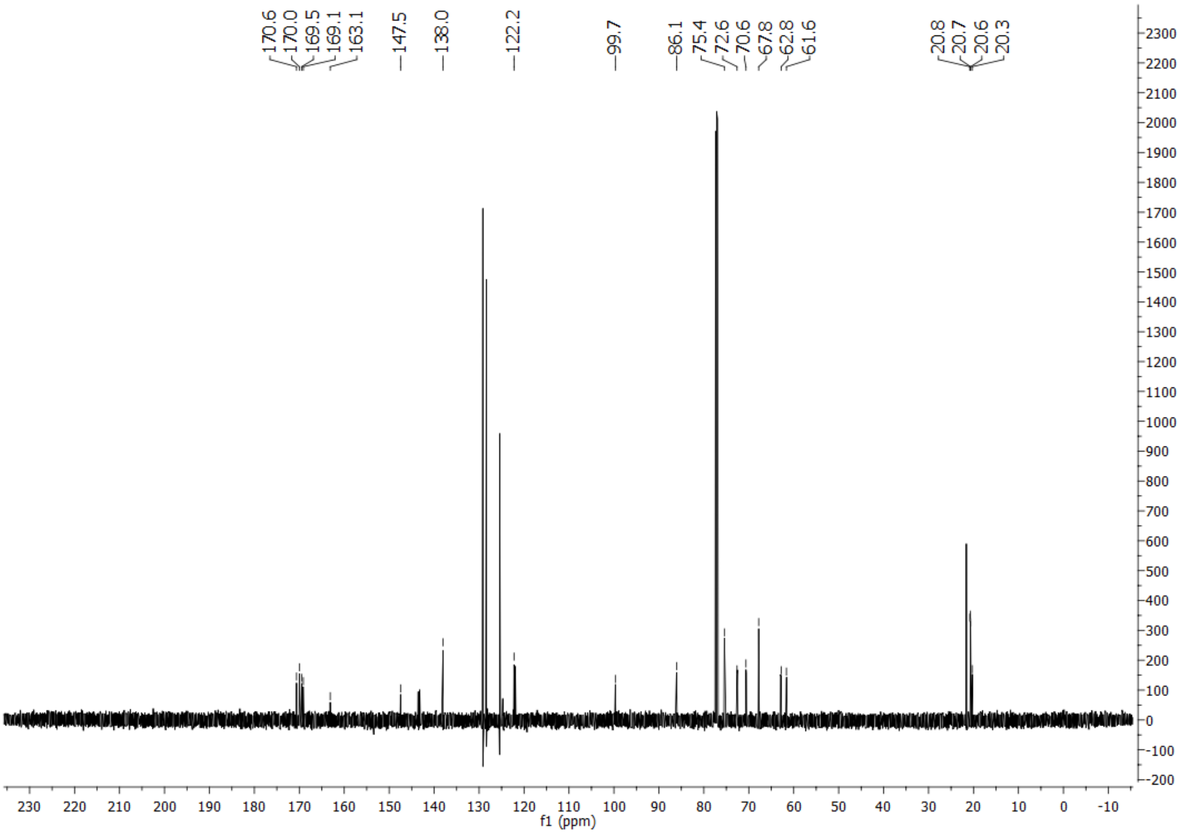
170.6
170.0
169.5
169.1
163.1
147.5
138.0
122.2
99.7
86.1
75.4
72.6
70.6
67.8
62.8
61.6
20.8
20.7
20.6
20.3
f1 (ppm)

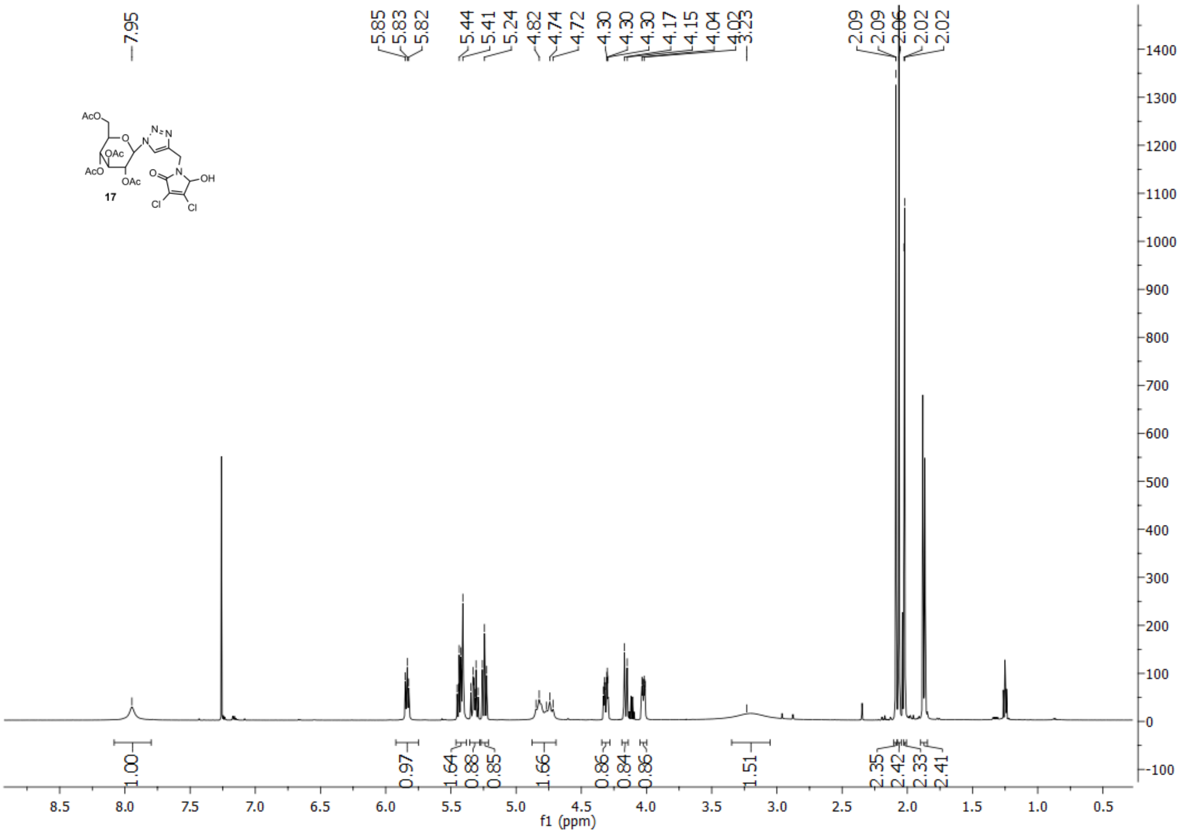
7.95
5.85
5.83
5.82
5.44
5.41
5.24
4.82
4.74
4.72
4.30
4.30
4.30
4.17
4.15
4.04
4.02
3.23
2.09
2.09
2.06
2.02
2.02
AcO
OAc
AcO
OAc
N
O
OH
Cl
Cl
17
1.00
0.97
1.64
0.88
0.85
1.66
0.86
0.84
0.86
1.51
2.35
2.42
2.33
2.41
f1 (ppm)

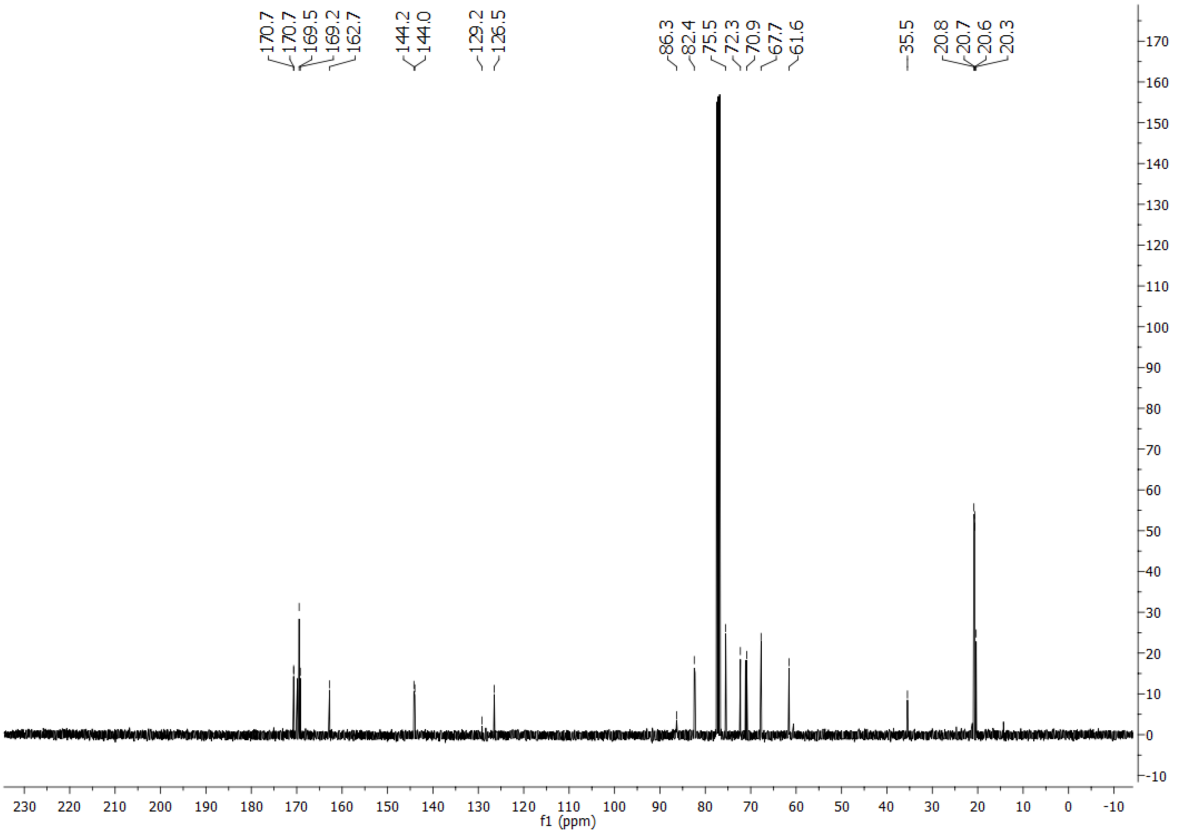
170.7
170.7
169.5
169.2
162.7
144.2
144.0
129.2
126.5
86.3
82.4
75.5
72.3
70.9
67.7
61.6
35.5
20.8
20.7
20.6
20.3
f1 (ppm)

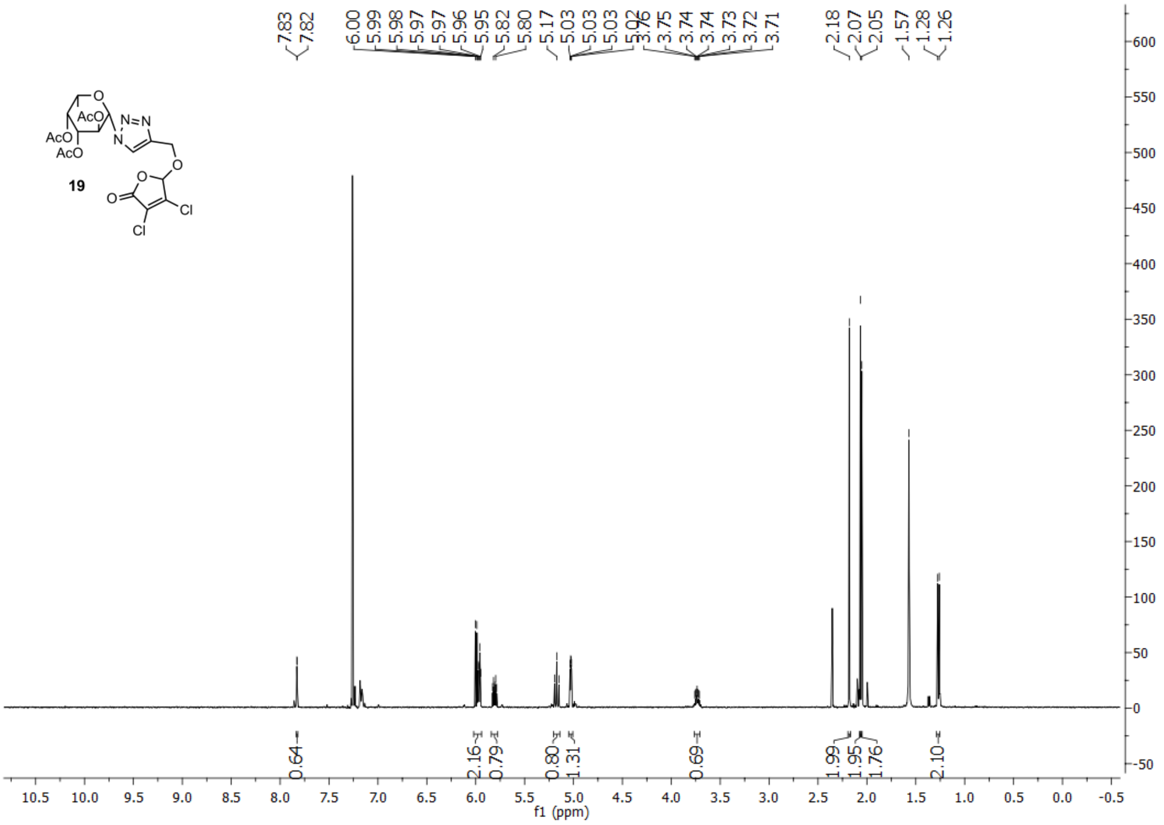

AcO
AcO
AcO
O
N=N
N
O
O
O
O
Cl
Cl
19
7.83
7.82
6.00
5.99
5.98
5.97
5.97
5.96
5.95
5.82
5.80
5.17
5.03
5.03
5.03
5.02
3.76
3.75
3.74
3.74
3.73
3.72
3.71
2.18
2.07
2.05
1.57
1.28
1.26
0.64
2.16
0.79
0.80
1.31
0.69
1.99
1.95
1.76
2.10
f1 (ppm)

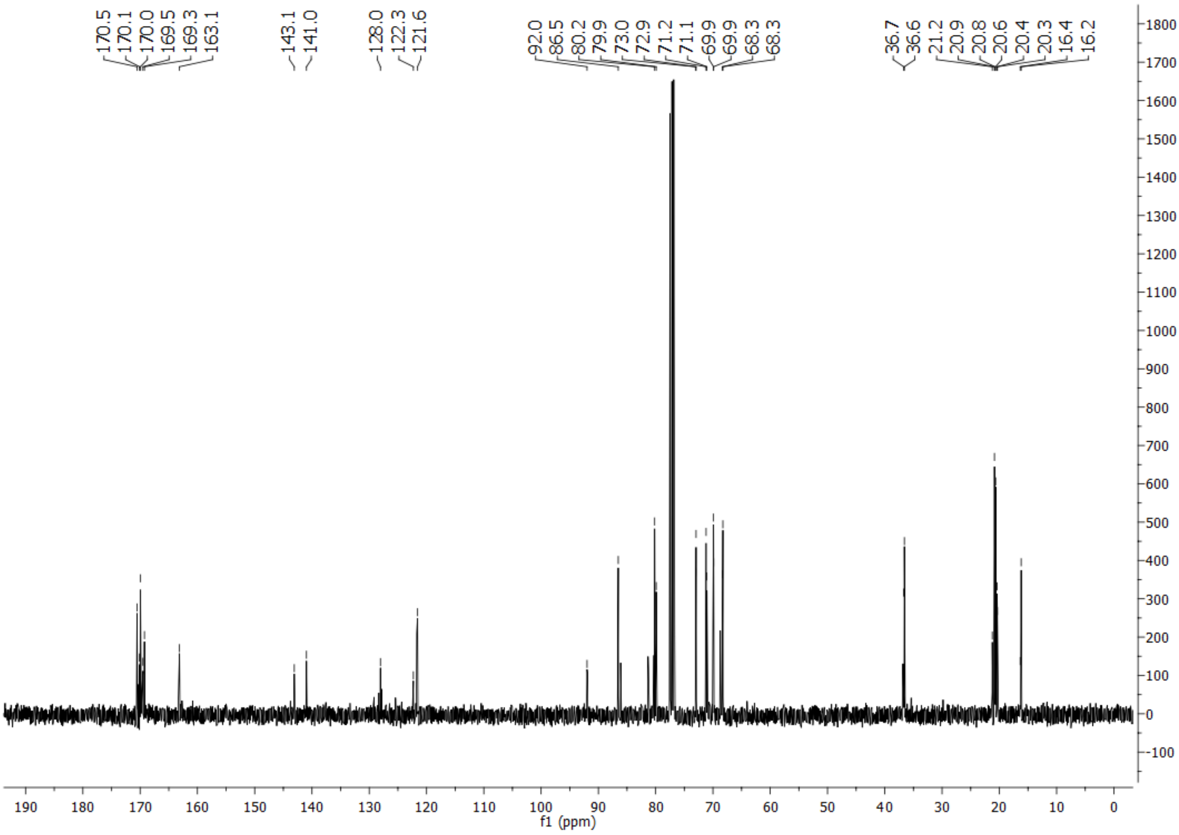

170.5
170.1
170.0
169.5
169.3
163.1
143.1
141.0
128.0
122.3
121.6
92.0
86.5
80.2
79.9
73.0
72.9
71.2
71.1
69.9
69.9
68.3
68.3
36.7
36.6
21.2
20.9
20.8
20.6
20.4
20.3
16.4
16.2
f1 (ppm)

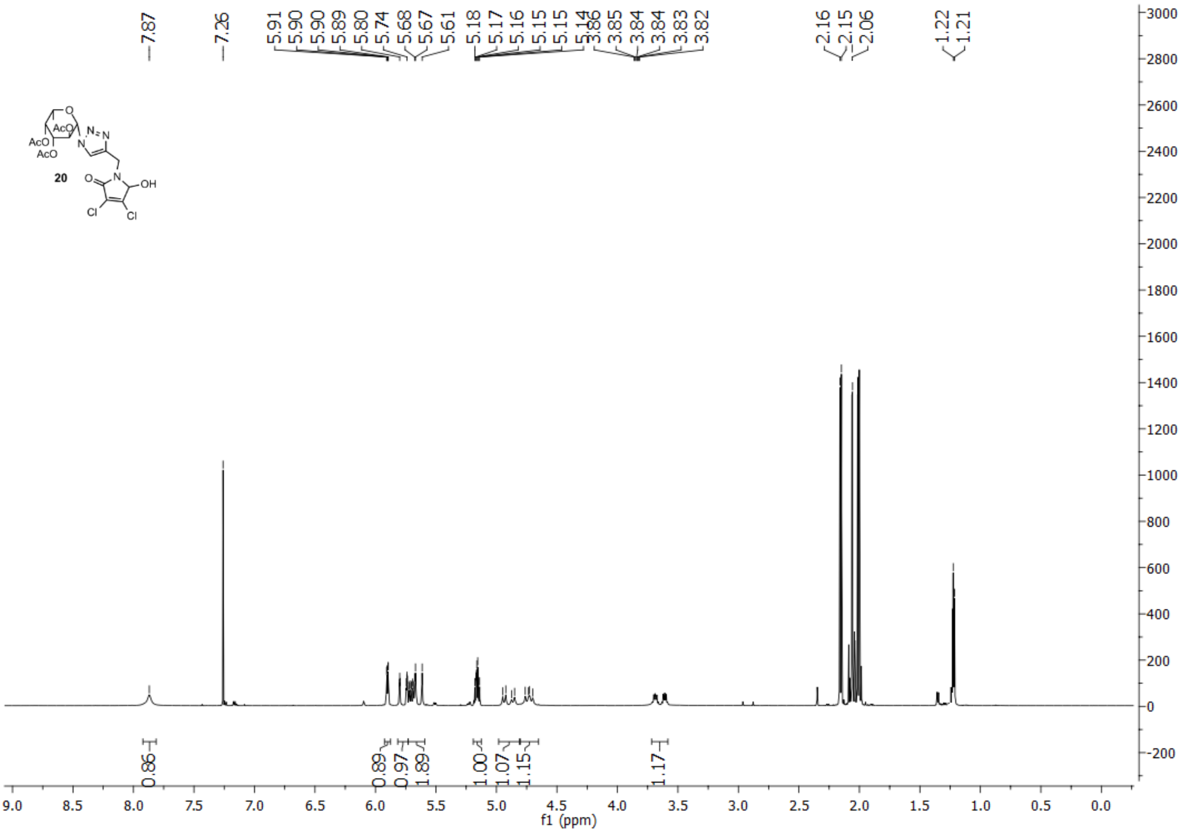
7.87
7.26
5.91 5.90 5.90 5.89 5.80 5.74 5.68 5.67 5.61
5.18 5.17 5.16 5.15 5.15 5.14
3.86 3.85 3.84 3.84 3.83 3.82
2.16 2.15 2.06
1.22 1.21
AcO
AcO
AcO
AcO
N
N
N
O
OH
Cl
Cl
20
0.86
0.89
0.97
1.89
1.00
1.07
1.15
1.17
f1 (ppm)

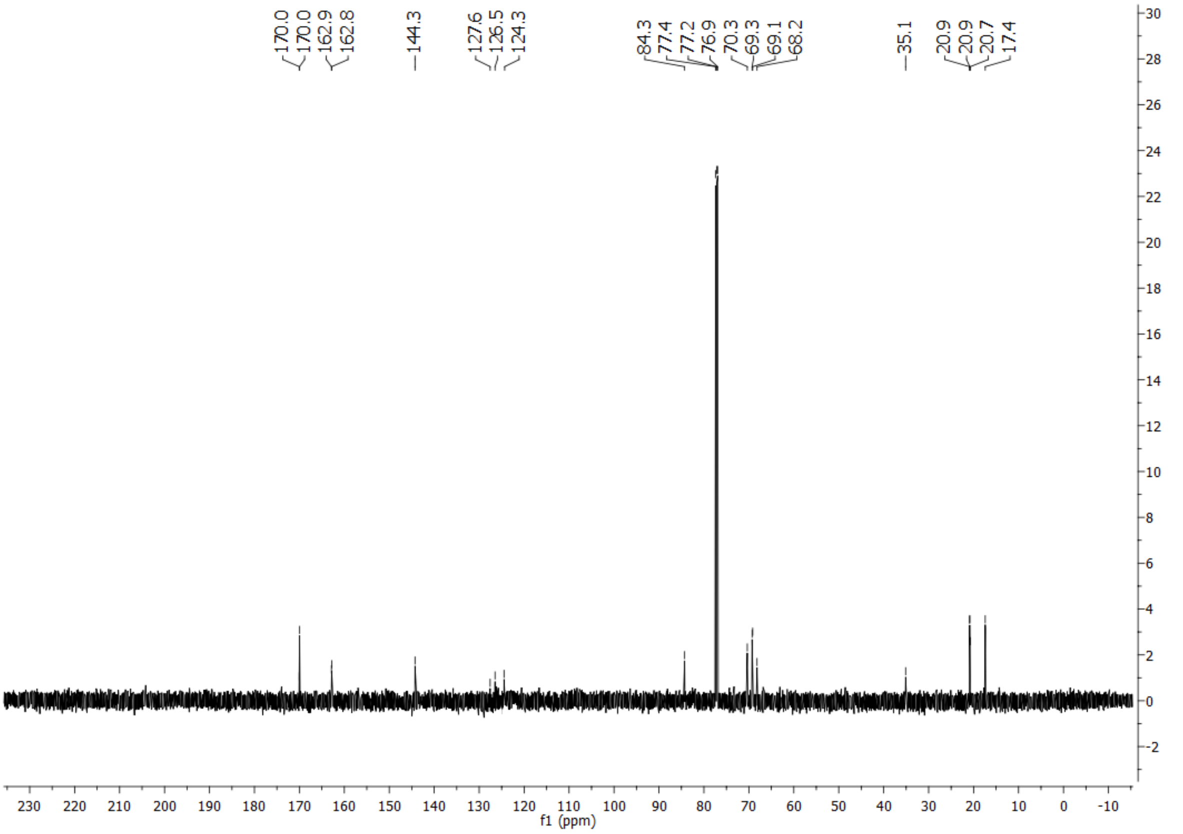
170.0 170.0 162.9 162.8
144.3
127.6 126.5 124.3
84.3 77.4 77.2 76.9 70.3 69.3 69.1 68.2
35.1
20.9 20.9 20.7 17.4
f1 (ppm)

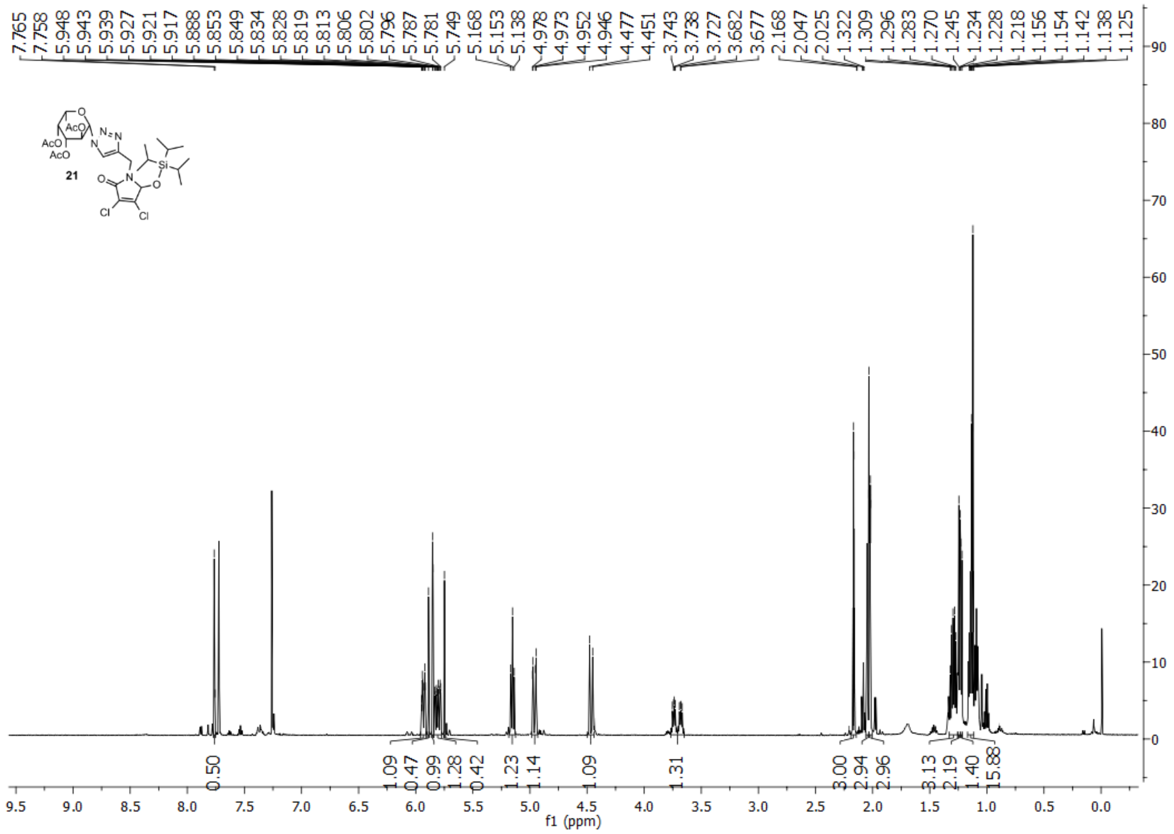
7.765
7.758
5.948
5.943
5.939
5.927
5.921
5.917
5.888
5.853
5.849
5.834
5.828
5.819
5.813
5.806
5.802
5.796
5.787
5.781
5.749
5.168
5.153
5.138
4.978
4.973
4.952
4.946
4.477
4.451
3.743
3.738
3.727
3.682
3.677
2.168
2.047
2.025
1.322
1.309
1.296
1.283
1.270
1.245
1.234
1.228
1.218
1.156
1.154
1.142
1.138
1.125
AcO
AcO
AcO
21
Si
Cl
Cl
0.50
1.09
0.47
0.99
1.28
0.42
1.23
1.14
1.09
1.31
3.00
2.94
2.96
3.13
2.19
1.40
15.88
f1 (ppm)

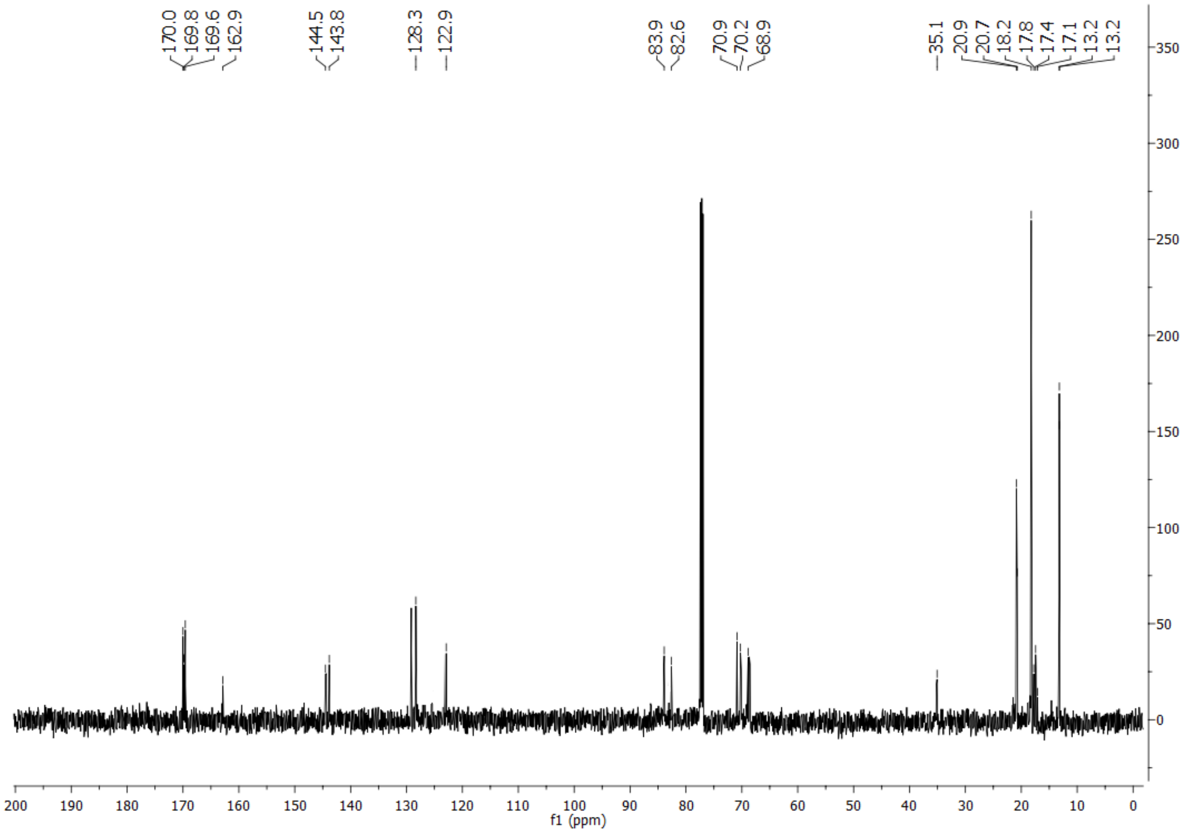
170.0
169.8
169.6
162.9
144.5
143.8
128.3
122.9
83.9
82.6
70.9
70.2
68.9
35.1
20.9
20.7
18.2
17.8
17.4
17.1
13.2
13.2
f1 (ppm)

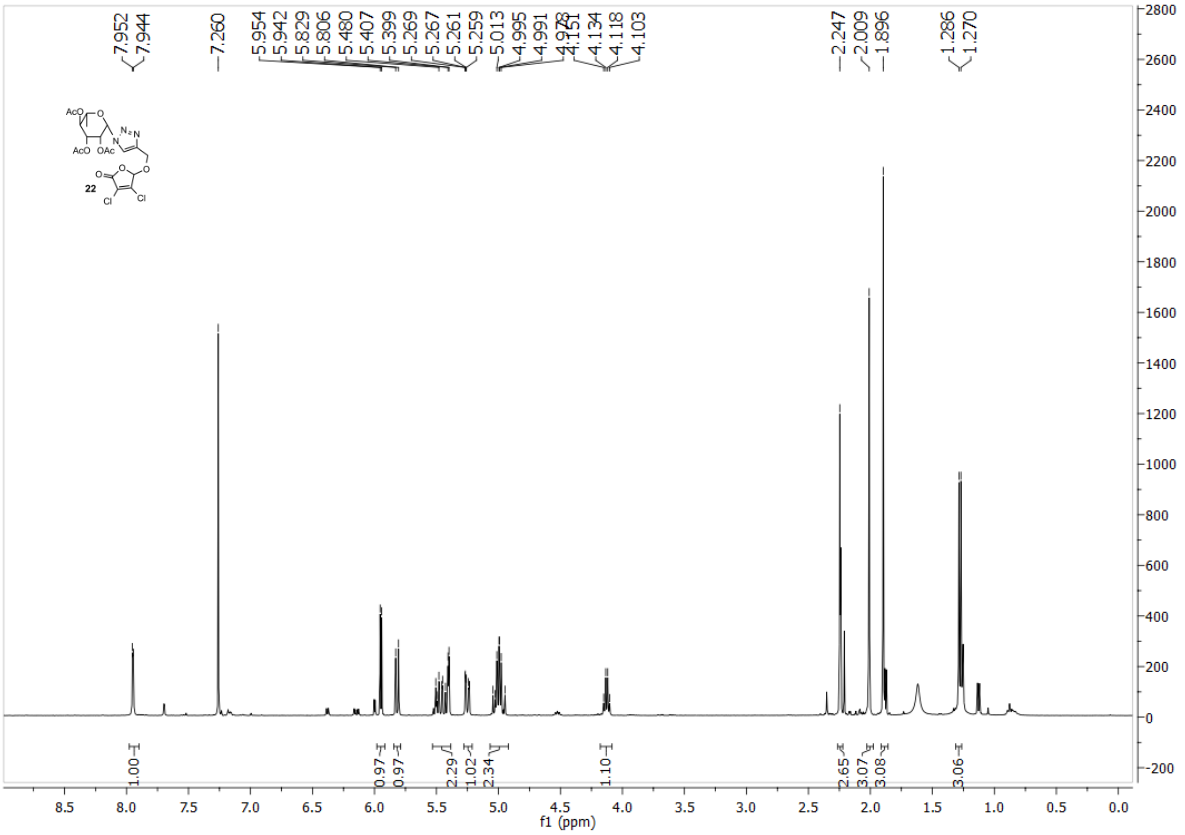
7.952
7.944
7.260
5.954
5.942
5.829
5.806
5.480
5.407
5.399
5.269
5.267
5.261
5.259
5.013
4.995
4.991
4.151
4.134
4.118
4.103
2.247
2.009
1.896
1.286
1.270
AcO
AcO
OAc
22
Cl
Cl
1.00
0.97
0.97
2.29
1.02
2.34
1.10
2.65
3.07
3.08
3.06
f1 (ppm)

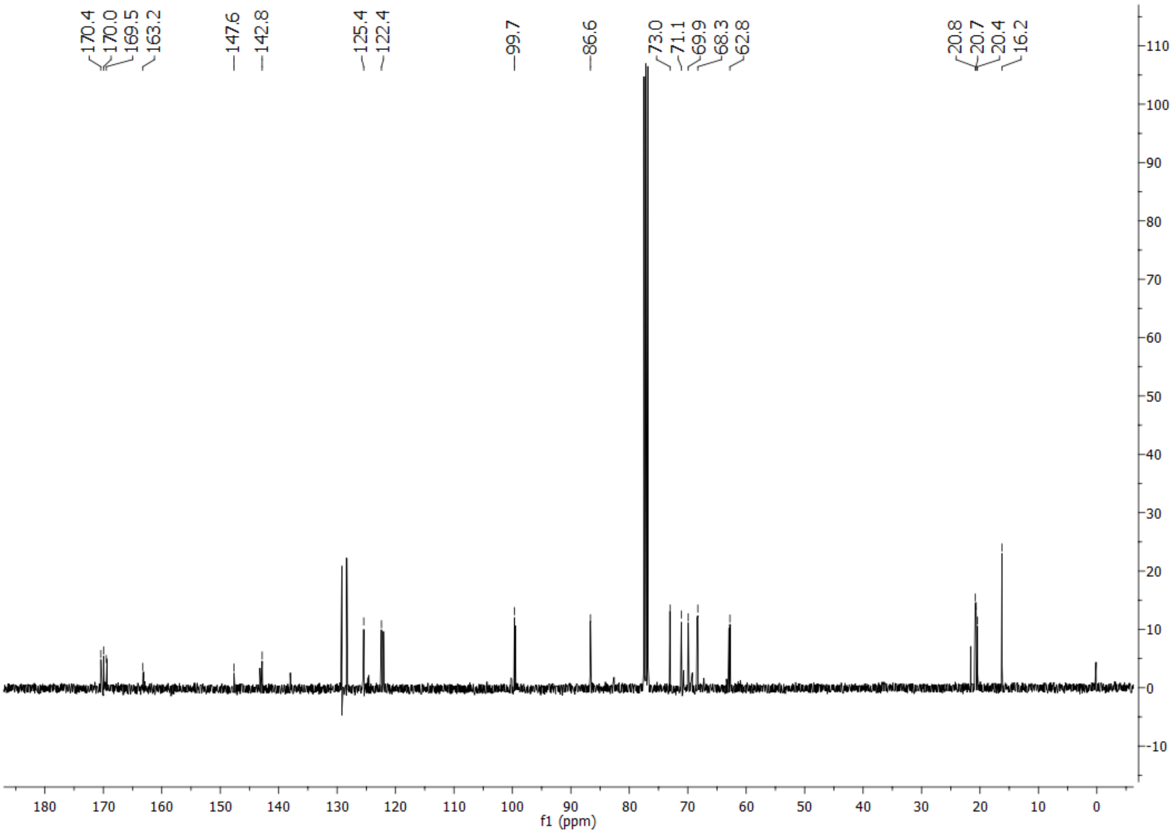
170.4
170.0
169.5
163.2
147.6
142.8
125.4
122.4
99.7
86.6
73.0
71.1
69.9
68.3
62.8
20.8
20.7
20.4
16.2
f1 (ppm)

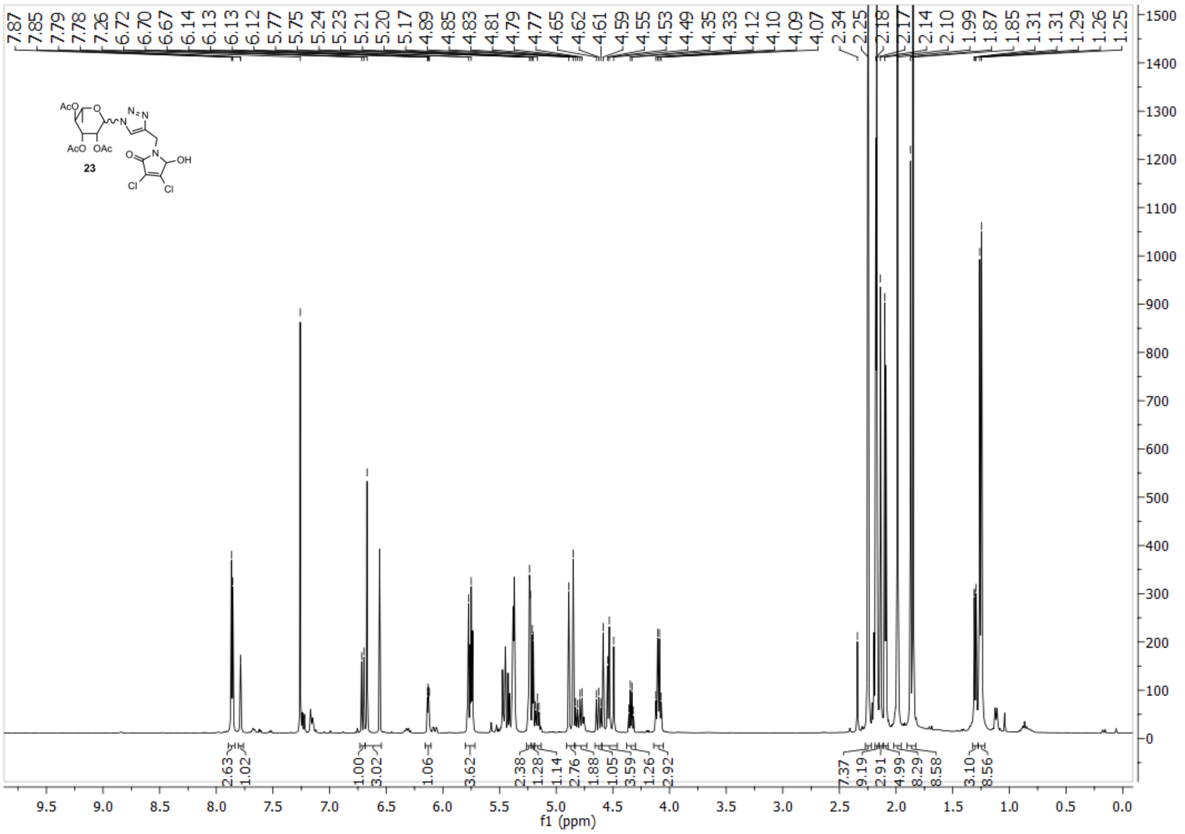
7.87 7.85 7.79 7.78 7.26 6.72 6.70 6.67 6.14 6.13 6.13 6.12 5.77 5.75 5.24 5.23 5.21 5.20 5.17 4.89 4.85 4.83 4.81 4.79 4.77 4.65 4.62 4.61 4.59 4.55 4.53 4.49 4.35 4.33 4.12 4.10 4.09 4.07 2.34 2.25 2.18 2.17 2.14 2.10 1.99 1.87 1.85 1.31 1.29 1.26 1.25
AcO
OAc
N
23
O
OH
Cl
2.63 1.02 1.00 3.02 1.06 3.62 2.38 1.28 1.14 2.76 1.88 1.05 3.59 1.26 2.92 7.37 9.19 2.91 4.99 8.29 8.58 3.10 8.56
f1 (ppm)

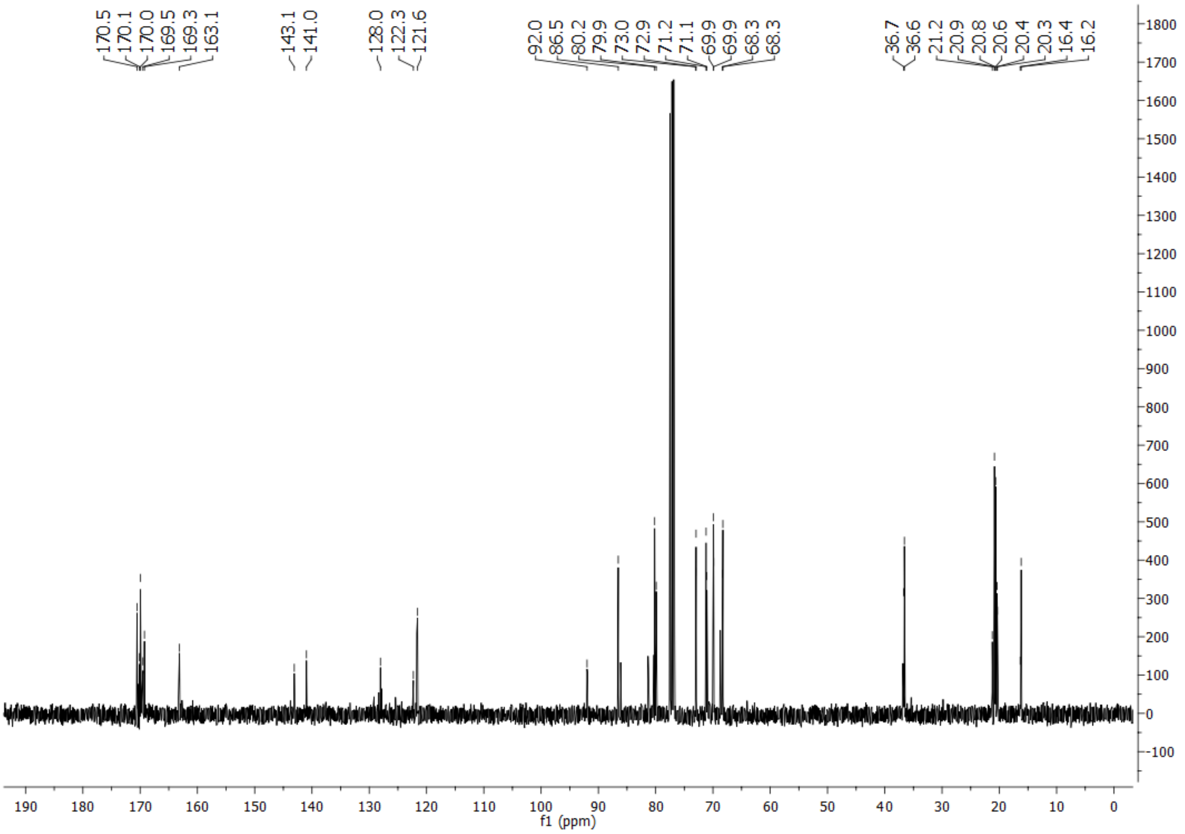
170.5 170.1 170.0 169.5 169.3 163.1 143.1 141.0 128.0 122.3 121.6 92.0 86.5 80.2 79.9 73.0 72.9 71.2 71.1 69.9 69.9 68.3 68.3 36.7 36.6 21.2 20.9 20.8 20.6 20.4 20.3 16.4 16.2
f1 (ppm)

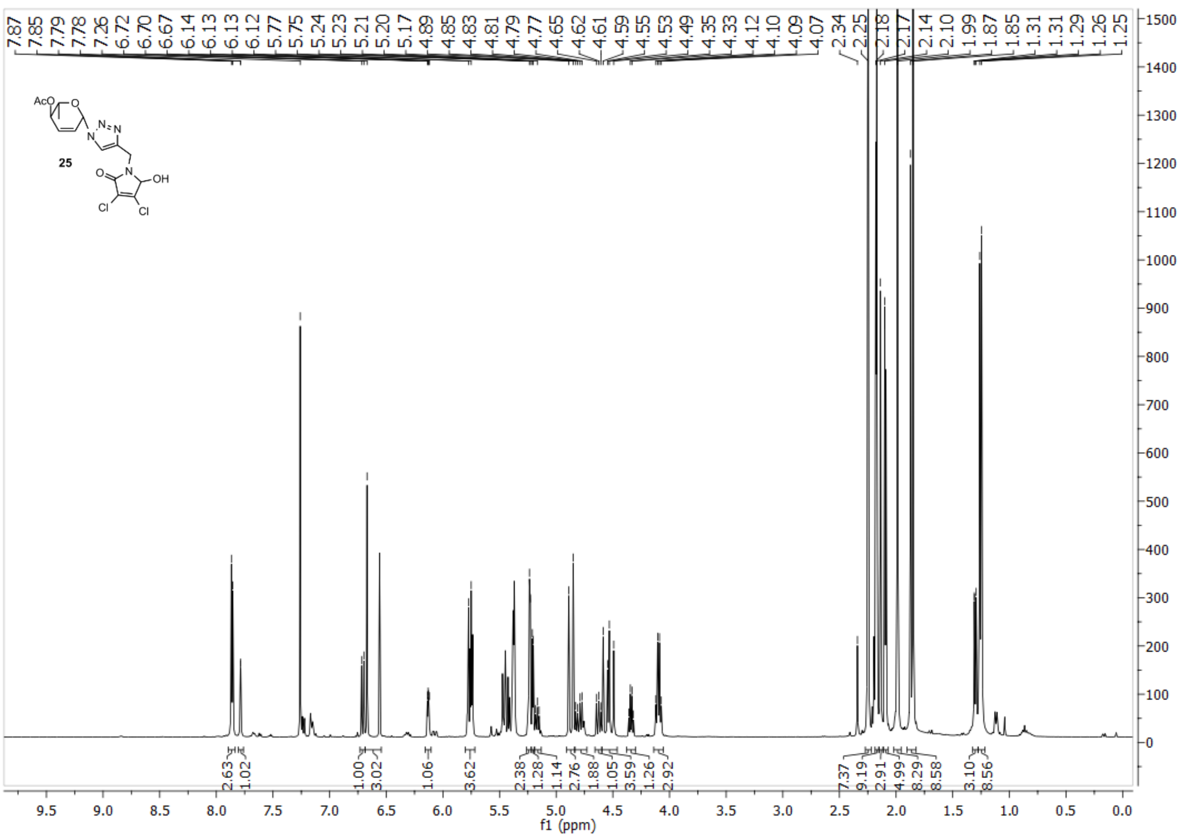
7.87 7.85 7.79 7.78 7.26 6.72 6.70 6.67 6.14 6.13 6.13 6.12 5.77 5.75 5.24 5.23 5.21 5.20 5.17 4.89 4.85 4.83 4.81 4.79 4.77 4.65 4.62 4.61 4.59 4.55 4.53 4.49 4.35 4.33 4.12 4.10 4.09 4.07 2.34 2.25 2.18 2.17 2.14 2.10 1.99 1.87 1.85 1.31 1.29 1.26 1.25
AcO
O
N
N
N
25
O
N
OH
Cl
Cl
2.63 1.02 1.00 3.02 1.06 3.62 2.38 1.28 1.14 2.76 1.88 1.05 3.59 1.26 2.92 7.37 9.19 2.91 4.99 8.29 8.58 3.10 8.56
f1 (ppm)

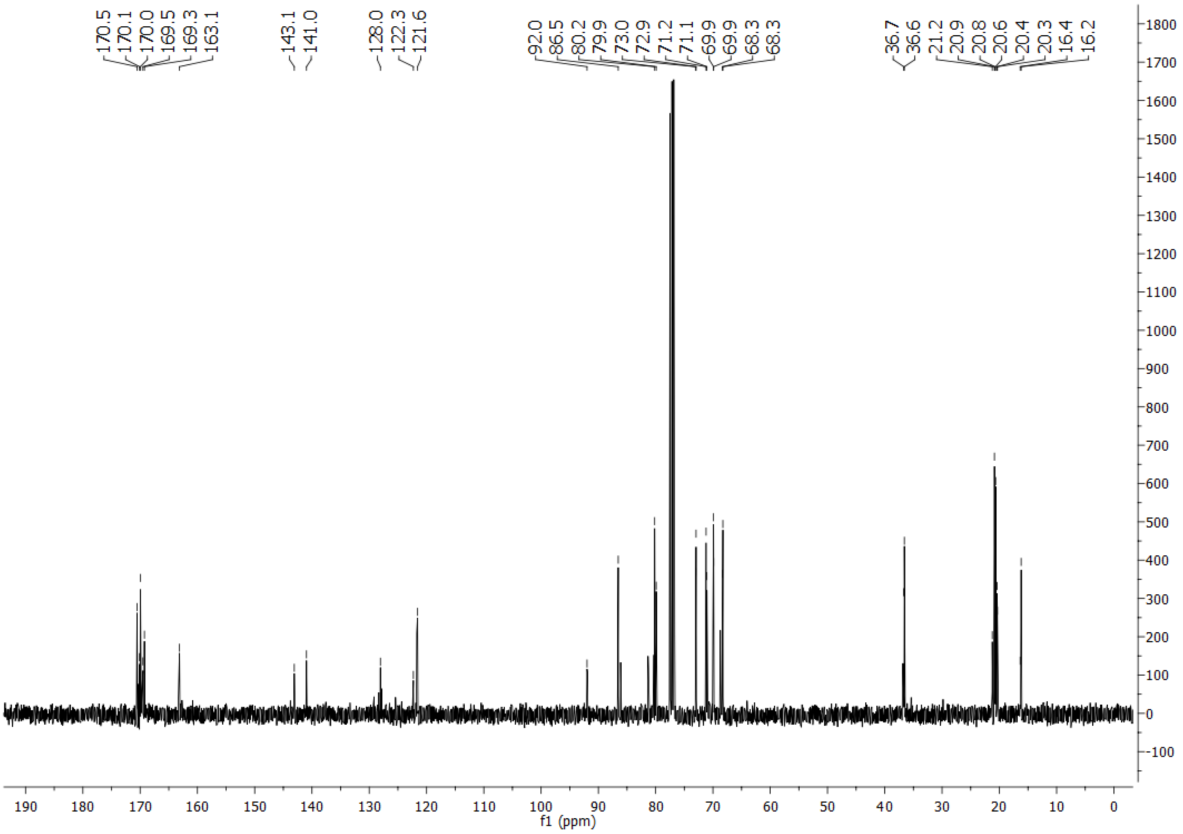
170.5 170.1 170.0 169.5 169.3 163.1 143.1 141.0 128.0 122.3 121.6 92.0 86.5 80.2 79.9 73.0 72.9 71.2 71.1 69.9 69.9 68.3 68.3 36.7 36.6 21.2 20.9 20.8 20.6 20.4 20.3 16.4 16.2
f1 (ppm)

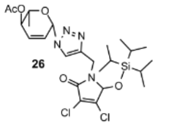
AcO
O
N
N
N
O
N
Si
O
Cl
Cl

26

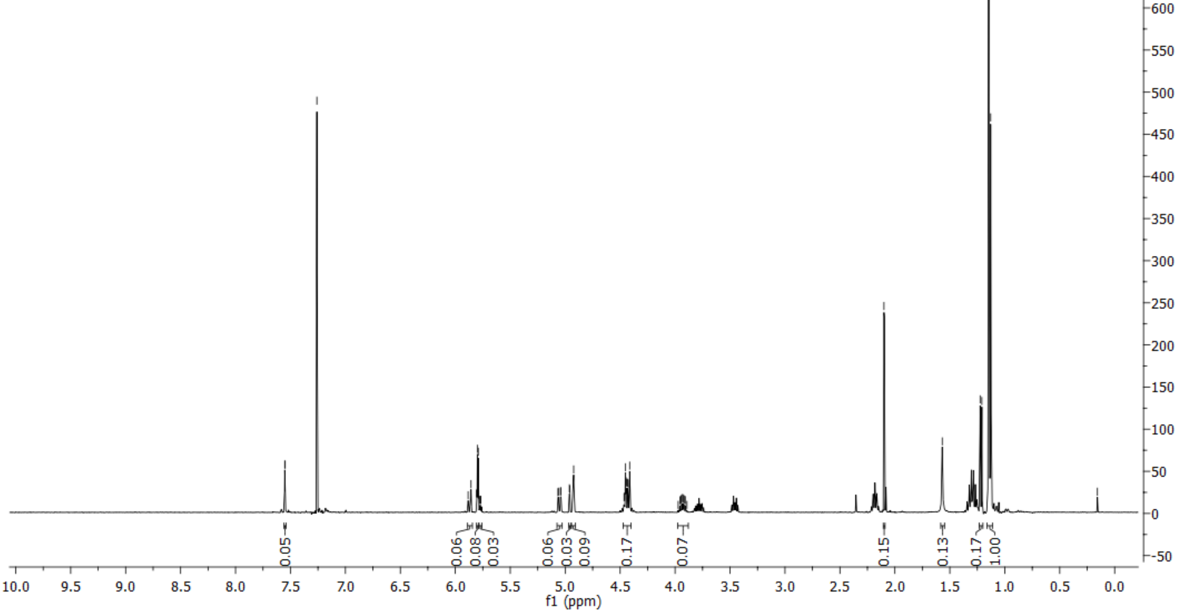
7.55
7.55
7.26
5.88
5.86
5.81
5.80
5.79
5.79
5.78
5.77
5.77
5.04
4.96
4.96
4.92
4.45
4.41
3.97
3.96
3.95
3.94
3.93
3.92
3.92
3.91
3.89
2.10
1.57
1.22
1.21
1.15
1.13
0.16
0.05
0.06
0.08
0.03
0.06
0.03
0.09
0.17
0.07
0.15
0.13
0.17
1.00
f1 (ppm)

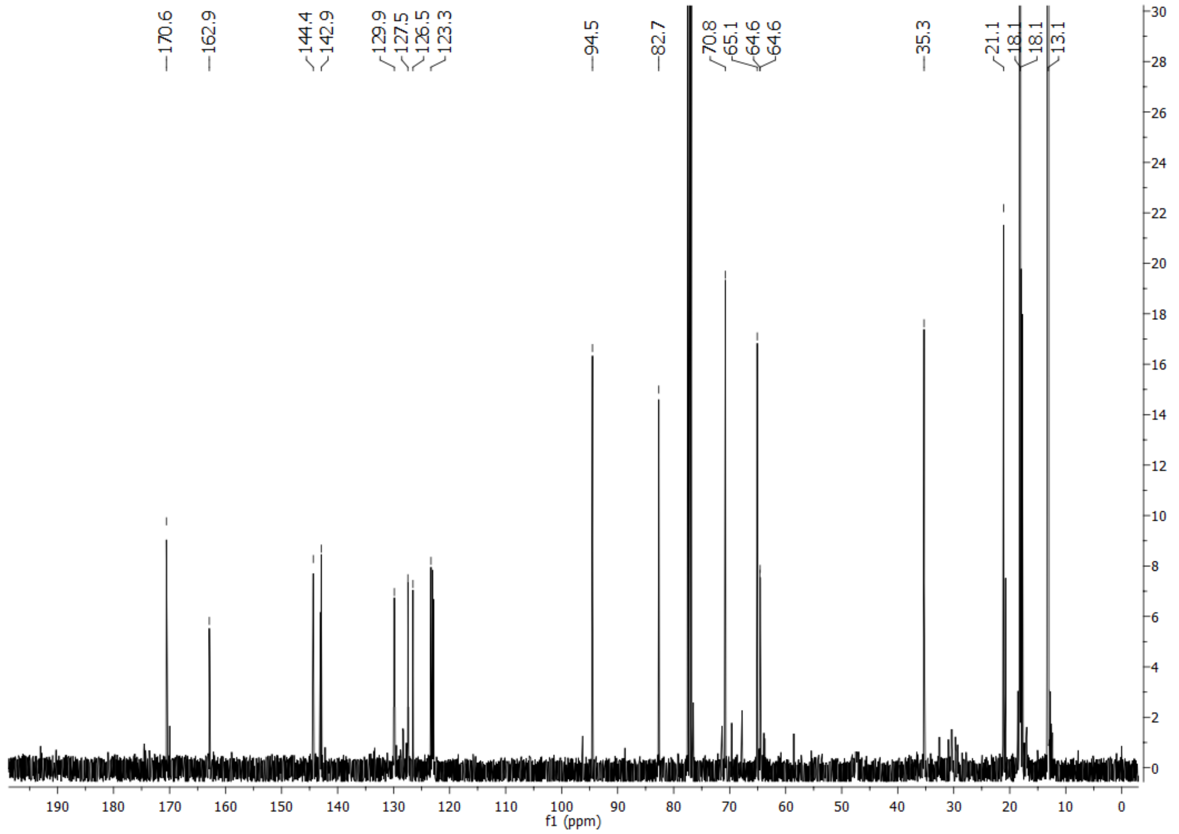
170.6
162.9
144.4
142.9
129.9
127.5
126.5
123.3
94.5
82.7
70.8
65.1
64.6
64.6
35.3
21.1
18.1
18.1
13.1
f1 (ppm)

Figure S1. Survival fraction of HCT116 (left) and MCF7 (right) cell lines after 72-hours of incubation with tested compounds (10, 11, 12, 13, 14, 15, 16, 17, 18, 19, 20, 21, 22, 23, 24, 25, 26, respectively), evaluated by MTT assay. Results presented as mean from 3 experiments, +/- SD. Statistical significance indicated by star; evaluated by T-test, where $p<0.05$ (TRUE bolded under the charts).

10

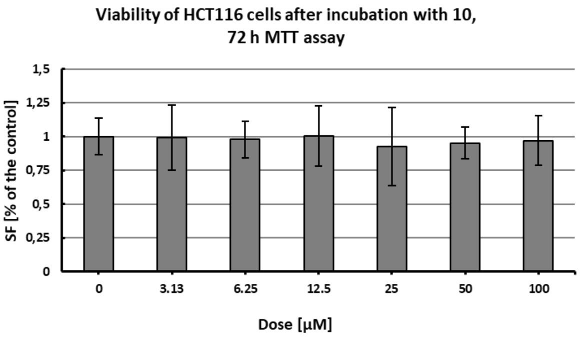

**P value**	0.9608	0.8066	0.9667	0.7228	0.5496	0.8015
**P<0.05**	FALSE	FALSE	FALSE	FALSE	FALSE	FALSE

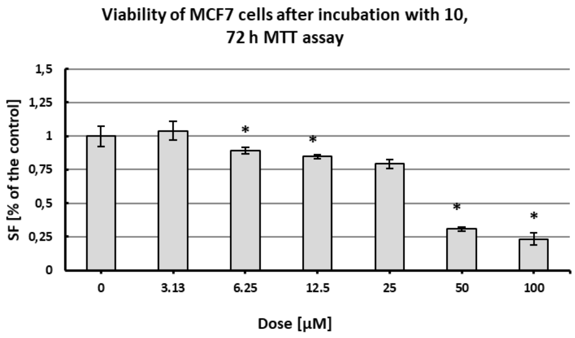

**P value**	0.4348	**0.0132**	**0.0037**	0.0927	**0.0002**	**0.0012**
**P<0.05**	FALSE	TRUE	TRUE	FALSE	TRUE	TRUE

11

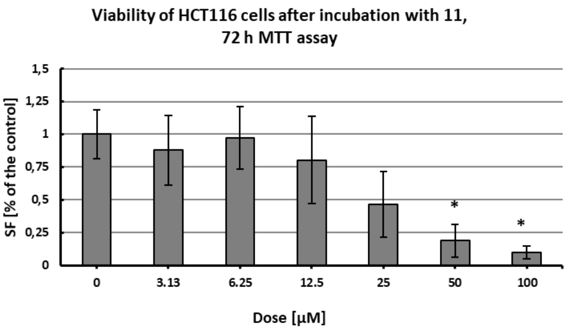

**P value**	0.5077	0.8499	0.4116	0.2352	**0.0079**	**0.001**
**P<0.05**	FALSE	FALSE	FALSE	FALSE	TRUE	TRUE

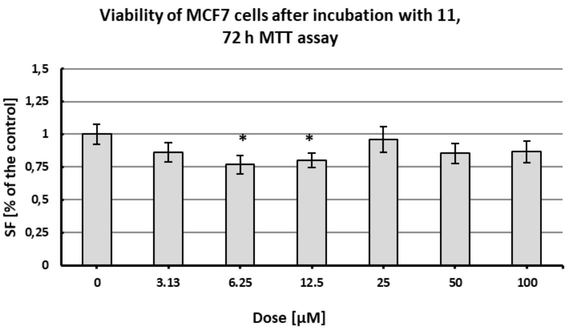

**P value**	0.0791	**0.0288**	**0.0251**	0.0945	0.0835	0.1096
**P<0.05**	FALSE	TRUE	TRUE	FALSE	FALSE	FALSE

12

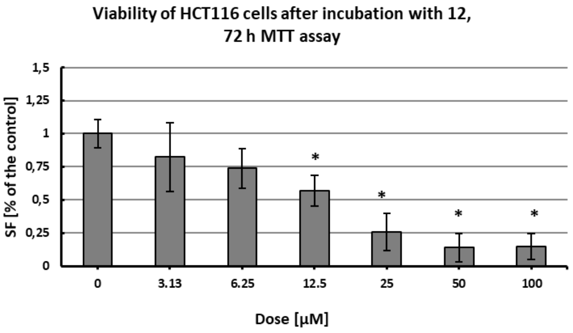

**P value**	0.3575	0.0945	**0.0236**	**0.0449**	**0.0051**	**0.0046**
**P<0.05**	FALSE	FALSE	TRUE	TRUE	TRUE	TRUE

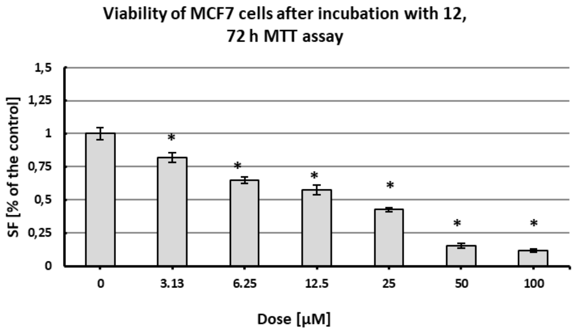

**P value**	**0.012**	**0.0015**	**0.0023**	**0.0085**	**0.0002**	**6.54E-05**
**P<0.05**	TRUE	TRUE	TRUE	TRUE	TRUE	TRUE

13

Viability of HCT116 cells after incubation with 13, 72 h MTT assay

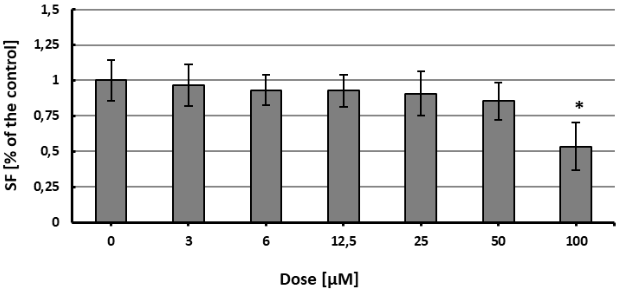

P value	0.7367	0.3819	0.3878	0.8578	0.1933	**0.0404**
**P<0.05**	FALSE	FALSE	FALSE	FALSE	FALSE	TRUE

Viability of MCF7 cells after incubation with 13, 72 h MTT assay

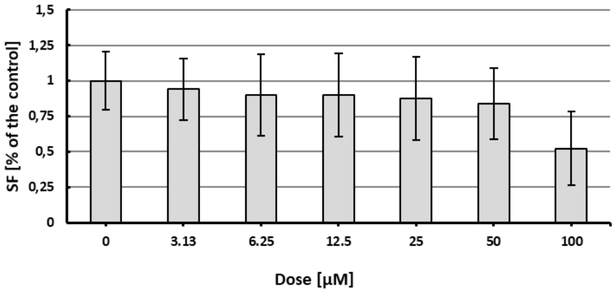

P value	0.6847	0.6091	0.6177	0.9151	0.3798	0.0859
**P<0.05**	FALSE	FALSE	FALSE	FALSE	FALSE	FALSE

14

Viability of HCT116 cells after incubation with 14, 72 h MTT assay

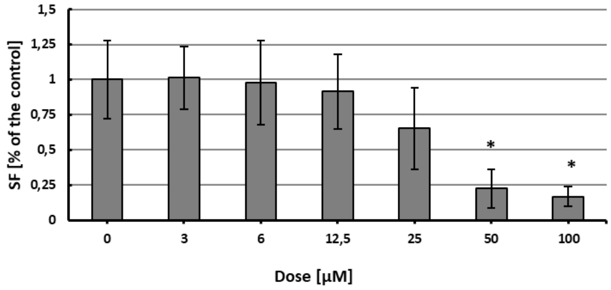

P value	0.9229	0.9153	0.6395	0.3127	**0.0103**	**0.0025**
**P<0.05**	FALSE	FALSE	FALSE	FALSE	TRUE	TRUE

Viability of MCF7 cells after incubation with 14, 72 h MTT assay

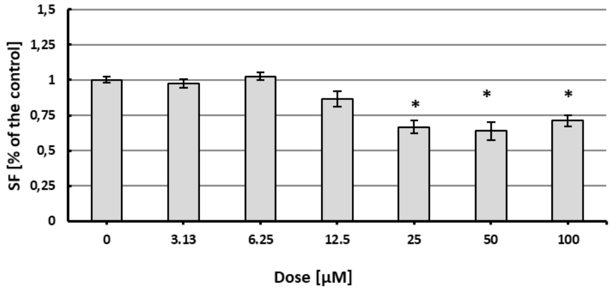

P value	0.3197	0.2292	0.0516	**0.0084**	**0.0102**	**0.0062**
**P<0.05**	FALSE	FALSE	FALSE	TRUE	TRUE	TRUE

15

Viability of HCT116 cells after incubation with 15, 72 h MTT assay

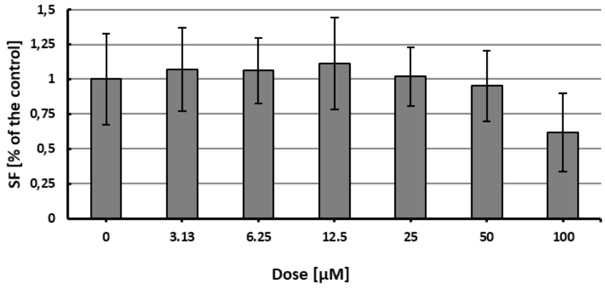

P value	0.7276	0.6992	0.6177	0.7051	0.7771	0.1416
**P<0.05**	FALSE	FALSE	FALSE	FALSE	FALSE	FALSE

Viability of MCF7 cells after incubation with 15, 72 h MTT assay

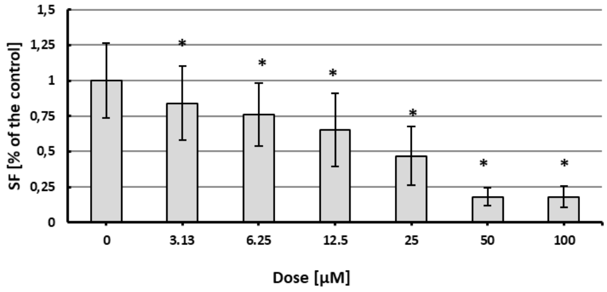

P value	**0.0454**	**0.0032**	**0.0022**	**0.0032**	**0.0003**	**0.0008**
**P<0.05**	TRUE	TRUE	TRUE	TRUE	TRUE	TRUE

16

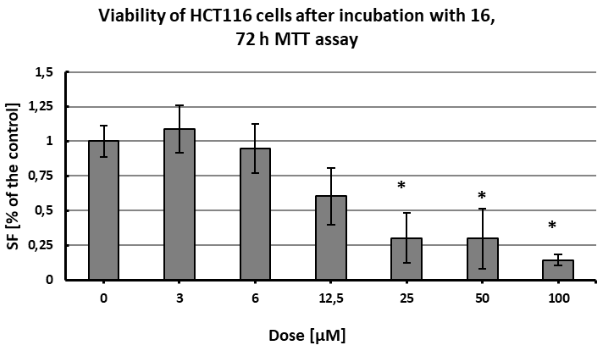

P value	0.4746	0.6621	0.0794	**0.0297**	**0.0308**	**0.0006**
**P<0.05**	FALSE	FALSE	FALSE	TRUE	TRUE	TRUE

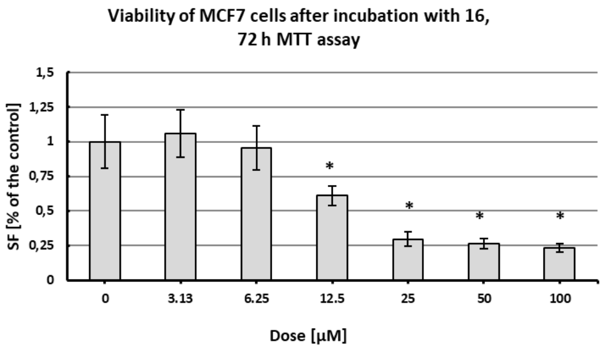

P value	0.605	0.678	**0.0111**	**0.0047**	**0.0008**	**0.0006**
**P<0.05**	FALSE	FALSE	TRUE	TRUE	TRUE	TRUE

17

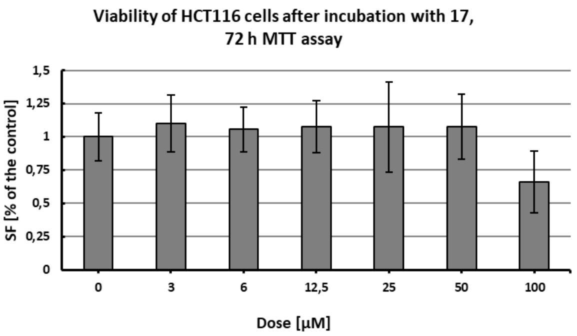

P value	0.4987	0.6298	0.5601	0.9145	0.6436	0.1266
**P<0.05**	FALSE	FALSE	FALSE	FALSE	FALSE	FALSE

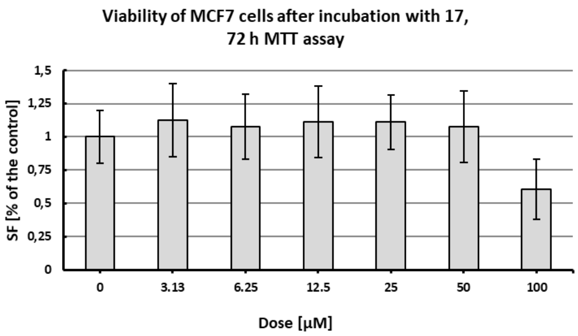

P value	0.5099	0.645	0.5398	0.9943	0.6731	0.0944
**P<0.05**	FALSE	FALSE	FALSE	FALSE	FALSE	FALSE

18

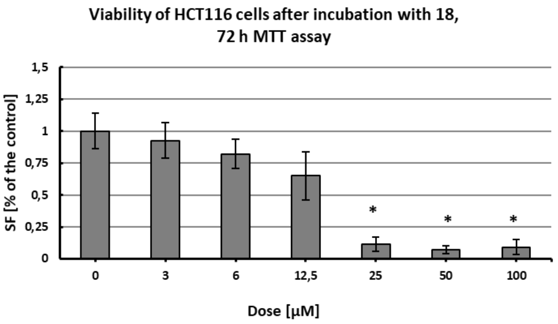

P value	0.4606	0.1127	0.0831	**0.0301**	**0.0004**	**0.0013**
**P<0.05**	FALSE	FALSE	FALSE	TRUE	TRUE	TRUE

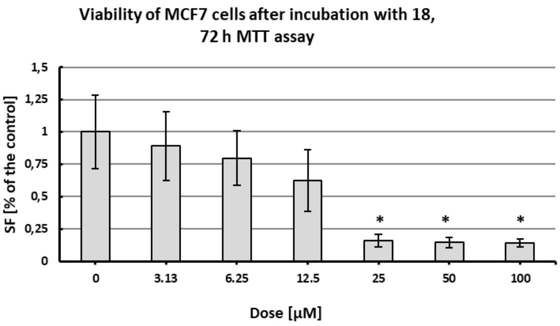

P value	0.5506	0.2357	0.1105	**0.0417**	**0.0006**	**0.0004**
**P<0.05**	FALSE	FALSE	FALSE	TRUE	TRUE	TRUE

19

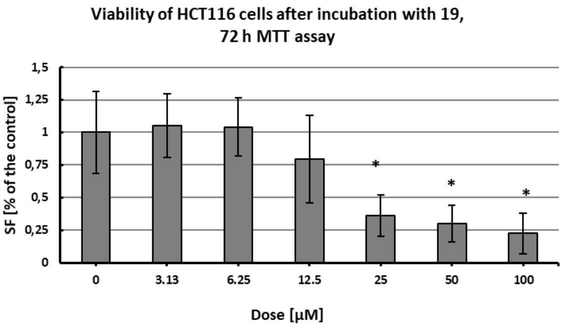

**P value**	0.7433	0.7801	0.398	**0.0414**	**0.0131**	**0.0131**
**P<0.05**	FALSE	FALSE	FALSE	TRUE	TRUE	TRUE

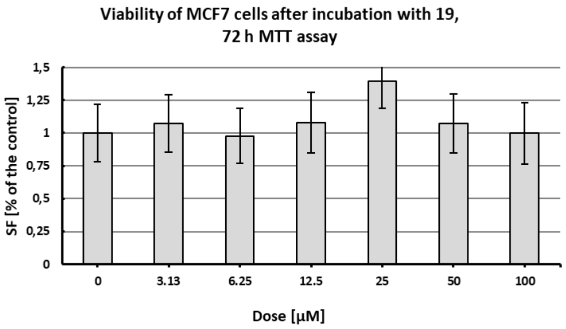

**P value**	0.6316	0.8688	0.6167	0.151	0.6302	0.9866
**P<0.05**	FALSE	FALSE	FALSE	FALSE	FALSE	FALSE

20

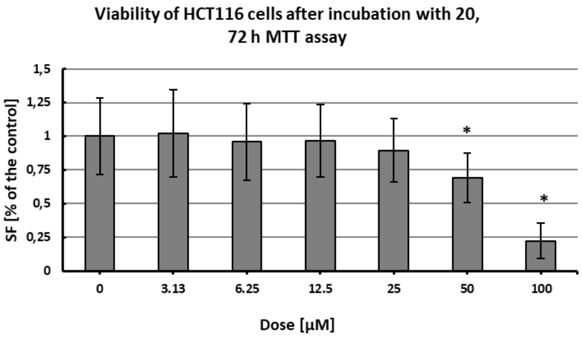

**P value**	0.9207	0.8185	0.8394	0.748	**0.1058**	**0.0094**
**P<0.05**	FALSE	FALSE	FALSE	FALSE	TRUE	TRUE

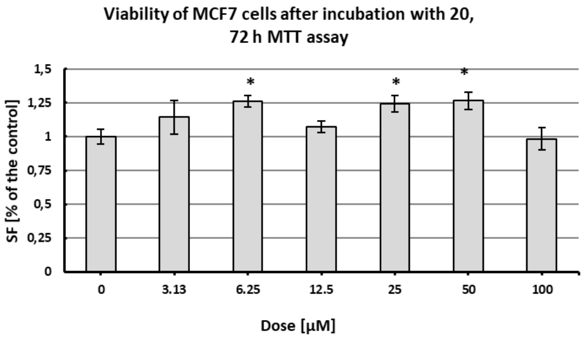

**P value**	0.1818	**0.0083**	0.099	**0.0207**	**0.0197**	0.7542
**P<0.05**	FALSE	TRUE	FALSE	TRUE	TRUE	FALSE

21

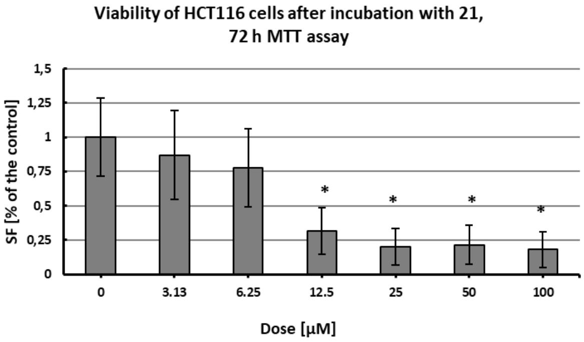

**P value**	0.5579	0.3071	**0.0196**	**0.0114**	**0.0109**	**0.0085**
**P<0.05**	FALSE	FALSE	TRUE	TRUE	TRUE	TRUE

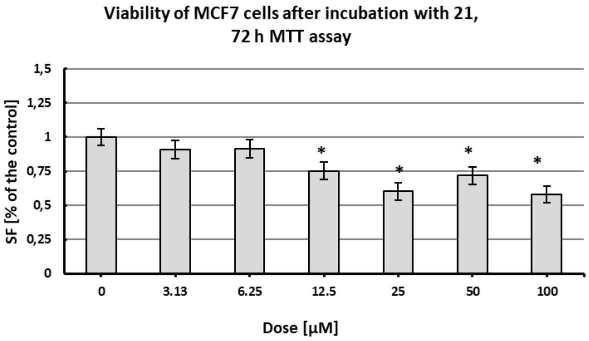

**P value**	0.1321	0.1511	**0.022**	**0.045**	**0.0173**	**0.0072**
**P<0.05**	FALSE	FALSE	TRUE	TRUE	TRUE	TRUE

<u>**22**</u>

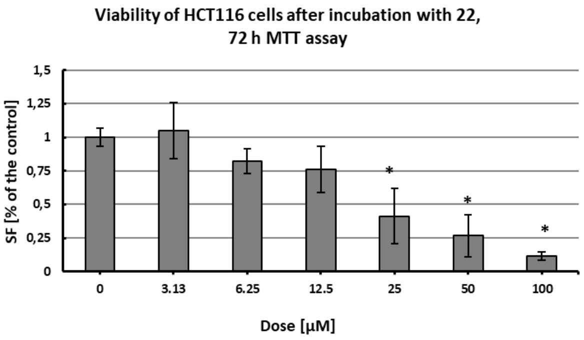

**P value**	0.7185	0.0759	0.1409	**0.0498**	**0.0149**	**0.0004**
**P<0.05**	FALSE	FALSE	FALSE	TRUE	TRUE	TRUE

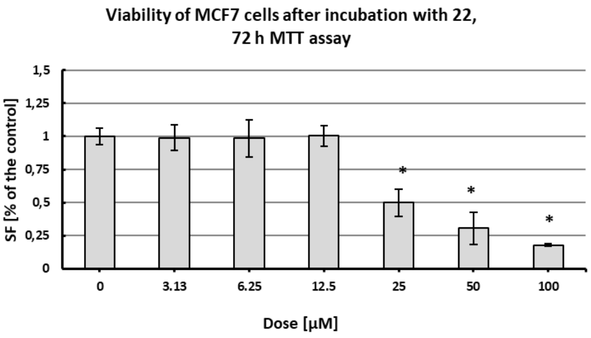

**P value**	0.8503	0.8719	0.9481	**0.0029**	**0.0101**	**4.21E-05**
**P<0.05**	FALSE	FALSE	FALSE	TRUE	TRUE	TRUE

<u>**23**</u>

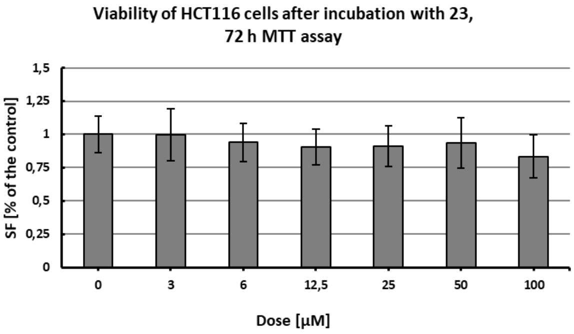

**P value**	0.9684	0.5371	0.3541	0.951	0.6101	0.2154
**P<0.05**	FALSE	FALSE	FALSE	FALSE	FALSE	FALSE

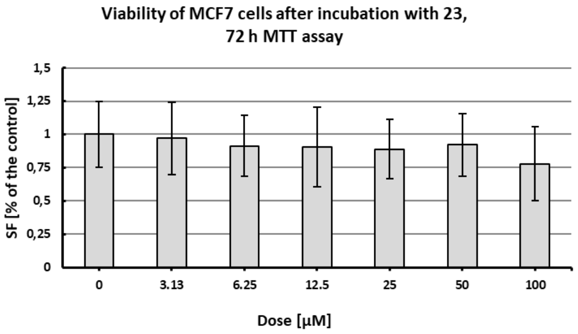

**P value**	0.8643	0.5811	0.6396	0.9455	0.6244	0.3031
**P<0.05**	FALSE	FALSE	FALSE	FALSE	FALSE	FALSE

<u>**24**</u>

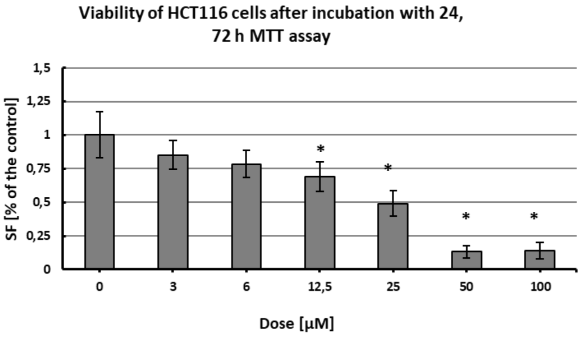

**P value**	0.1375	0.0636	**0.0406**	**0.0474**	**0.0009**	**0.0017**
**P<0.05**	FALSE	FALSE	TRUE	TRUE	TRUE	TRUE

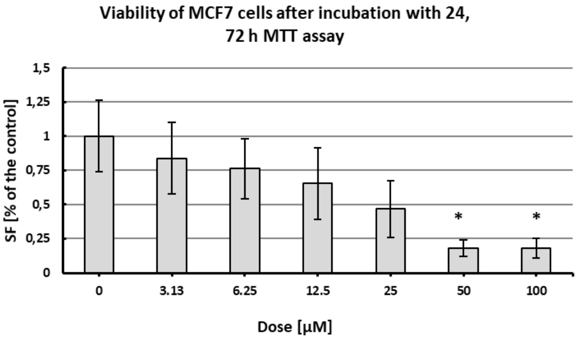

**P value**	0.3975	0.2038	0.1462	0.0905	**0.0019**	**0.0027**
**P<0.05**	FALSE	FALSE	FALSE	FALSE	TRUE	TRUE

25

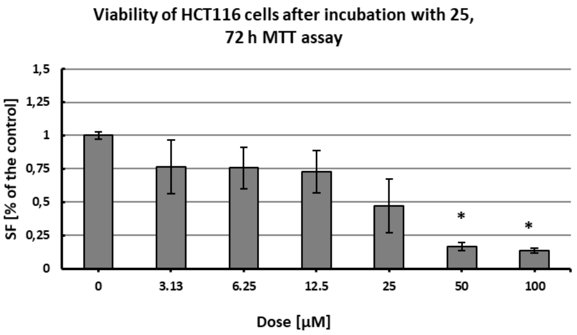

P value	0.1813	0.1142	0.1001	0.1649	**0.0005**	**0.0001**
**P<0.05**	FALSE	FALSE	FALSE	FALSE	TRUE	TRUE

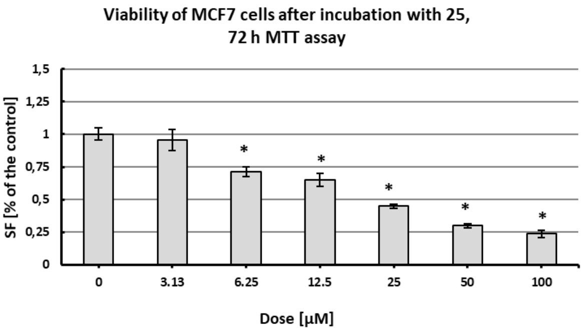

P value	0.4322	**0.0056**	**0.0063**	**0.0134**	**0.0002**	**0.0004**
**P<0.05**	FALSE	TRUE	TRUE	TRUE	TRUE	TRUE

26

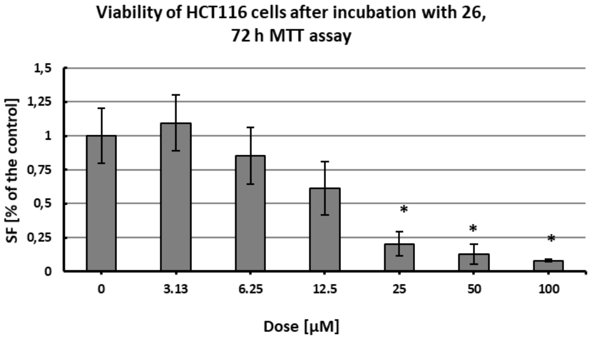

P value	0.5142	0.,3536	0.0764	**0.0509**	**0.0023**	**3.42E-05**
**P<0.05**	FALSE	FALSE	FALSE	TRUE	TRUE	TRUE

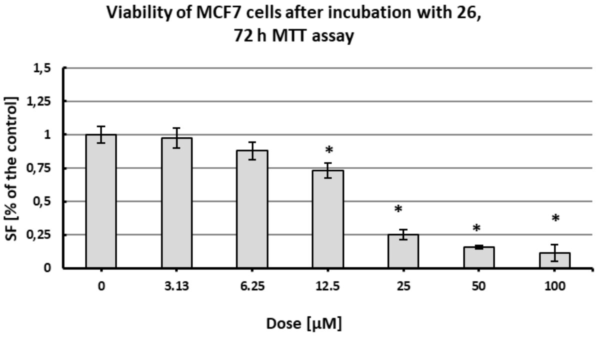

P value	0.6008	0.0824	**0.015**	**0.0006**	**9.01E-05**	**0.0015**
**P<0.05**	FALSE	FALSE	TRUE	TRUE	TRUE	TRUE
